# Transmembrane Transport of cAMP and AMP Using a Two Component Small Molecule Transport System

**DOI:** 10.1002/anie.202524663

**Published:** 2025-12-09

**Authors:** Uththara M.C. Rathnaweera, Olivia Sam, Karolis Norvaisa, Sarah R. Marshall, Randima D. De Silva Weerakonda Arachchige, Matúš Chvojka, Hennie Valkenier, Nathalie Busschaert

**Affiliations:** ^1^ Department of Chemistry Tulane University 6400 Freret St New Orleans LA 70118 USA; ^2^ Engineering of Molecular NanoSystems École Polytechnique de Bruxelles Université Libre de Bruxelles Avenue F.D. Roosevelt 50, CP165/64 Brussels 1050 Belgium; ^3^ Department of Chemistry Faculty of Science Masaryk University Kamenice 5 Brno 625 00 Czech Republic

**Keywords:** Anionophore, cAMP, Nucleotide, Supramolecular chemistry, Transmembrane transport

## Abstract

Nucleotides such as cAMP (cyclic adenosine monophosphate) and AMP (adenosine monophosphate) are central to many cellular processes, but their highly hydrophilic and charged nature prevents passive permeation across lipid bilayers. Here, we report the first example of facilitated transport of cAMP and AMP across liposome membranes using a neutral two‐component system at physiological pH. This system pairs a synthetic anionophore targeting the phosphate group with a thymine derivative to boost transport efficiency. Liposome‐based fluorescence and ^31^P NMR experiments confirmed transmembrane transport, supported by control experiments. A fluorinated squaramide proved to be the best transporter and was able to transport cAMP even without the help of a thymine derivative, as well as AMP in the presence of a lipophilic thymine derivative. These findings show that carefully designed small molecules can enable direct nucleotide translocation, with potential applications in drug delivery and synthetic biology.

## Graphical abstract

Transmembrane transport of the nucleotides cAMP (cyclic adenosine monophosphate) and AMP (adenosine monophosphate) is challenging, but can be achieved using a two‐component system consisting of a synthetic anionophore (e.g., squaramides) and a lipophilic thymine derivative. Fluorescence and NMR‐based liposomes studies confirm that direct translocation of the nucleotides across the membrane occurs without disruption of membrane integrity.

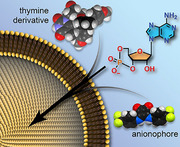

In the last few decades, supramolecular chemists have developed synthetic systems that can transport ions and other polar moieties across biological membranes.^[^ ^1^, ^2^ ^]^ While synthetic Clˉ transporters have been widely explored for applications in treating cystic fibrosis, cancer, and bacterial infections,^[^ ^3^ ^]^ nucleotide transport has received far less attention—despite being some of the most interesting anions in biology. Beyond serving as the building blocks of DNA and RNA, nucleotides play a central role in energy transfer, signal transduction, and metabolism. In this regard, ATP (adenosine triphosphate) and cAMP (cyclic adenosine monophosphate) are well‐known as the energy currency and the main secondary messenger of the cell, respectively,^[^ ^4^, ^5^ ^]^ but other nucleotides such as AMP (adenosine monophosphate), ADP (adenosine diphosphate), GDP (guanosine diphosphate) and GTP (guanosine triphosphate) also have important biological functions.^[^ ^6^, ^7^, ^8^, ^9^ ^]^ Nucleotides and nucleotide‐analogues also play an important role in therapeutic contexts. Elevated intracellular cAMP levels have been shown to inhibit leukemia cell proliferation,^[^ ^10^ ^]^ and many antiviral and anticancer drugs are nucleotide analogues (e.g., acyclovir, gemcitabine, tenofovir).^[^ ^11^, ^12^, ^13^, ^14^, ^15^ ^]^ However, because nucleotides cannot cross the membrane passively and there are no nucleotide transport proteins in the plasma membrane, the bioavailability of these drugs is very low.^[^ ^11^ ^]^ In most cases, neutral nucleosides or analogues are used as drugs that are phosphorylated inside the body (e.g., acyclovir).^[^ ^16^ ^]^ Alternatively, lipophilic phosphoesters or phosphoramidates are used as prodrugs, which are hydrolyzed inside the cell by various enzymes (e.g., tenofovir).^[^ ^17^, ^18^, ^19^ ^]^ However, the initial phosphorylation step is very slow and prodrug‐to‐drug conversions always have a risk of interpatient variability and the release of toxic byproducts.^[^ ^20^ ^]^ The direct delivery of these drugs in their monophosphate form could therefore be an alternative strategy.

Even with these strong incentives, transporting nucleotides across lipid membranes is a major challenge due to their large size, multiple negative charges, and the high hydration energy of phosphate anions.^[^ ^21^, ^22^ ^]^ Most strategies thus rely on nanoparticles^[^ ^23^, ^24^ ^]^ or polycationic systems,^[^ ^25^, ^26^, ^27^, ^28^, ^29^, ^30^, ^31^ ^]^ which generally function by facilitated endocytosis.^[^ ^32^ ^]^ An anionophore approach, whereby a neutral small molecule binds and shuttles the nucleotide across the membrane, has not yet been reported. However, early examples of small molecules capable of transporting cAMP and other nucleotides across bulk organic layers in U‐tube experiments^[^ ^33^, ^34^, ^35^, ^36^, ^37^, ^38^, ^39^, ^40^ ^]^ suggest that this approach should be feasible. In this manuscript, we thus report the first neutral carriers that can facilitate the direct translocation of monophosphate nucleotides across phospholipid bilayers.

As proof‐of‐principle, we focused on adenine nucleotides (cAMP, AMP, ADP and ATP), as they have the most varied biological functions. Since their negative charge resides on the phosphate groups, effective transporters must bind and shield these phosphates from the hydrophobic interior of the membrane. We envisioned that *tren*‐based (thio)ureas (**1**–**4**, Scheme 1) would be good starting points for the development of synthetic nucleotide transporters, as they have previously been shown to transport sulfate and phosphoesters.^[^ ^41^, ^42^ ^]^ We also included mono‐(thio)ureas and ‐squaramides (**5**–**10**, Scheme 1) because they can easily be obtained via 1‐step synthesis and have previously been shown to be good anion transporters.^[^ ^43^ ^]^ To enhance cAMP and AMP transport, we also explored a two‐component transport system.^[^ ^44^ ^]^ Nucleotides consist of phosphate groups attached to a nucleoside (sugar + nucleobase). Given that nucleosides are taken up into the cell by specialized nucleoside transport proteins,^[^ ^45^, ^46^ ^]^ we hypothesized that the nucleoside moiety might hinder transport by **1**–**10**. To overcome this, we introduced lipophilic thymine derivatives (**Me‐T** and **DMT‐dT**) as co‐transporters, expected to base pair with the adenine nucleobase (Scheme 1). All compounds were either commercially sourced or synthesized via previously reported methods.^[^ ^43^, ^47^ ^]^

**Scheme 1:**
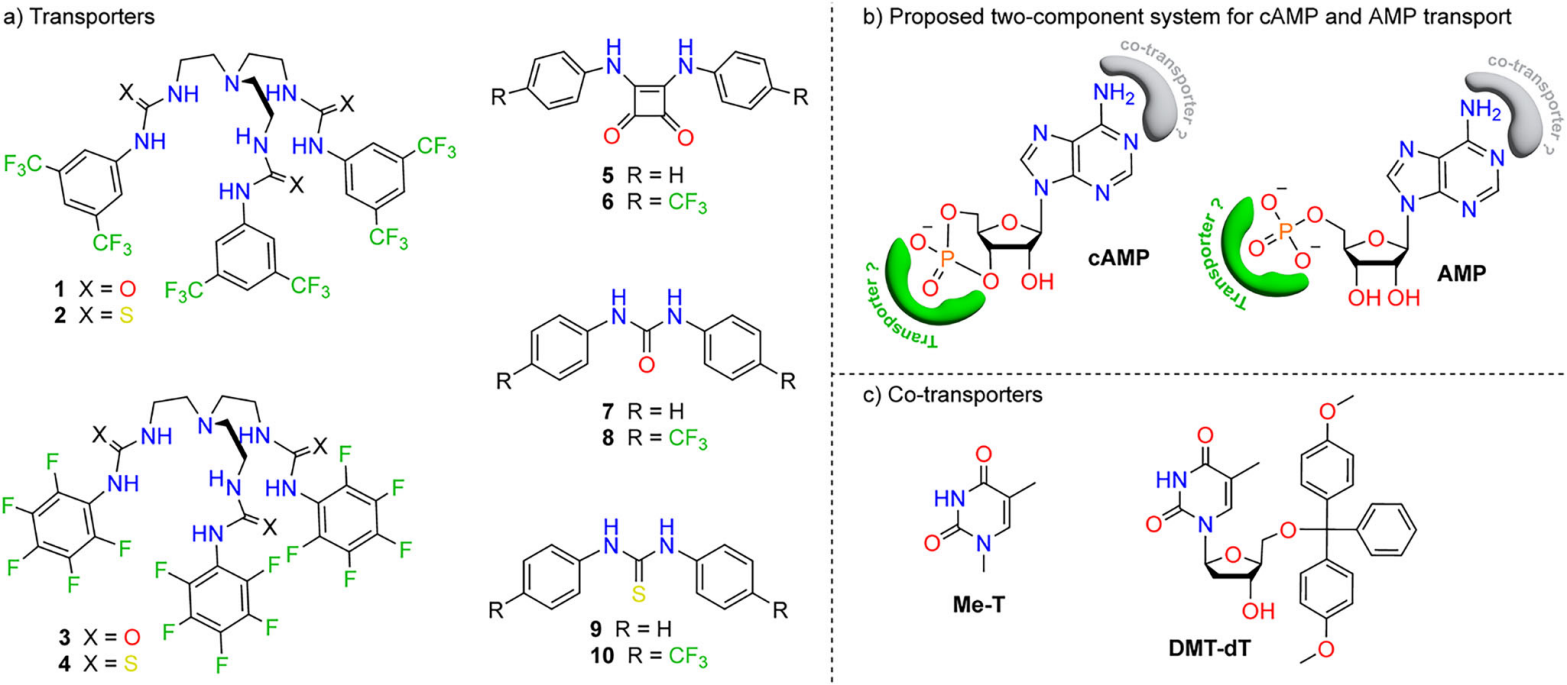
a) Structures of transporters **1**–**10**, b) Structure of cAMP and AMP with the putative binding sites for the transporters and co‐transporters indicated in green and grey respectively, c) Structures of co‐transporters **Me‐T** and **DMT‐dT**.

Typical anion transport assays often use lucigenin as the fluorescent dye, but this dye is relatively membrane permeable and known to slowly leak out of liposomes, risking false positives.^[^ ^48^ ^]^ To avoid this, we used a more hydrophilic analogue of lucigenin (SPBA),^[^ ^48^ ^]^ which does not show leakage out of liposomes and which fluorescence is efficiently quenched by cAMP and AMP (Figures S6–S9). We initially focused on screening cAMP transport, as it has a single negative charge at physiological pH and should therefore be easier to transport across liposome membranes than the other nucleotides. Still, we anticipated that cAMP transport would be challenging and that high transporter/co‐transporter concentrations would be required, as well as long transport times. Initial assays in 200 nm POPC (1‐palmitoyl‐2‐oleoyl‐glycero‐3‐phosphocholine) large unilamellar vesicles (LUVs) were therefore conducted using the highest concentrations that did not result in precipitation (3 mol% of transporter and 10 mol% of co‐transporter with respect to lipid, see Figures S10–S13). The results are shown in Figure 1.

**Figure 1:**
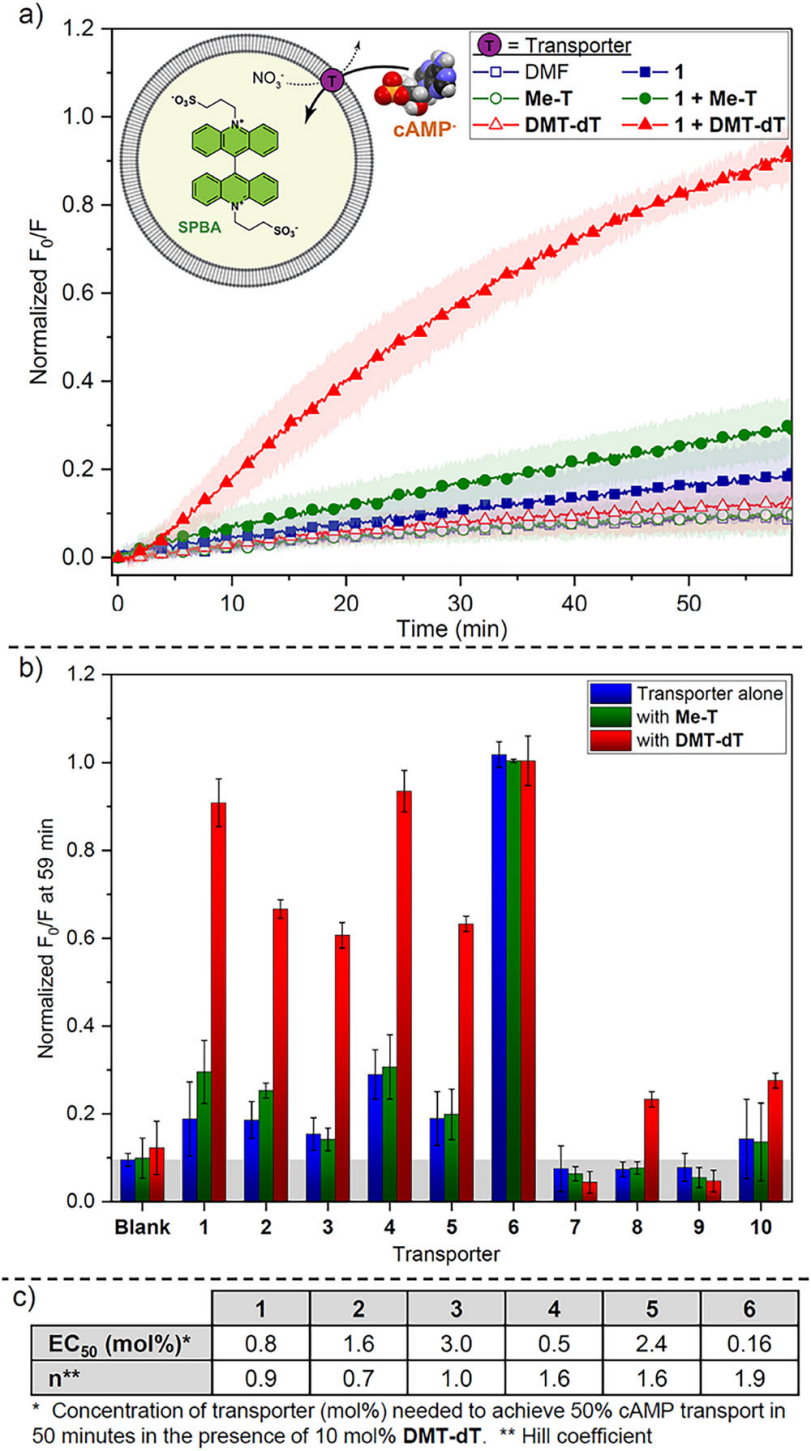
cAMP transport facilitated by a two‐component system. Experiments were performed with 200 nm POPC LUVs containing 0.8 mM SPBA, 225 mM NaNO_3_, 10 mM HEPES at pH 7.4 and suspended in a solution of 25 mM cAMP sodium salt, 225 mM NaNO_3_, 10 mM HEPES at pH 7.4. a) Representative normalized cAMP transport curves for transporter **1** (3 mol%) with and without 10 mol% co‐transporter. b) Bar chart showing cAMP transport mediated by transporters **1–10** (3 mol%) with and without co‐transporters (10 mol%). Data represents normalized fluorescence intensity changes measured 59 minutes after the addition of transporter and is the average of minimum 3 independent repeats, with error bars representing standard deviations. Greyed out area indicates no transport. c) Table of the EC_50_ values (mol%) and Hill coefficients (*n*) of transporters **1–6**, corresponding to the concentration needed to achieve 50% cAMP transport in 50 min in the presence of 10 mol% **DMT‐dT**.

From Figure 1 several trends can be observed. First, fluorinated squaramide **6** is the only compound that can transport cAMP without a co‐transporter and is therefore the most potent transporter. Second, *tren*‐based compounds **1**–**4** and unfluorinated squaramide **5** are only able to mediate cAMP transport in the presence of **DMT‐dT** as the co‐transporter, but not in the presence of **Me‐T**. The enhanced activity in the presence of **DMT‐dT** is most likely due to its bulky, highly lipophilic dimethoxytrityl (DMT) group that is more effective at shielding the highly polar cAMP molecule. Finally, transporters **7**–**10** showed limited cAMP transport even in the presence of co‐transporters, indicating that simple mono‐ureas and mono‐thioureas are not effective for transporting the highly hydrophilic cAMP anion.

To assess whether cAMP transport can be achieved at lower concentrations, dose‐response studies were conducted to determine *EC_50_* values (concentration needed to achieve 50% cAMP influx after 50 minutes). The results are shown in Figure 1c and Table S2. In the absence of co‐transporter, squaramide **6** already has a reasonable *EC_50_* value of 0.8 mol% with respect to lipid. This *EC_50_* value drops down to 0.16 mol% in the presence of 10 mol% **DMT‐dT**, the lowest of all *EC_50_* values measured. In the presence of 10 mol% **DMT‐dT**, transporters **1–5** have *EC_50_* values in the range of 0.5–3.0 mol%. These results indicate that most transporters retain activity at concentrations below 1 mol%. This is in stark contrast with the results for varied **DMT‐dT** concentrations when the transporter concentration is maintained at 3 mol% (Table S2). The *EC_50_* values were generally 6 mol% or higher and transport activity drops rapidly as the concentration of the **DMT‐dT** co‐transporter is reduced.

Next, we investigated whether the transporters could also facilitate the translocation of more highly charged nucleotides, such as AMP. At pH 7.4, approximately 90% of AMP is present in its di‐anionic form (Figures S2 and S3), which should be more challenging to transport than cAMP. Only transporters **1**–**6** with or without **DMT‐dT** as a co‐transporter were tested, as transporters **7**–**10** and co‐transporter **Me‐T** did not show any activity with cAMP and were therefore unlikely to be effective. The experiments were carried out similar to the cAMP experiments, using 3 mol% transporter with or without 10 mol% **DMT‐dT** with respect to lipid. The results are shown in Figure 2. As expected, AMP proved to be much more difficult to transport and compounds **1**–**5** did not show any activity, even in the presence of **DMT‐dT**. On the other hand, squaramide **6** was able to transport AMP in the presence of 10 mol% **DMT‐dT** at pH 7.4 as well as pH 8.4 (where nearly all AMP is dianionic; see data in Figure S44). This contrasts with the cAMP transport results, where squaramide **6** was able to transport cAMP in the absence of a co‐transporter. This confirms that squaramide **6** is the best nucleotide transporter of the series, and that AMP is more challenging to transport than cAMP.

**Figure 2:**
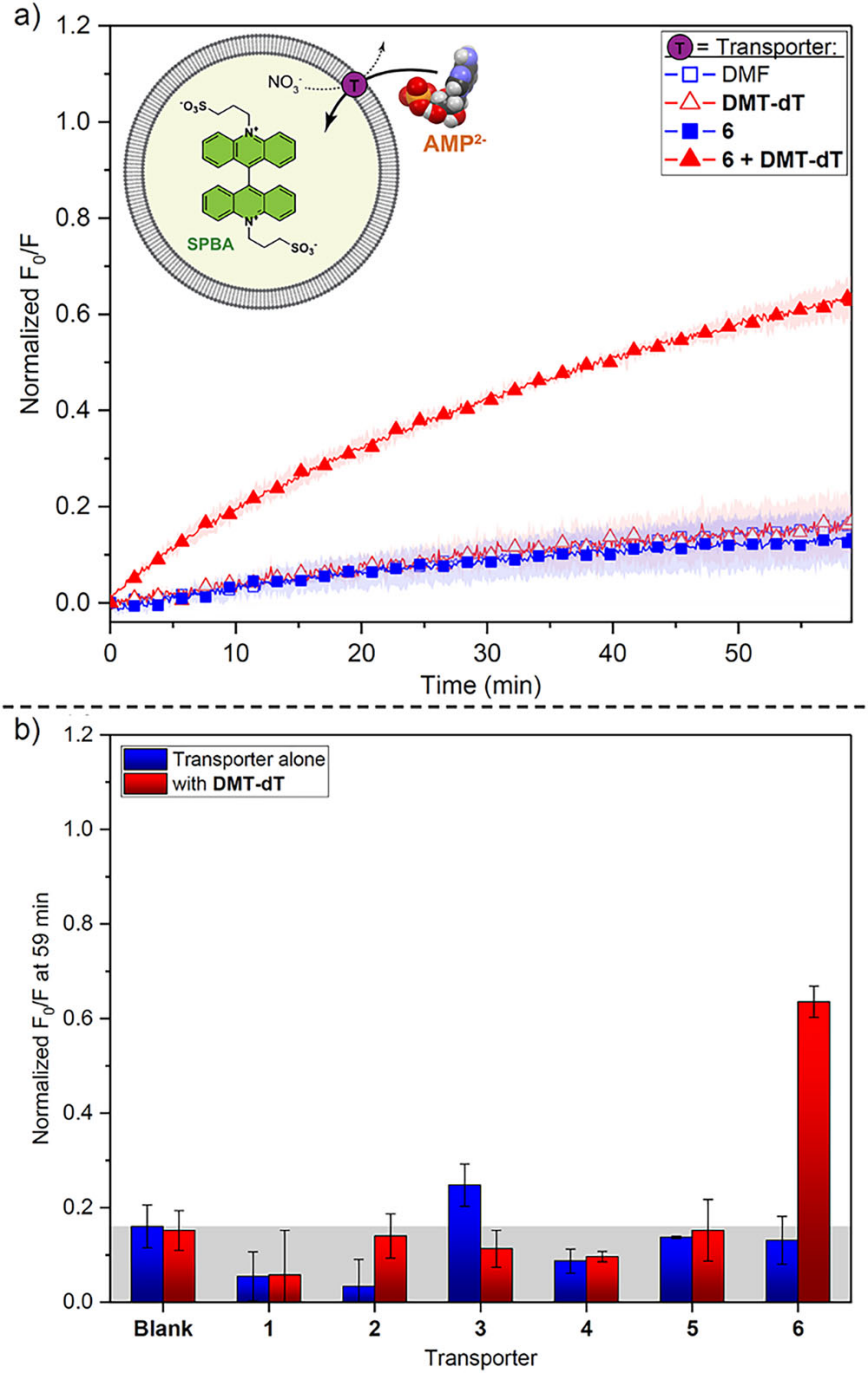
AMP transport facilitated by a two‐component system. Experiments were performed with 200 nm POPC LUVs containing 0.8 mM SPBA, 225 mM NaNO_3_, 10 mM HEPES at pH 7.4 and suspended in a solution of 25 mM AMP sodium salt, 225 mM NaNO_3_, 10 mM HEPES at pH 7.4. a) Representative normalized AMP transport curves of transporter **6** (3 mol%) with and without 10 mol% **DMT‐dT**. b) Bar chart showing AMP transport mediated by transporters **1–6** (3 mol%) with and without **DMT‐dT** (10 mol%). Data represents normalized fluorescence intensity changes measured 59 min after the addition of transporter and is the average of minimum 3 independent repeats, with error bars representing standard deviations. Greyed out area indicates no transport.

The SPBA assay results described above strongly suggest that cAMP and AMP transport facilitated by neutral small molecules is feasible. However, like any assay, false positives are possible and caution should be used when interpreting results. To provide further evidence of cAMP and AMP transport, ^31^P NMR experiments were developed similar to the NMR‐based liposome experiments previously used for chloride,^[^ ^49^ ^]^ bicarbonate,^[^ ^50^ ^]^ sulfate,^[^ ^41^ ^]^ phosphate,^[^ ^51^ ^]^ and amino acid^[^ ^52^ ^]^ transport. In these assays, POPC LUVs are prepared and suspended in a buffer containing 100 mM cAMP or AMP. Transporter and co‐transporter are subsequently added and the solution is stirred for 1 h, after which a ^31^P NMR spectrum is collected. Mn^2+^ is added to the NMR sample as a membrane impermeable paramagnetic agent that can fully relax the ^31^P signal of extravesicular cAMP or AMP. Therefore, a ^31^P NMR signal will only be observed if transport of cAMP or AMP into the LUVs occurred. The results are shown in Figure 3 and align well with the results from the SPBA assays. For cAMP, clear ^31^P NMR signals were observed at –1.56 ppm for all transporters in the presence of **DMT‐dT**, in agreement with the fluorescence data. In contrast, for AMP a clear signal at 3.91 ppm (corresponding to internal AMP) was only observed for the two‐component system of **6** + **DMT‐dT**, with much smaller peaks seen for the other systems, also in agreement with the fluorescence data (see Figures S52, S53 for integration values). An independent ^31^P NMR assay using Eu^3+^ as a shift reagent instead of Mn^2+^ as a paramagnetic agent gave very similar results (Figure S56). Additional ^31^P NMR experiments with ADP and ATP using **6** + **DMT‐dT** showed only very small signals for ADP and no signal for ATP (Figures S54 and S55), indicating that the two‐component system is not effective for transporting the more highly charged ADP and ATP anions.

**Figure 3:**
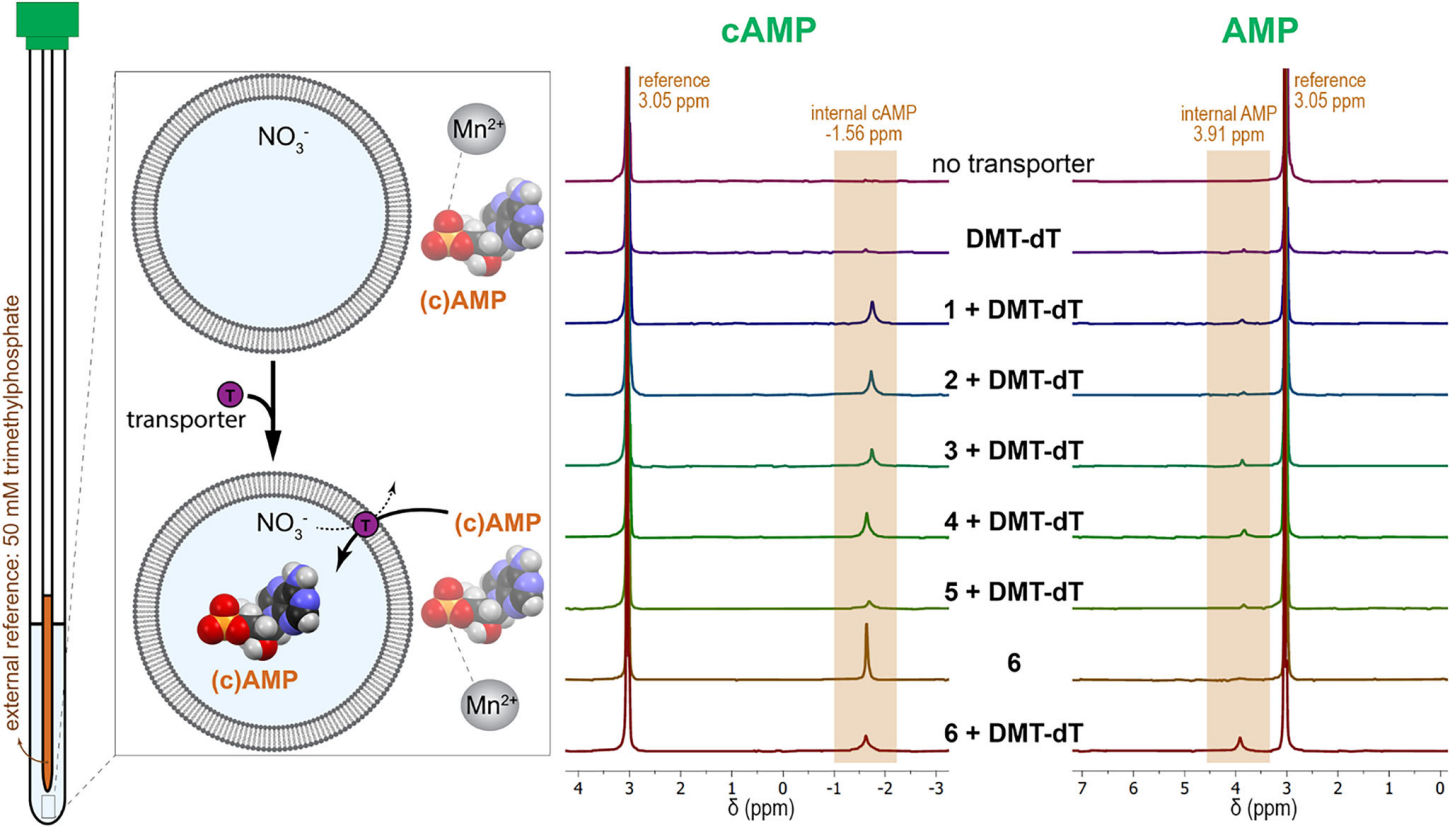
^31^P NMR experiments provide direct evidence of cAMP and AMP transport. 200 nm POPC LUVs in NaNO_3_ buffer at pH 7.4 are spiked with an external solution containing cAMP or AMP, and ^31^P NMR spectra are obtained 1 h after the addition of transporter. The spectra are obtained in the presence of MnSO_4_ to relax any external cAMP or AMP signal. The spectra shown contained the following transporters (from top to bottom): no transporter, **DMT‐dT** (10 mol%), transporter **1** (3 mol%) with **DMT‐dT** (10 mol%), transporter **2** (3 mol%) with **DMT‐dT** (10 mol%), transporter **3** (3 mol%) with **DMT‐dT** (10 mol%), transporter **4** (3 mol%) with **DMT‐dT** (10 mol%), transporter **5** (3 mol%) with **DMT‐dT** (10 mol%), transporter **6** (3 mol%), and transporter **6** (3 mol%) with **DMT‐dT** (10 mol%). All spectra are shown at the same vertical scale.

To understand why squaramide **6** outperforms the other transporters, we investigated whether differences in cAMP binding affinity could explain its superior activity. ^1^H NMR titrations were carried out for squaramide **6** and *tren*‐based urea **1** in DMSO‐*d_6_* containing 0.5% water. Additions of aliquots of cAMP led to downfield shifts of the NH peaks of **1** and **6** (Figure 4a), allowing the determination of association constants using Bindfit2.^[^ ^53^ ^]^ For transporter **1**, the data fitted well to a 1:1 binding model with *K_a_* = 1300 M^−1^ (error 6%). However, for transporter **6** the data did not conform to a 1:1 binding model, and a better fit was obtained using a 2:1 host‐guest binding model (two transporter molecules per cAMP anion), with *K*_a1_ = 1050 (error 7%) and *K*_a2_ = 300 (error 35%) (see Figure S59). Interestingly, the Hill coefficient obtained for the concentration‐dependent transport experiments for **6** was ∼2 (see Figure 1c), suggesting that the 2:1 stoichiometry might also play a role during the transport of cAMP facilitated by **6**. Although the association constants for cAMP are similar for **6** and **1**, the 2:1 stoichiometry might provide better shielding of the polar phosphate from the membrane. Molecular mechanics (MM) conformational searches confirmed that these stoichiometries are possible and show that in each case the adenine nucleobase remains free for interactions with the co‐transporter (Figure 4b and Section S9).

**Figure 4:**
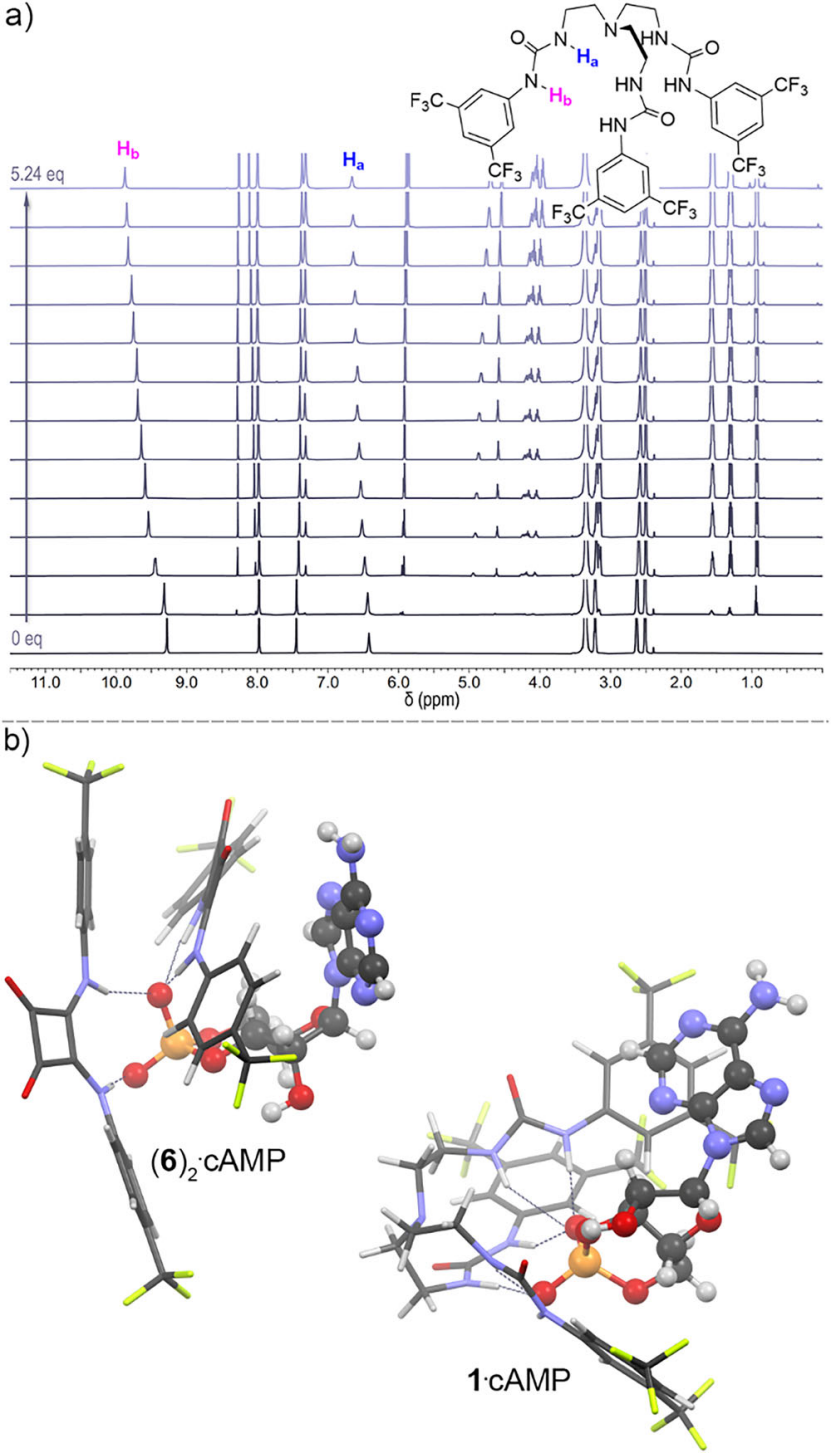
a) Stack plot of the ^1^H NMR titration of aliquots of cAMP (added as a tetrabutylammonium salt) into a solution of compound **1** in DMSO‐d_6_ containing 0.5% water. b) MM model of compound **1** interacting with cAMP with a 1:1 stoichiometry (right) and compound **6** interacting with cAMP with a 2:1 stoichiometry (left). cAMP molecules are shown in ball‐and‐stick mode, and transporters are shown as capped sticks.

The final question we wanted to address was whether **DMT‐dT** acts as a true co‐transporter or merely as a membrane disruptor. Calcein leakage assays eliminated the possibility that **DMT‐dT** functions as a detergent (Figure S66), and experiments using DPPC (dipalmitoylphosphatidylcholine) above and below its phase transition temperature suggested that the formation of transmembrane channels is also unlikely (Figure S67). Furthermore, liposome‐based measurements of membrane fluidity using Laurdan and 1,6‐diphenyl‐1,3,5‐hexatriene (DPH) indicated that 10 mol% **DMT‐dT** only has a negligible effect on membrane fluidity (Figure S68 and S69). Combined, these results support a co‐transport mechanism, whereby **DMT‐dT** interacts with the cAMP‐transporter complex, rather than changes in membrane properties. However, the exact nature of the transporter/co‐transporter/nucleotide complex is still unclear. Given the large number of hydrogen bond donors and acceptors in all components, the complex may differ from the idealized model in Scheme 1. Indeed, MM modelling of the complexes between cAMP or AMP with transporters **1** or **6** and **DMT‐dT** often showed π–π stacking between **DMT‐dT** and the adenine base or between **DMT‐dT** and the transporter, instead of canonical base‐pairing (Figure S60–S65). To try to differentiate between transporter/co‐transporter interactions and cAMP/co‐transporter interactions, experiments using alternative co‐transporters (**DMT‐dA**, **DMT‐dG**, **DMT‐dC**) were conducted. These revealed different rates of cAMP transport for different co‐transporters, with the highest rate generally observed for **DMT‐dT** and the lowest rate for **DMT‐dG** (Figure 5 and Figure S84–S90), indicating some degree of base selectivity. Still, in a membrane environment, single non‐preorganised nucleobases may form alternative hydrogen bonds, limiting selectivity compared to DNA.^[^ ^54^, ^55^, ^56^ ^]^ We therefore tested the effect of **DMT‐dT** on the transport of other anions, such as inorganic phosphate and chloride. The transport of H_2_PO_4_ˉ by compounds **2** and **6** was clearly enhanced in the presence of **DMT‐dT** (Figure S83). For the transport of Clˉ by *tren* compound **2**, there was no significant effect of **DMT‐dT** (Figure S81), but for the other compounds there was an increase in chloride transport activity in the presence of **DMT‐dT** (Figure S72–S82). The fact that **DMT‐dT** does not have the same effect on the Clˉ transport ability of all transporters confirms that general membrane disruption by **DMT‐dT** is unlikely and that the increased transport rates are most likely the result of direct interactions between **DMT‐dT** and the anion or between **DMT‐dT** and the transporters. The small preference for **DMT‐dT** over other co‐transporters suggests that Watson‐Crick base pairing could be involved in the co‐transport mechanism, but other types of interactions cannot be fully excluded.

**Figure 5:**
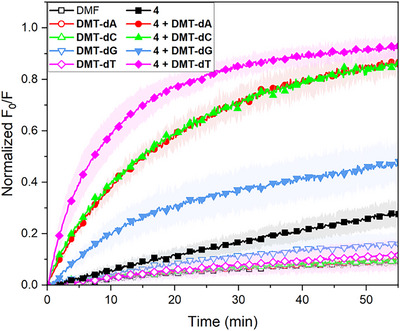
cAMP transport facilitated by a 3 mol% transporter **1** and 10 mol% of various co‐transporters (**DMT‐dT**, **DMT‐dA**, **DMT‐dC**, **DMT‐dG**). Experiments were performed with 200 nm POPC LUVs containing 0.8 mM SPBA, 225 mM NaNO_3_, 10 mM HEPES at pH 7.4 and suspended in a solution of 25 mM cAMP sodium salt, 225 mM NaNO_3_, 10 mM HEPES at pH 7.4. Data represents normalized fluorescence intensity, and is the average of minimum 3 independent repeats.

In conclusion, we have demonstrated the first example of direct transmembrane translocation of cAMP and AMP facilitated by neutral small molecules using a two‐component system. New liposome‐based assays based on ^31^P NMR or fluorescence quenching of SPBA provided strong evidence for the ability of these carriers to transport monophosphate nucleotides without disruption of membrane integrity. The most effective system combined a fluorinated mono‐squaramide as the anionophore, and a lipophilic thymine derivative as a co‐transporter. These results could open the door towards applications of nucleotide transport, such as antiviral drug delivery and artificial cells. Efforts to extend this strategy to the more negatively charged nucleotides ADP and ATP are currently underway.

## Supporting Information

The authors have cited additional references within the Supporting Information.^[^ ^57^, ^58^, ^59^, ^60^, ^61^, ^62^, ^63^ ^]^

## Author Contributions

Uththara M.C. Rathnaweera carried out chemical synthesis for transporters 4–6 and the SPBA dye, fluorescence studies, ^31^P NMR studies, and ^1^H NMR studies. Olivia Sam contributed to the fluorescence studies for AMP. Karolis Norvaisa carried out molecular modelling and inorganic phosphate transport studies. Sarah R. Marshall synthesized transporters 1–3. Randima D. De Silva Weerakonda Arachchige synthesized transporter 10. M. Chvojka provided SPBA dye for initial studies. Uththara M.C. Rathnaweera, Karolis Norvaisa, and Nathalie Busschaert wrote the manuscript. Nathalie Busschaert conceived and supervised the entire project. Hennie Valkenier and Karolis Norvaisax contributed to the experiment design and validation and all co‐authors edited the manuscript.

## Conflict of Interests

The authors declare no conflict of interest.

## Supporting information



Supporting Information

## Data Availability

The data that support the findings of this study are available in the Supporting Information of this article.
