# Supplementary material for: Transmembrane Transport of cAMP and AMP Using a Two Component Small Molecule Transport System
Source: Angew Chem Int Ed Engl. 2025 Dec 9;65(4):e24663. doi: 10.1002/anie.202524663 (PMC12828476; doi:10.1002/anie.202524663)
Supplement: Supplementary file 1 — Supporting Information [file ANIE-65-e24663-s001.pdf]

# Table of Contents

S1. General information ..................... S2
S2. Charges and p$K_a$ calculations for nucleotides ..................... S4
S3. Fluorescence quenching of SPBA by nucleotides ..................... S6
S4. Optimization of transporter concentrations ..................... S9
*S4.1. Preparation of large unilamellar vesicles* ..................... S9
*S4.2. Solubility experiment* ..................... S9
*S4.3. DLS experiment* ..................... S10
S5. Transport studies ..................... S12
*S5.1. Protocol of the cAMP or AMP transport assays and data processing* ..................... S12
*S5.2. Comparative dye leakage analysis of lucigenin and SPBA over time* ..................... S13
*S5.3. SPBA assay for cAMP transport: screening* ..................... S14
*S5.4. SPBA assay for cAMP transport: Hill plot for transporters* ..................... S19
*S5.5. SPBA assay for cAMP transport: Hill plot for co-transporters* ..................... S23
*S5.6. SPBA assay for AMP transport: screening* ..................... S27
*S5.7. SPBA assay for AMP transport: pH dependence* ..................... S30
S6. Evaluating potential factors leading to false positive results ..................... S31
*S6.1. Addition of nucleotide solution at different times* ..................... S31
*S6.3. Quantifying chloride contamination in nucleotide samples* ..................... S36
S7. Nucleotide transport studies using $^{31}$P NMR assay ..................... S36
*S7.1. General* ..................... S36
*S7.2. Visualization of intravesicular nucleotide by paramagnetic reagent ($MnSO_4$)* ..................... S37
*S7.3. Visualization of intravesicular nucleotide by NMR shift reagent $Eu(NO_3)_3$* ..................... S41
S.8. $^{1}$H NMR titrations ..................... S43
S9. MM calculations ..................... S48
S10. Evidence for co-transport mechanism ..................... S55
*S10.1. Calcein leakage assay* ..................... S55
*S10.2. DPPC assay* ..................... S58
*S10.3. Membrane fluidity measurements* ..................... S60
S10.3.1. Laurdan assay ..................... S60
S10.3.2. DPH assay ..................... S61
*S10.4. Effect of DMT-dT on the transport of other anions* ..................... S63
S10.4.1. Effect of DMT-dT on chloride transport ..................... S63
S10.4.2. Effect of DMT-dT on transporter deliverability ..................... S69
S10.4.3. Effect of DMT-dT on phosphate transport ..................... S71
*S10.5. Effect of other DMT nucleosides on the transport of cAMP* ..................... S73
S11. References ..................... S78

## S1. General information

All reagents, solvents and sodium salts of some nucleotides (AMP, ADP and ATP) were purchased from commercial sources and used without further purification (see **Table S1** for suppliers). The cAMP mono sodium salt was prepared by adding aliquots of cAMP solid into an aqueous NaOH solution (0.5 M) until pH = 7, followed by lyophilization using a Labconco FreeZone 2.5L Freeze Dryer to obtain a white powder. The cAMP-TBA salt was prepared by adding aliquots of TBA-OH (in 40% $H_2O$) to an aqueous cAMP solution until pH = 7, followed by lyophilization using a Labconco FreeZone 2.5L Freeze Dryer to obtain a white powder. The structures of all the transporters and co-transporters are shown in **Scheme S1**. Transporters **1-6** and **10** were synthesized as previously reported.[1, 2] Transporters **7**, **8**, **9**, and co-transporters **DMT-dT** and **Me-T** were purchased commercially and used as provided by the manufacturer (see **Table S1** for suppliers). The SPBA dye *(bis(3-sulfopropyl)-9,9'-biacridine bis(3-sulfopropyl)-9,9'-biacridine)*) was synthesized as previously reported.[3, 4] The structure of SPBA is shown in **Scheme S2**. POPC (1-palmitoyl-2-oleoyl-*sn*-glycero-3-phosphocholine) and DPPC (1,2-dipalmitoyl-*sn*-glycero-3-phosphocholine) lipids were purchased by Avanti Polar Lipids as a solution in chloroform (25 mg/mL) and were stored at -20 °C. Buffer solutions were prepared fresh every day from Ultra-Pure water collected from an ELGA Pure Lab ultrapure water system. Fluorescence measurements were performed on an Agilent Cary Eclipse fluorometer equipped with a multi-cell holder, stirring function and Peltier temperature controller. All fluorescence experiments were performed at 25°C in 3 mL glass cuvettes containing a stir bar for cuvettes with stirring set to maximum. $^{31}$P NMR experiments were performed on a Bruker ASCEND 600 MHz NMR at 298 K and were obtained proton decoupled.

**Table S1.** List of suppliers and the respective CAS numbers for the chemicals used in this manuscript.

| Compound | Supplier | CAS number |
|---|---|---|
| Adenosine 3',5'-Cyclic Monophosphate | TCI Chemicals | 60-92-4 |
| Adenosine 5'-monophosphate disodium salt | Sigma Aldrich | 4578-31-8 |
| Adenosine 5'-diphosphate, disodium salt hydrate, 98% | Thermo Scientific Chemicals | 16178-48-6 |
| Adenosine 5'-Triphosphate Disodium Salt Hydrate | Thermo Scientific Chemicals | 34369-07-8 |
| Transporter **7** | Sigma Aldrich | 102-07-8 |
| Transporter **8** | Sigma Aldrich | 102-08-9 |
| Transporter **9** | TCI Chemicals | 1960-88-9 |
| 5'-O-(4,4'-Dimethoxytrityl)thymidine (**DMT-dT**) | TCI Chemicals | 40615-39-2 |
| 1-Methylthymine (**Me-T**) | Sigma Aldrich | 4160-72-9 |

| | | |
|---|---|---|
| **DMT-dA** | Sigma Aldrich | 17331-22-5 |
| **DMT-dG** | Sigma Aldrich | 81144-43-6 |
| **DMT-dC** | Sigma Aldrich | 76512-82-8 |

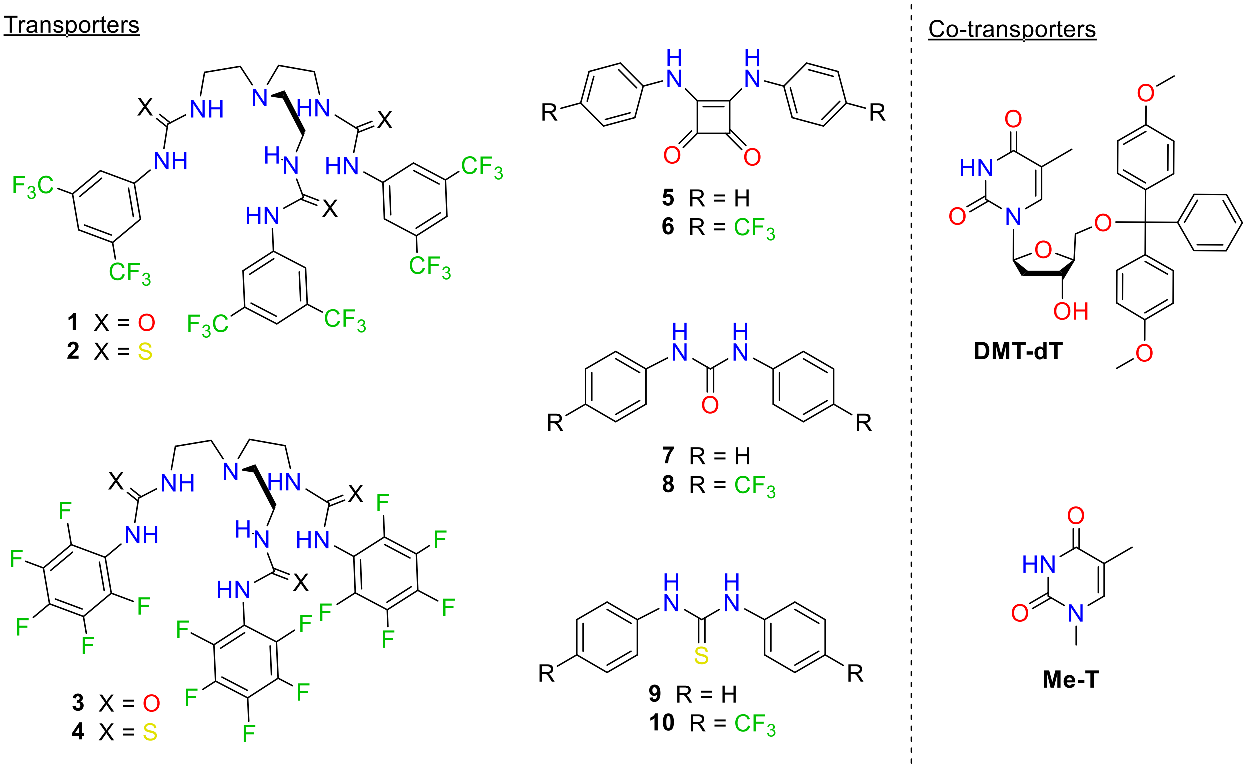


**Scheme S1.** Structures of the transporters and co-transporters used in this manuscript.

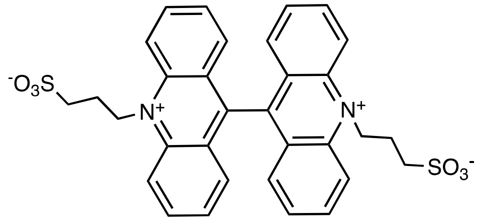

**Scheme S2.** Structures of the dye SPBA used in the transport experiments.

## S2. Charges and p$K_a$ calculations for nucleotides

The p$K_a$ values and charge at various pHs were calculated for the different adenine nucleotides (cAMP, AMP, ADP and ATP) using ChemAxon's online tool Chemicalize. The results are shown in **Figure S1-Figure S5** The figures also show the average charge at pH 7.4, which is the pH used for the transport experiments. The calculated p$K_a$ value for AMP (6.45) is in close agreement with experimental reports (6.5),[5] indicating that the calculations can give a good estimate of the various charged species present during the transport experiments.

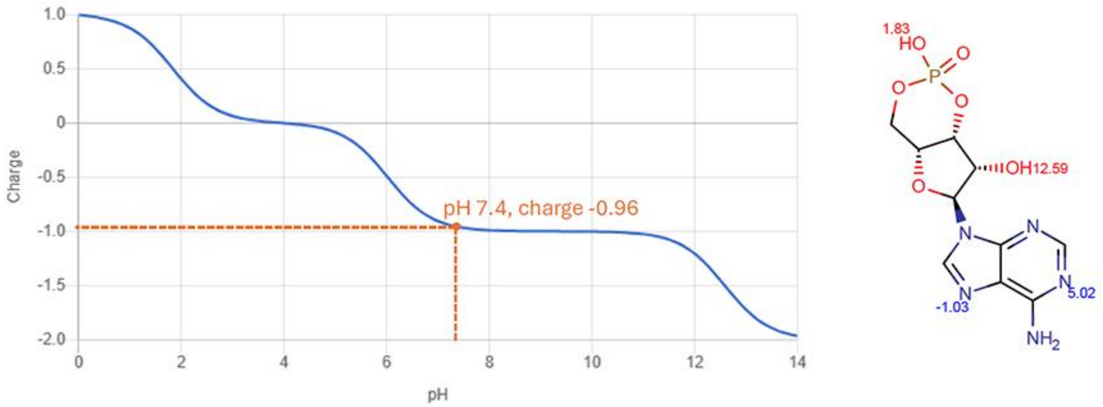


**Figure S1**. Charge vs. pH graph and p$K_a$ values for cAMP calculated using Chemicalize.

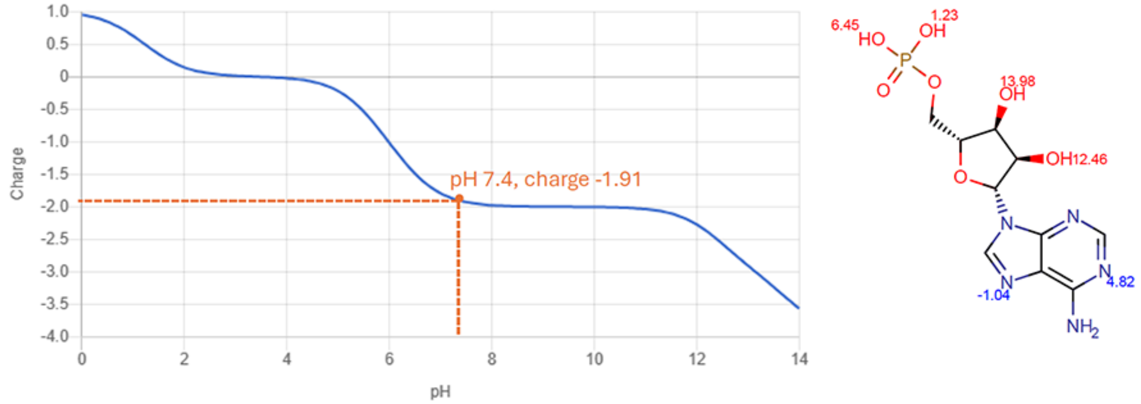


**Figure S2**. Charge vs. pH graph and p$K_a$ values for AMP calculated using Chemicalize.

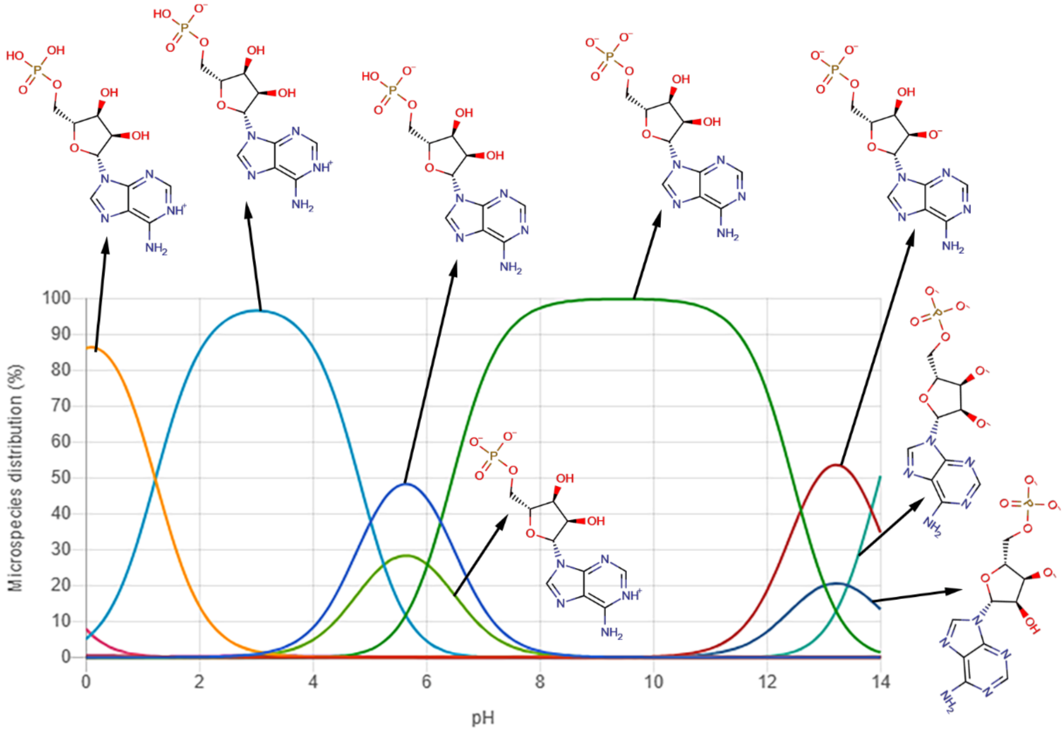


**Figure S3**. Speciation Curve for AMP at different pHs calculated using Chemicalize.

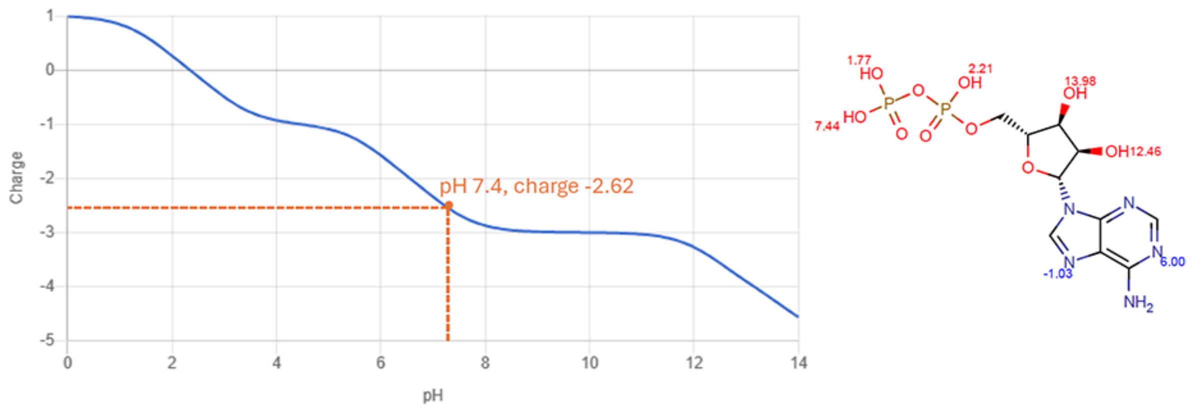


**Figure S4**. Charge vs. pH graph and p$K_a$ values for ADP calculated using Chemicalize.

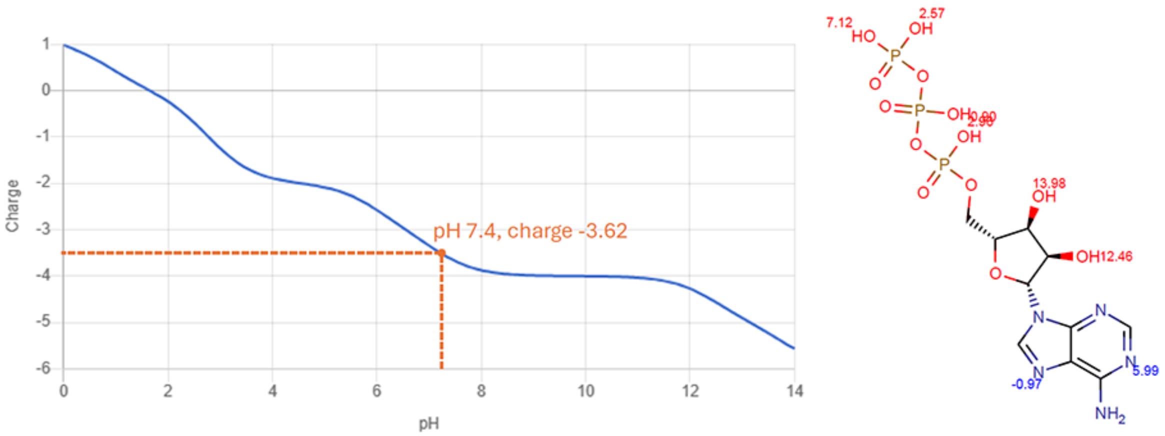


**Figure S5**. Charge vs. pH graph and p$K_a$ values for ATP calculated using Chemicalize.

## S3. Fluorescence quenching of SPBA by nucleotides

To determine if SPBA can be used to measure nucleotide transport, Stern-Volmer-type quenching of SPBA by various nucleotides was first determined. To a solution of 0.6 µM SPBA in nitrate buffer (225 mM $NaNO_3$, 10 mM HEPES, pH 7.4), was added aliquots of the sodium salt of the nucleotides at various concentrations. The fluorescence spectrum ($\lambda_{ex}$ = 435 nm, $\lambda_{em}$ = 505 nm) was measured before and after the addition of sodium salt of nucleotides. The spectra were normalized by dividing the fluorescence intensity at any wavelength by the fluorescence intensity at 505 nm before the addition of nucleotide. For the Stern-Volmer constant, the $F_0/F$ value at 505 nm was calculated and plotted against the nucleotide concentration, whereby $F_0$ is the fluorescence intensity at 505 nm before the addition of nucleotide and $F$ is the intensity after addition. The Stern-Volmer constant, $K_{SV}$, is given by the slope of the linear fit (the intercept is fixed at a value of 1). The experiments were performed in duplicate and the results are shown in **Figure S6-Figure S9.**

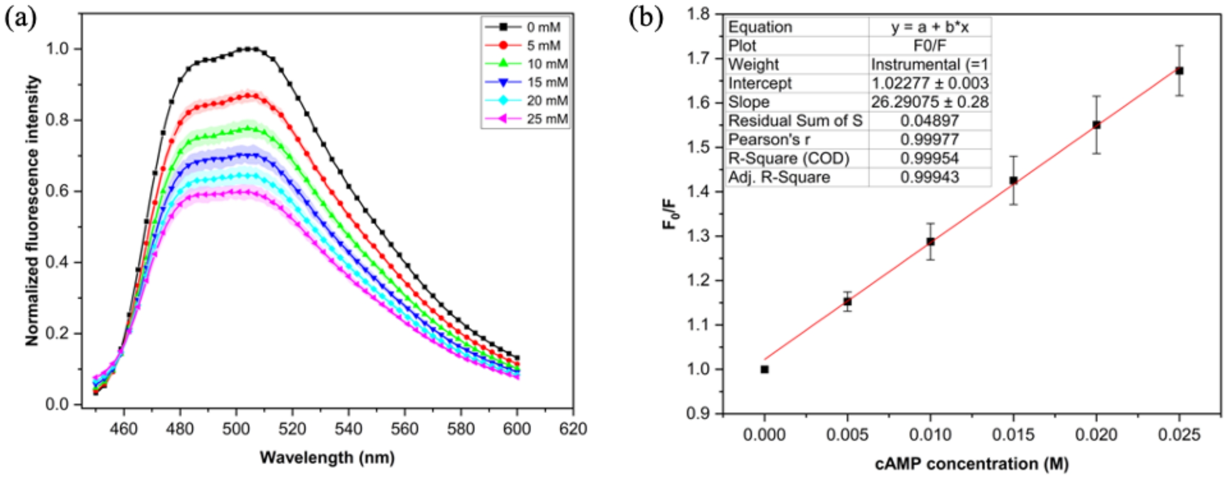


**Figure S6**. (a) Normalized fluorescence spectra of SPBA in the presence of various concentrations of cAMP sodium salt. The excitation wavelength was 435 nm. (b) Stern-Volmer plot of the quenching of SPBA by various concentrations cAMP sodium salt.

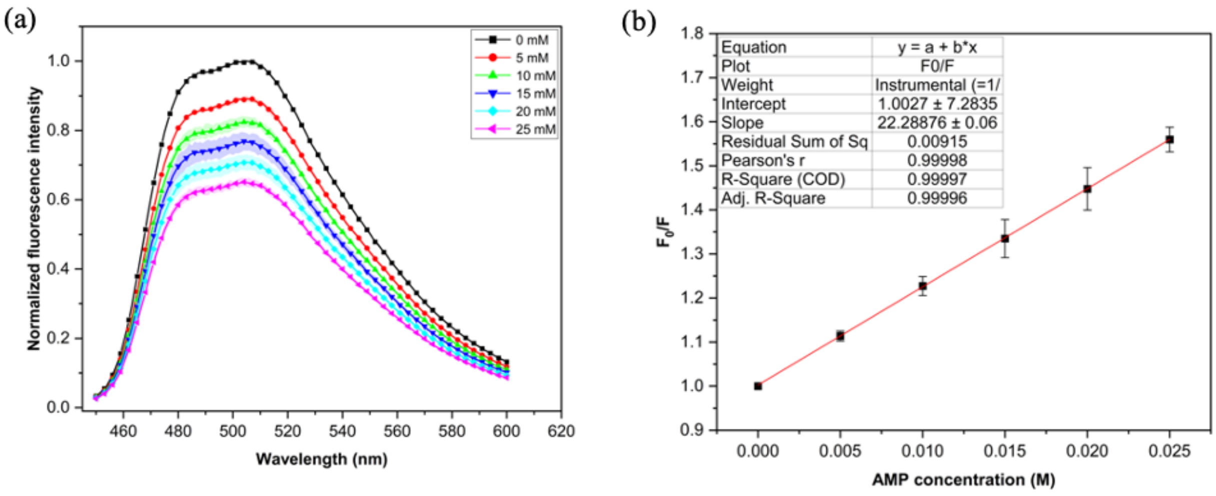


**Figure S7**. (a) Normalized fluorescence spectra of SPBA in the presence of various concentrations of AMP sodium salt. The excitation wavelength was 435 nm. (b) Stern-Volmer plot of the quenching of SPBA by various concentrations AMP sodium salt.

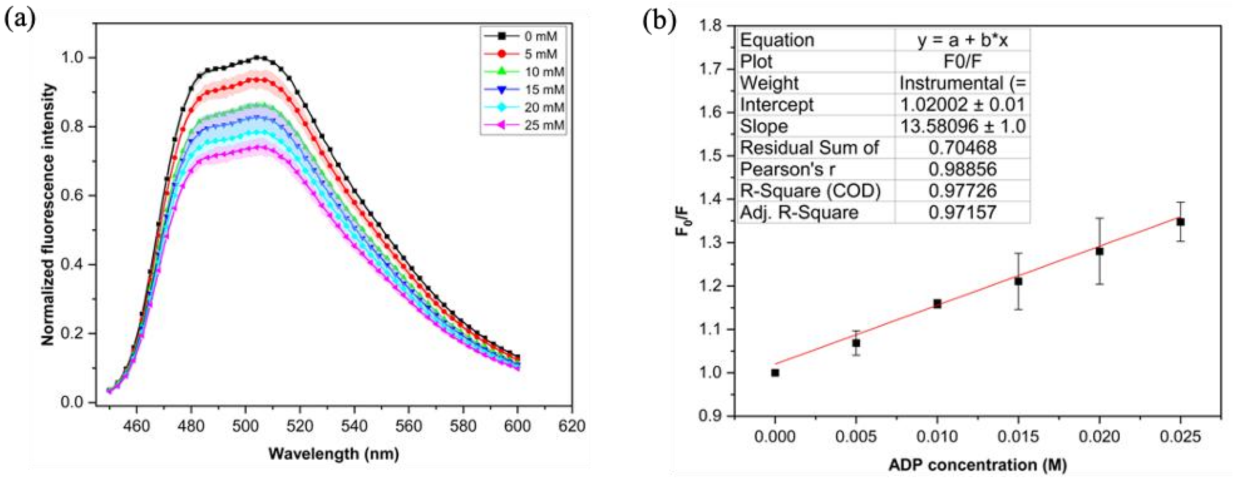


**Figure S8**. (a) Normalized fluorescence spectra of SPBA in the presence of various concentrations of ADP sodium salt. The excitation wavelength was 435 nm. (b) Stern-Volmer plot of the quenching of SPBA by various concentrations ADP sodium salt.

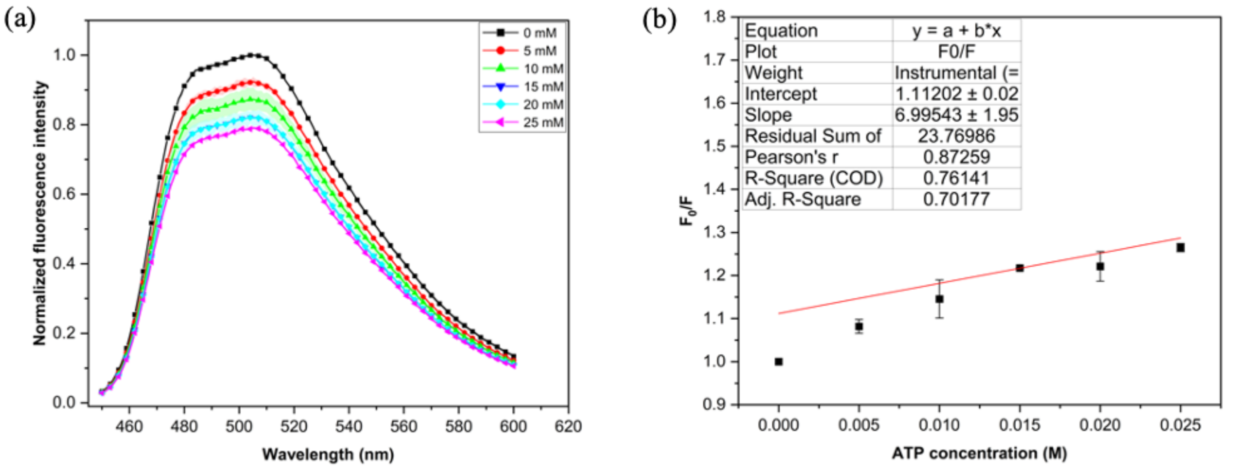


**Figure S9**. (a) Normalized fluorescence spectra of SPBA in the presence of various concentrations of ATP sodium salt. The excitation wavelength was 435 nm. (b) Stern-Volmer plot of the quenching of SPBA by various concentrations ATP sodium salt.

# S4. Optimization of transporter concentrations

### *S4.1. Preparation of large unilamellar vesicles*

A thin film of POPC lipid was formed by evaporating a chloroform solution of lipid under reduced pressure, followed by drying under high vacuum for at least for 8 hours. The lipid film was hydrated by vortexing with the prepared buffer solution (225 mM $NaNO_3$, 10 mM HEPES at pH 7.4). The lipid suspension was then subjected to eleven freeze-thaw cycles alternating between submersion in liquid nitrogen followed by thawing in a mildly warm water bath (below 34 °C). The lipid suspension was allowed to rest at room temperature for 30 min and was subsequently extruded 29 times through a 200 nm polycarbonate membrane using the Avanti mini extruder set (Avanti Polar Lipids, Inc.). The obtained concentrated stock liposome solution was diluted in external buffer (225 mM $NaNO_3$, 10 mM HEPES at pH 7.4) to afford a final total lipid concentration of 0.5 mM.

### *S4.2. Solubility experiment*

Prior to conducting transport assays, the solubility of each transporter in the liposome solution was determined. Large unilamellar vesicles were prepared as described in **section S4.1**. to obtain 200 nm POPC LUVs at a final lipid concentration of 0.5 mM. 3 mL of this liposome solution was placed in glass cuvettes, and a 15 µL aliquot of each transporter (from a DMF stock) was added. This resulted in final transporter concentrations ranging from 1 mol% to 10 mol% relative to the lipid concentration, with and without 10 mol% **DMT-dT**. The mixture was stirred for 2 minutes. For most of the transporters, at concentrations in the range of 5 - 10 mol%, we observed the formation of particles in the solution shortly after adding the transporter solutions. In contrast, all solutions with concentrations below 5 mol% remained clear within seconds of addition. However, for compound **6** even a concentration of 4 mol% showed cloudiness and evidence of limited solubility (**Figure S10** and **Figure S11**). Therefore, we selected a transporter concentration of 3 mol% and co-transporter concentration 10 mol% for all the subsequent experiments.

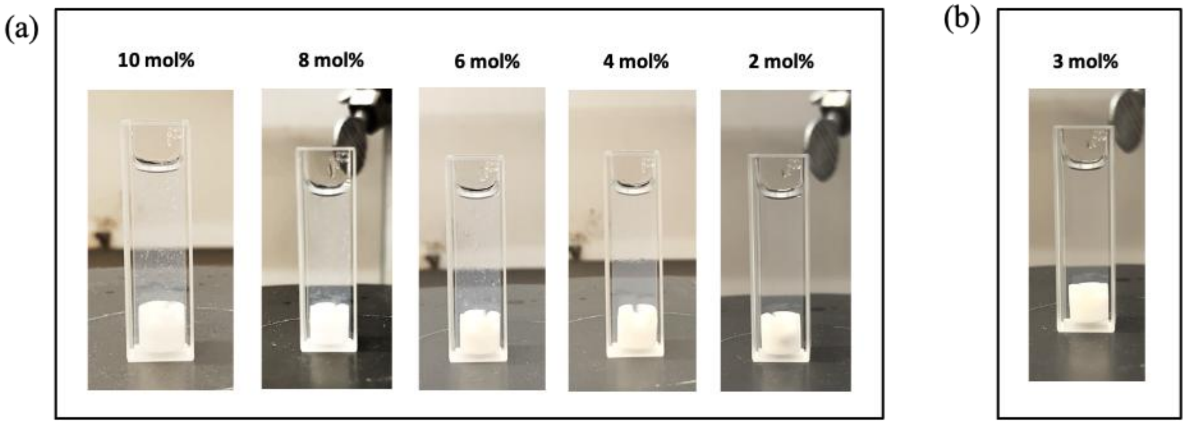


**Figure S10**. The images of the solubility experiment for transporter **6** (a) at different concentrations (10, 8, 6, 4 and 2 mol% to lipid) (b) at concentration 3 mol% taken after addition and stirring for 2 minutes.

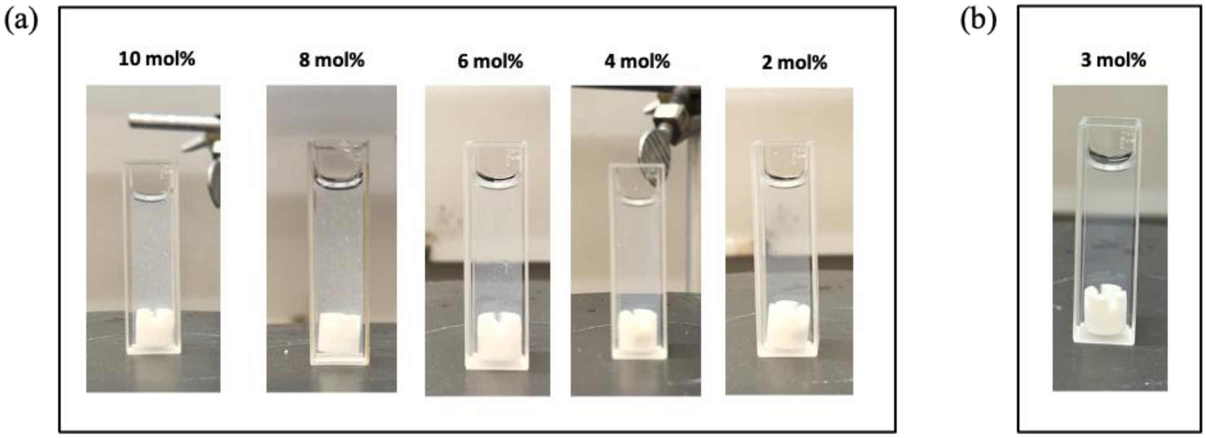


**Figure S11**. The images of the solubility experiment for transporter **6** (a) at different concentrations (10, 8, 6, 4 and 2 mol% to lipid) with **DMT-dT** (10 mol% to lipid) (b) at transporter concentration 3 mol% with **DMT-dT** 10 mol% taken after addition and stirring for 2 minutes.

## *S4.3. DLS experiment*

To further confirm that 3 mol% is a suitable concentration, we performed dynamic light scattering experiments. Large unilamellar vesicles were prepared as described in **section S4.1**. to obtain 200 nm POPC LUVs at a final lipid concentration of 0.5 mM. 3 mL of this liposome solution was placed in glass cuvettes, and a 15 µL aliquot of each transporter (from a DMF stock) was added to achieve final transporter concentrations of 3 mol% or 5 mol% relative to the lipid concentration, with and without 10 mol% **DMT-dT**. The dynamic light scattering (DLS) spectra were measured for each sample using a NanoBrook Omni (Brookhaven Instruments) particle size

analyzer. In agreement with the solubility test results, in addition to the expected 200 nm LUV peak, additional peaks were observed in some of the samples containing 5 mol% transporter - indicating particle precipitation (**Figure S12**). On the other hand, samples with 3 mol% transporter did not show any extra peak in the DLS spectra (**Figure S13**). This further confirmed that 3 mol% is a suitable concentration to test the nucleotide transport ability of the compounds.

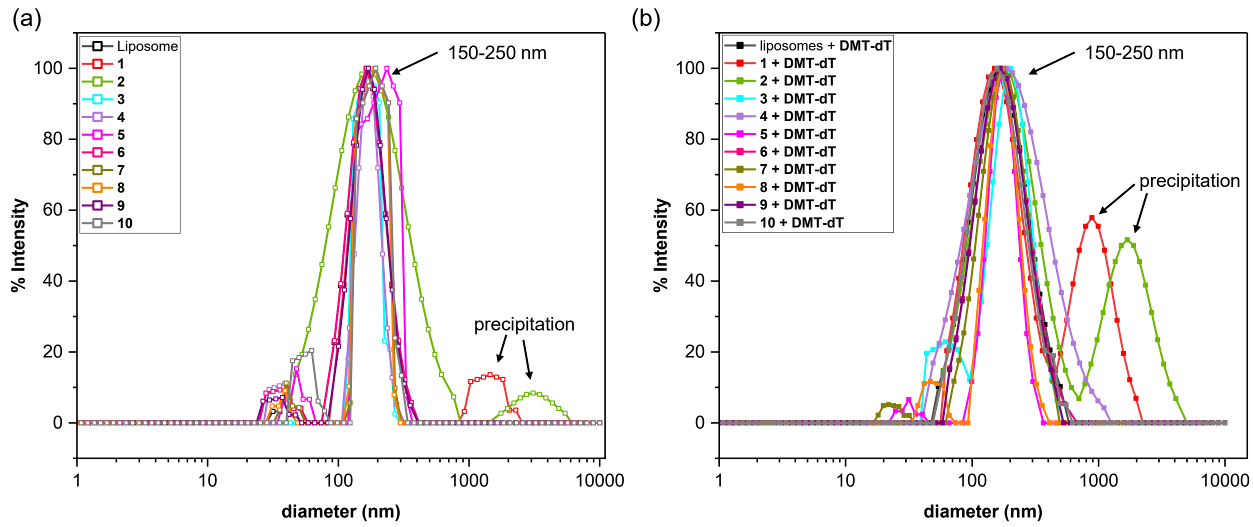


**Figure S12.** DLS spectra of 200 nm LUVs containing transporter at 5 mol% with respect to lipid with and without 10 mol% **DMT-dT** to lipid. (a) without **DMT-dT**. (b) with **DMT-dT**.

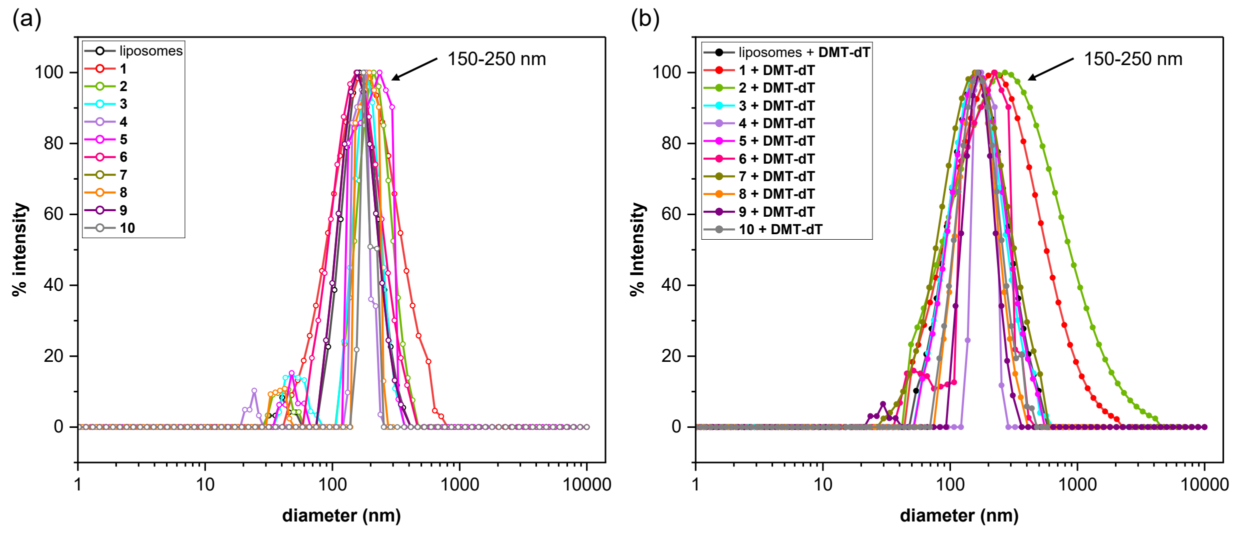


**Figure S13.** DLS spectra of 200 nm LUVs containing transporter at 3 mol% with respect to lipid with and without 10 mol% **DMT-T** to lipid. (a) without **DMT-T**. (b) with **DMT-T**.

# S5. Transport studies

## *S5.1. Protocol of the cAMP or AMP transport assays and data processing*

A thin film of POPC lipid was formed by evaporating a chloroform solution of lipid under reduced pressure, followed by drying under high vacuum for at least for 8 hours. The lipid film was hydrated by vortexing with a $NaNO_3$ solution (0.8 mM SPBA (or 1 mM lucigenin) in 225 mM $NaNO_3$, 10 mM HEPES at pH 7.4). The lipid suspension was then subjected to eleven freeze-thaw cycles alternating between submersion in liquid nitrogen followed by thawing in a mildly warm water bath (below 34 °C). The lipid suspension was allowed to rest at room temperature for 30 min and was subsequently extruded 29 times through a 200 nm polycarbonate membrane using the Avanti mini extruder set (Avanti Polar Lipids, Inc.). Unencapsulated dye was removed by size exclusion chromatography on a Sephadex G-25 column, eluted with 225 mM $NaNO_3$, 10 mM HEPES, pH 7.4. The final lipid concentration per sample was 0.5 mM.

The dye-loaded liposomes (0.5 mM lipid) were transferred into a 3 mL glass cuvette and placed in the sample compartment of an Agilent Cary Eclipse fluorescence spectrometer equipped with a magnetic stirrer, and a temperature controller. Stirring was initiated at maximum speed and maintained throughout the experiment. Two minutes before starting the kinetic run, 75 µL of a sodium salt of nucleotide stock solution (1 M in 225 mM $NaNO_3$ and 10 mM HEPES, pH 7.4)) was added to reach a final concentration of 25 mM. At $t$ = 0 min, the kinetic run began, at $t$ = 1 min, 15 µL of the transporter solution in DMF was added to initiate the influx of nucleotide anions and the efflux of $NO_3^-$ anions. The fluorescence intensity (lucigenin; $\lambda_{ex}$ = 430 nm, $\lambda_{em}$ = 505 nm and SPBA; $\lambda_{ex}$ = 435 nm, $\lambda_{em}$ = 505 nm) was recorded for 60 minutes. At time t = 60 min, detergent (75 µL of 10% Triton X-100) was added to fully lyse the membrane and estimate the quality of the liposomes. Transporter concentrations are given as mol% with respect to POPC lipid concentration.

The time scale of the crude kinetic run was corrected to ensure that nucleotide transport starts at $t$ = 0 (because the transporter was added after 1 min, this indicates shifting the time scale with 1 min). The corrected kinetic run was subsequently converted to '*Normalized $F_0/F$*', using the following equation (where $F_0$ is the fluorescence intensity at time $t$ = 0 min (1 min after the addition of transporter), $F$ is the fluorescence intensity at any time and $F_{final}$ is the fluorescence intensity after adding Triton X-100.

$$Normalized\ \frac{F_0}{F} = \frac{\frac{F_0}{F} - \frac{F_0}{F_0}}{\frac{F_0}{F}_{final} - \frac{F_0}{F_0}} = \frac{\frac{F_0}{F} - 1}{\frac{F_0}{F}_{final} - 1}$$

The calculation is valid because both cAMP and AMP showed a linear correlation between SPBA quenching (*$F_0/F$*) and nucleotide concentration. By taking the value upon addition of Triton X-100 ($F_{final}$) as a reference point, the data is normalized and the error between experiments is reduced. Subtracting 1 in the formula ensures that values start at 0 and reach a maximum value of 1. In general, the '*Normalized $F_0/F$*' data of 3-4 independent experiments conducted over at least 3 different sets of liposomes were averaged and the standard deviations were calculated.

### *S5.2. Comparative dye leakage analysis of lucigenin and SPBA over time*

To decide whether lucigenin or SPBA is the best dye to monitor nucleotide transport over long time scale, we compared the background leakage of the dye out of the liposomes. Liposomes were prepared as described in **section S5.1.**, using cAMP as the external anion and the addition of DMF instead of transporter. The results are shown in **Figure S14** and show more leakage of lucigenin compared to SPBA, as also previously observed for chloride transport.[6] We therefore decided to perform all nucleotide transport experiments with SPBA, because this has less problems with the dye leaking out of the liposomes. Initial screening tests using lucigenin also found many false positives for cAMP transport, and lucigenin was therefore abandoned.

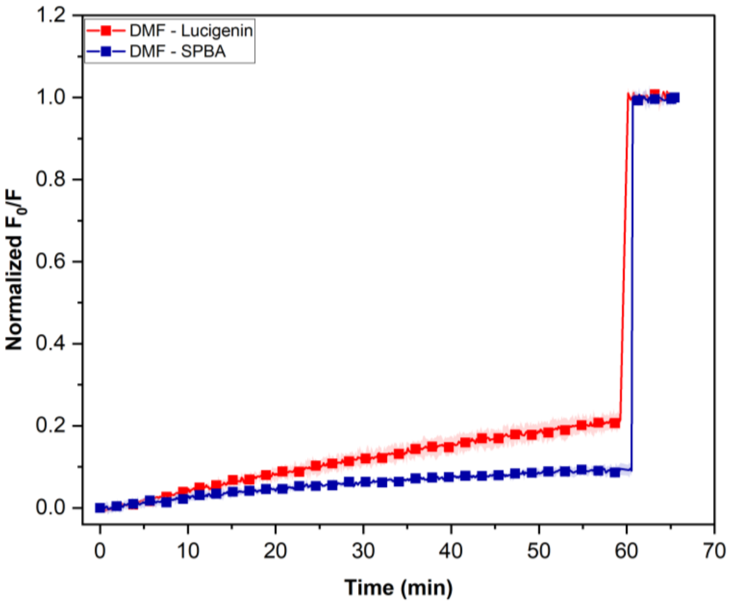


**Figure S14**. A comparative analysis of dye leakage between lucigenin and SPBA was conducted over 60 minutes. Experiment was performed as described in section S5.1. and is the average of a minimum of 2 repeats (shaded areas represent standard deviations.

### *S5.3. SPBA assay for cAMP transport: screening*

The initial screening for cAMP transport was conducted using 3 mol% transporter with or without 10 mol% co-transporter. The experiments were performed using the method described in **section S5.1**. and the results are shown in **Figure S15-Figure S24**. When a co-transporter was tested, a stock solution in DMF containing both transporter and co-transporter was used.

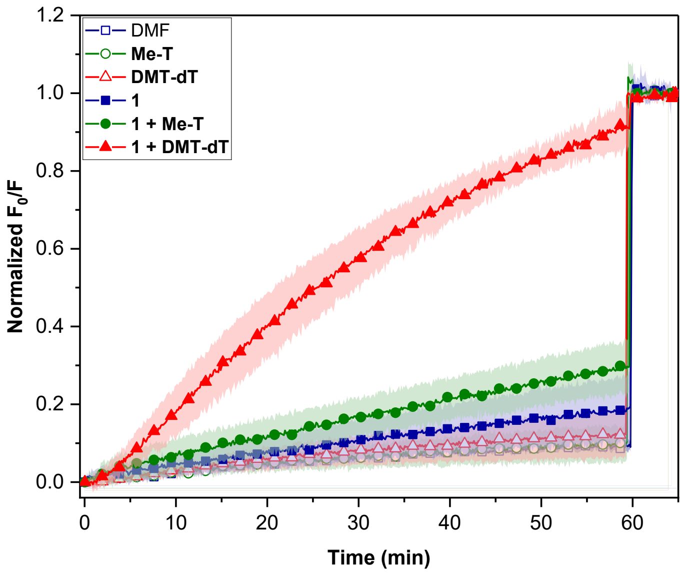


**Figure S15.** cAMP transport across 200 nm POPC LUVs mediated by transporter **1** (3 mol% to lipid) along with the co-transporters **DMT-dT** or **Me-T** (10 mol% to lipid). The experiment was performed as described in **section S5.1.** and is the average of minimum of 3 repeats (shaded areas represent standard deviations).

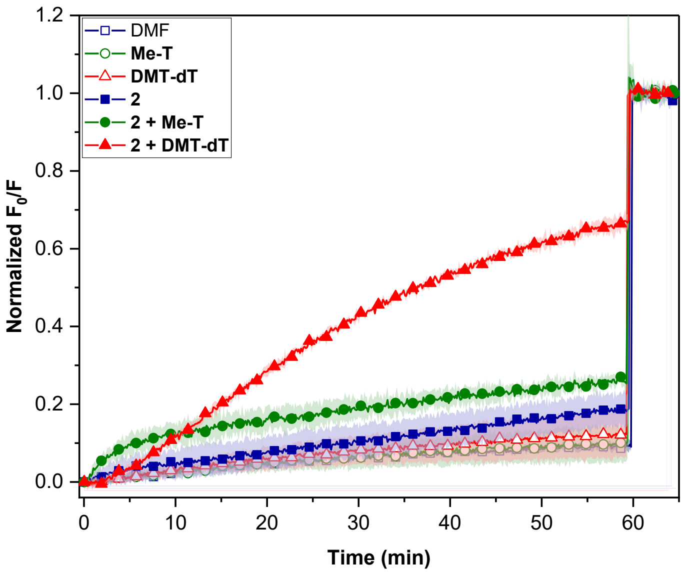


**Figure S16.** cAMP transport across 200 nm POPC LUVs mediated by transporter **2** (3 mol% to lipid) along with the co-transporters **DMT-dT** or **Me-T** (10 mol% to lipid). The experiment was performed as described in **section S5.1.** and is the average of minimum of 3 repeats (shaded areas represent standard deviations).

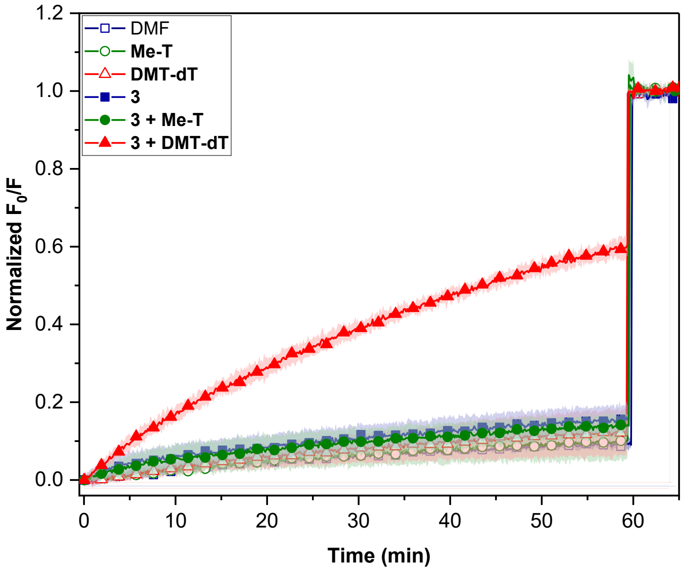


**Figure S17.** cAMP transport across 200 nm POPC LUVs mediated by transporter **3** (3 mol% to lipid) along with the co-transporters **DMT-dT** or **Me-T** (10 mol% to lipid). The experiment was performed as described in **section S5.1.** and is the average of minimum of 3 repeats (shaded areas represent standard deviations).

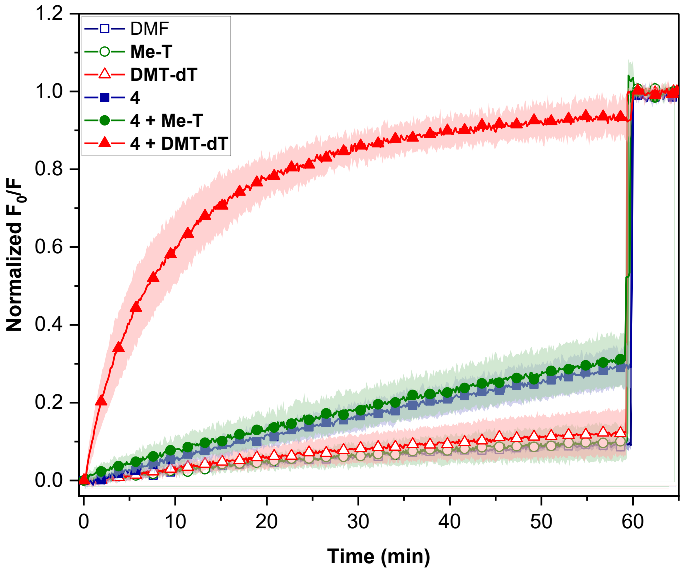


**Figure S18.** cAMP transport across 200 nm POPC LUVs mediated by transporter **4** (3 mol% to lipid) along with the co-transporters **DMT-dT** or **Me-T** (10 mol% to lipid). The experiment was performed as described in **section S5.1.** and is the average of minimum of 3 repeats (shaded areas represent standard deviations).

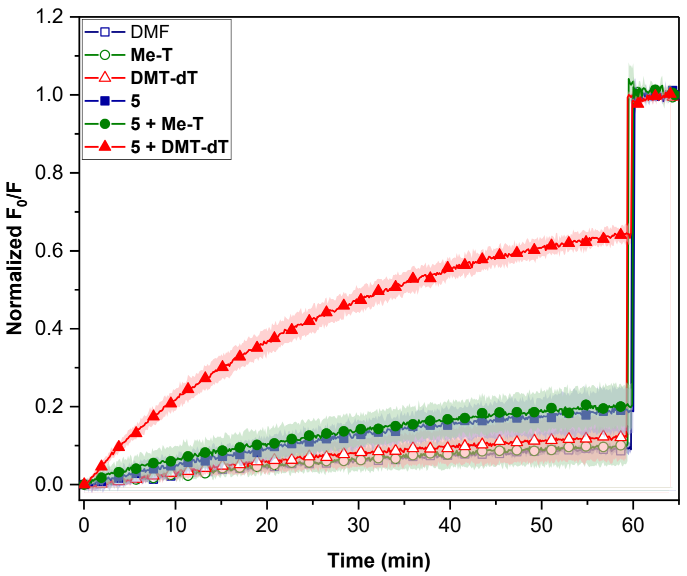


**Figure S19.** cAMP transport across 200 nm POPC LUVs mediated by transporter **5** (3 mol% to lipid) along with the co-transporters **DMT-dT** or **Me-T** (10 mol% to lipid). The experiment was performed as described in **section S5.1.** and is the average of minimum of 3 repeats (shaded areas represent standard deviations).

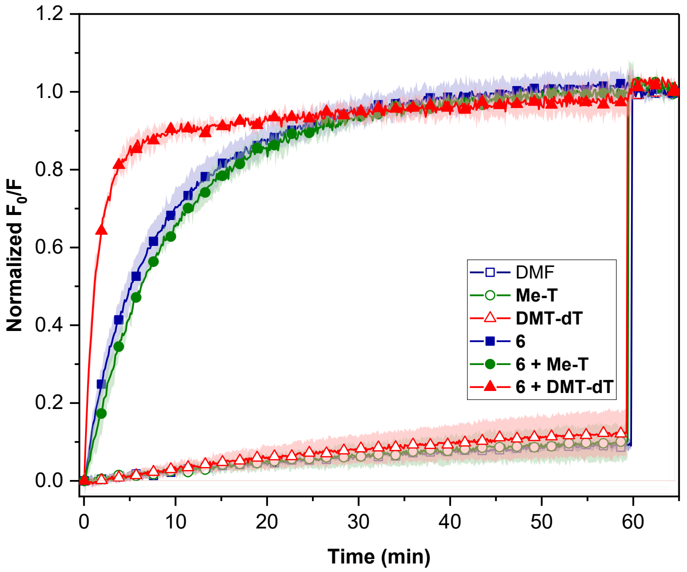


**Figure S20.** cAMP transport across 200 nm POPC LUVs mediated by transporter **6** (3 mol% to lipid) along with the co-transporters **DMT-dT** or **Me-T** (10 mol% to lipid). The experiment was performed as described in **section S5.1.** and is the average of minimum of 3 repeats (shaded areas represent standard deviations).

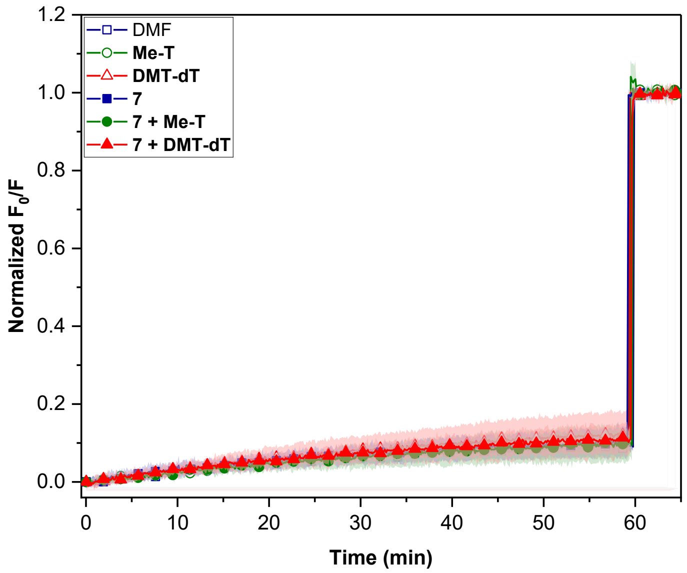


**Figure S21.** cAMP transport across 200 nm POPC LUVs mediated by transporter **7** (3 mol% to lipid) along with the co-transporters **DMT-dT** or **Me-T** (10 mol% to lipid). The experiment was performed as described in **section S5.1.** and is the average of minimum of 3 repeats (shaded areas represent standard deviations).

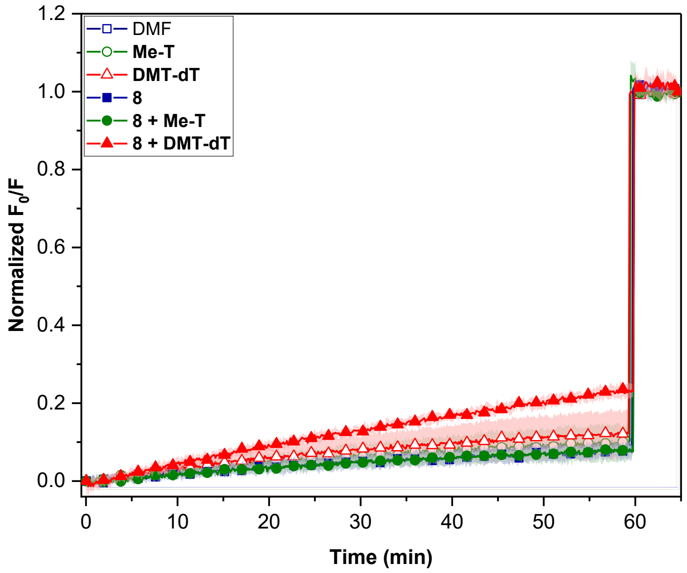


**Figure S22.** cAMP transport across 200 nm POPC LUVs mediated by transporter **8** (3 mol% to lipid) along with the co-transporters **DMT-dT** or **Me-T** (10 mol% to lipid). The experiment was performed as described in **section S5.1.** and is the average of minimum of 3 repeats (shaded areas represent standard deviations).

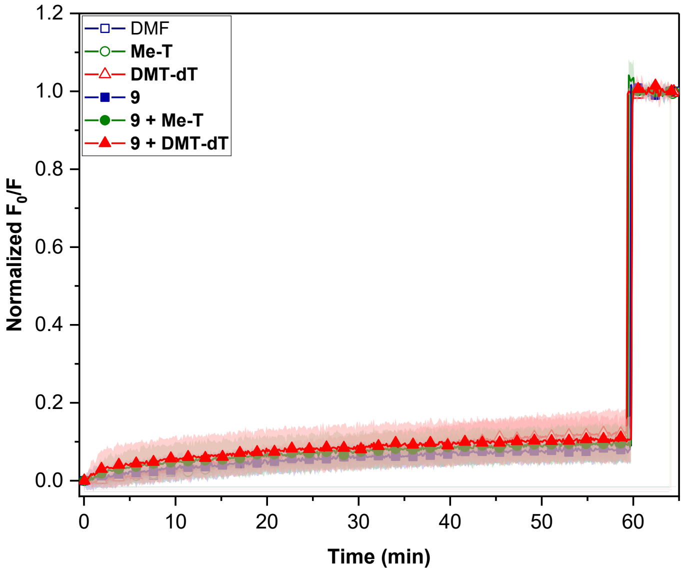


**Figure S23.** cAMP transport across 200 nm POPC LUVs mediated by transporter **9** (3 mol% to lipid) along with the co-transporters **DMT-dT** or **Me-T** (10 mol% to lipid). The experiment was performed as described in **section S5.1.** and is the average of minimum of 3 repeats (shaded areas represent standard deviations).

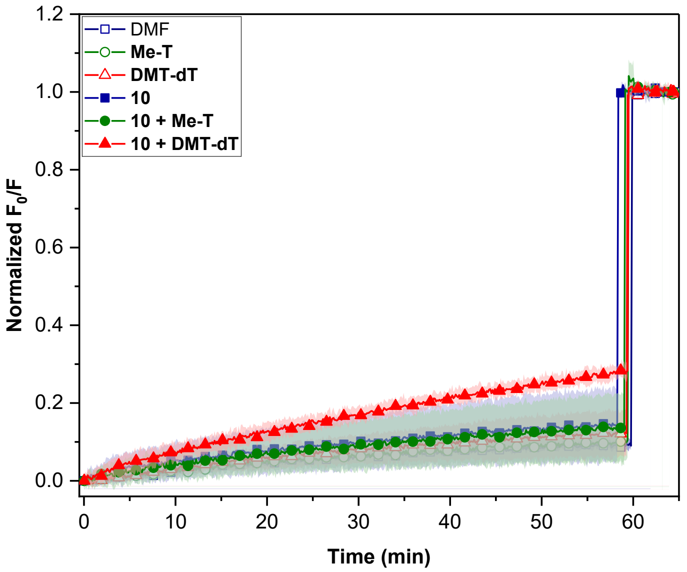


**Figure S24.** cAMP transport across 200 nm POPC LUVs mediated by transporter **10** (3 mol% to lipid) along with the co-transporters **DMT-dT** or **Me-T** (10 mol% to lipid). The experiment was performed as described in **section S5.1.** and is the average of minimum of 3 repeats (shaded areas represent standard deviations).

## *S5.4. SPBA assay for cAMP transport: Hill plot for transporters*

The cAMP transport assay was performed as described in **section S5.1.** for various concentrations of transporters **1-6** while maintaining constant **DMT-dT** concentration (10 mol% with respect to lipid). For transporter **6**, experiments were performed both in the presence and in the absence of **DMT-dT**. The *normalized* $F_0/F$ value 50 min after the addition of transporters was plotted as a function of the transporter concentration (mol %). Data points were fitted to the Hill 1 equation using Origin 2024b (10.15):

$$y = START + (END - START)\frac{x^n}{k^n + x^n}$$

where $y$ is the *normalized* $F_0/F$ value 50 min after the addition of transporter and $x$ is the transporter concentration (mol% with respect to lipid). *START, END*, $k$ and $n$ are the parameters to be fitted. *START* and *END* are the minimum and maximum values for y, $n$ is the Hill coefficient and $k$ is the transporter concentration at the inflection point of the graph and corresponds to the $EC_{50,50\,min}$ values (defined as the transporter concentration (mol % transporter to lipid) needed to obtain half maximum cAMP influx after 50 min). In theory START should be the background drift observed for 0 mol% transporter (**DMT-dT** addition only, or DMF only) and END should be 1. In most cases the values for START were fixed to 0.0974 (the observed value for the addition of **DMT-dT** only) or 0.09339 (the observed value for the addition of DMF only) and the END value was fixed to 1 (theoretical maximum which is indeed observed for compound **6**). The results are shown in **Figure S25**-**Figure S31**, and are summarized in **Table S2**.

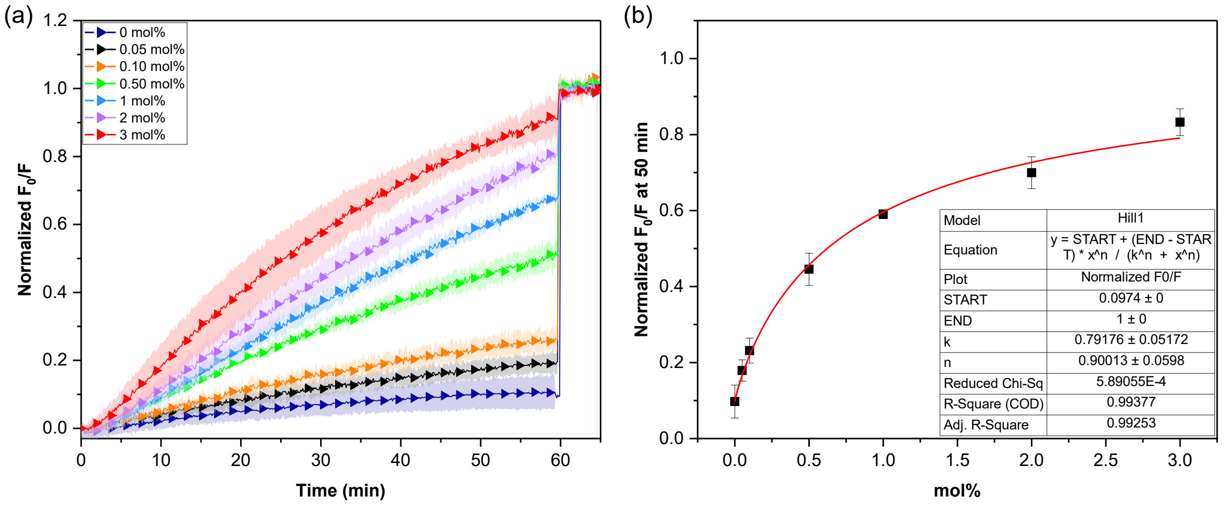


**Figure S25.** (a) cAMP influx mediated by transporter **1** from 200 nm unilamellar POPC vesicles loaded with 0.8 mM SPBA, 225 mM $NaNO_3$ and 10 mM HEPES at pH 7.4. Concentrations used: various mol% transporter and 10 mol% **DMT-dT** with respect to lipid. The experiments were performed as described in **section S5.1.** and is the average of minimum of 3 repeats. (shaded areas represent standard deviations). (b) Hill curves plotted from the cAMP influx values at 50 min.

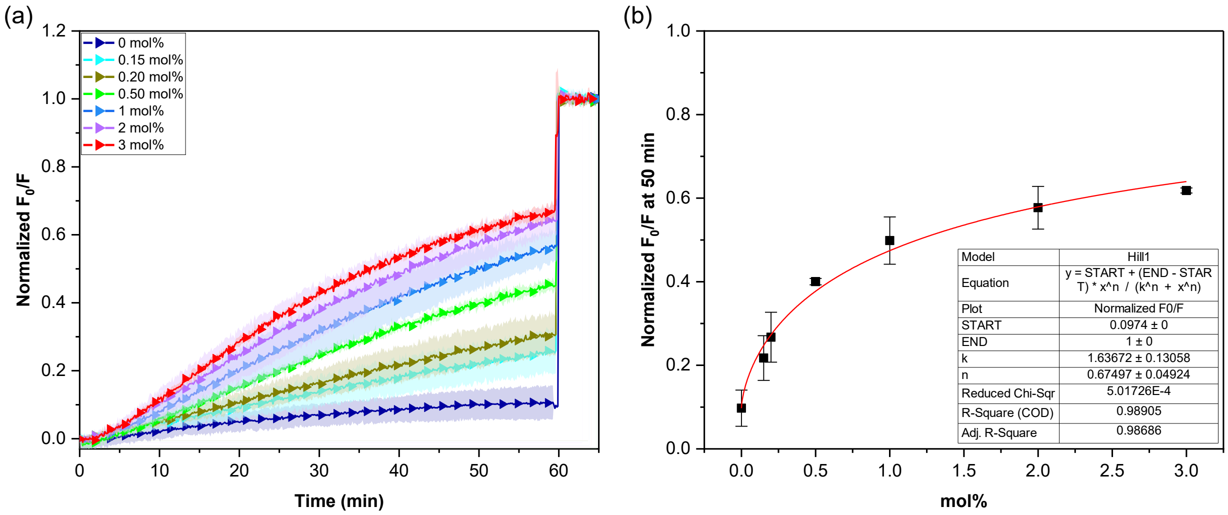


**Figure S26**. (a) cAMP influx mediated by transporter **2** from 200 nm unilamellar POPC vesicles loaded with 0.8 mM SPBA, 225 mM $NaNO_3$ and 10 mM HEPES at pH 7.4. Concentrations used: various mol% transporter and 10 mol% **DMT-T** with respect to lipid. The experiments were performed as described in **section S5.1.** and is the average of minimum of 3 repeats. (shaded areas represent standard deviations). (b) Hill curves plotted from the cAMP influx values at 50 min.

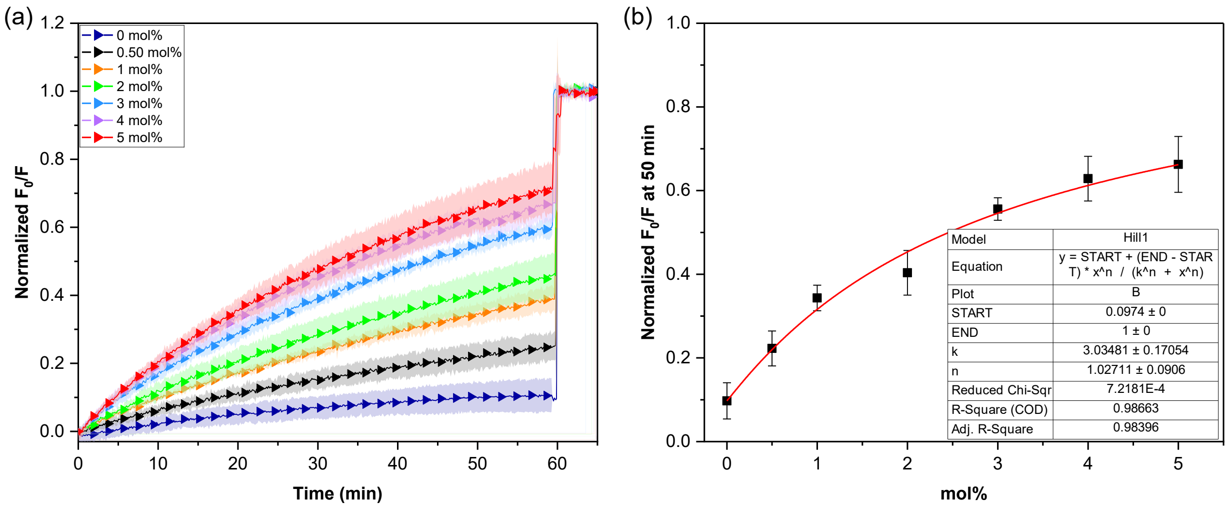


**Figure S27**. (a) cAMP influx mediated by transporter **3** from 200 nm unilamellar POPC vesicles loaded with 0.8 mM SPBA, 225 mM $NaNO_3$ and 10 mM HEPES at pH 7.4. Concentrations used: various mol% transporter and 10 mol% **DMT-dT** with respect to lipid. The experiments were performed as described in **section S5.1.** and is the average of minimum of 3 repeats. (shaded areas represent standard deviations). (b) Hill curves plotted from the cAMP influx values at 50 min.

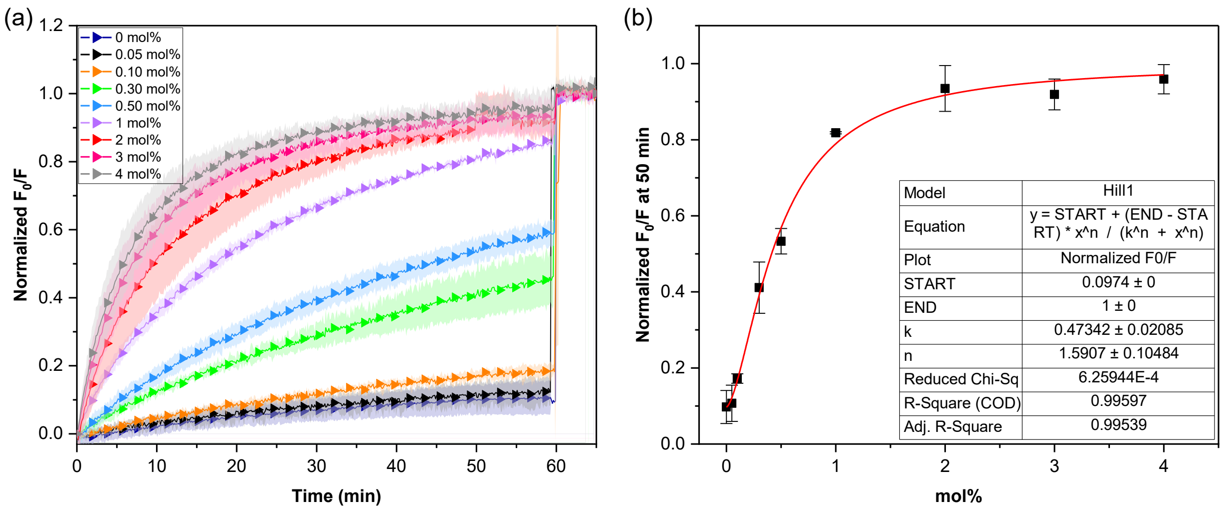


**Figure S28.** (a) cAMP influx mediated by transporter **4** from 200 nm unilamellar POPC vesicles loaded with 0.8 mM SPBA, 225 mM $NaNO_3$ and 10 mM HEPES at pH 7.4. Concentrations used: various mol% transporter and 10 mol% **DMT-dT** with respect to lipid. The experiments were performed as described in **section S5.1.** and is the average of minimum of 3 repeats. (shaded areas represent standard deviations). (b) Hill curves plotted from the cAMP influx values at 50 min.

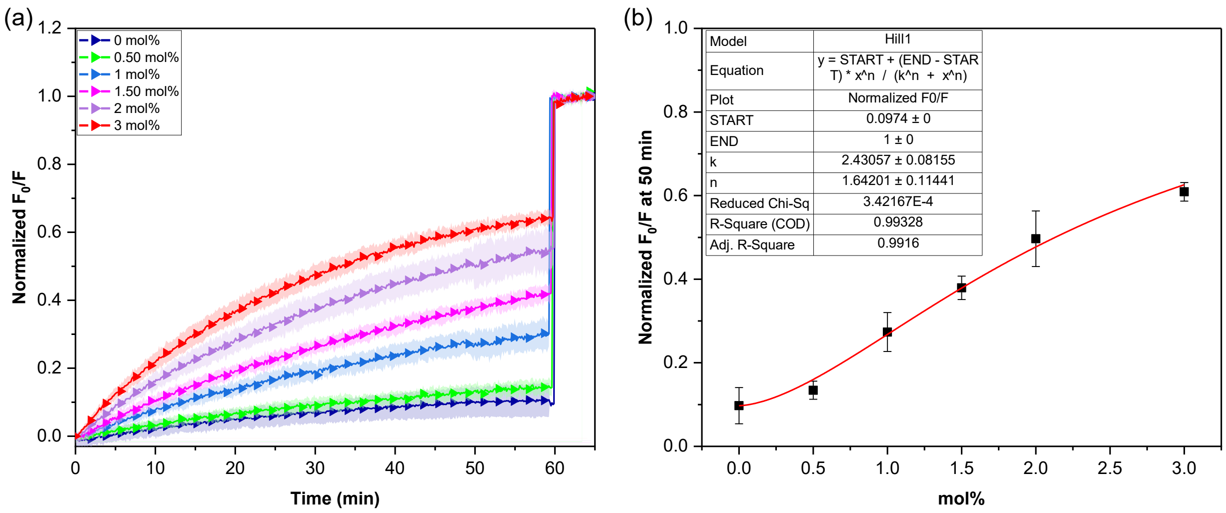


**Figure S29.** (a) cAMP influx mediated by transporter **5** from 200 nm unilamellar POPC vesicles loaded with 0.8 mM SPBA, 225 mM $NaNO_3$ and 10 mM HEPES at pH 7.4. Concentrations used: various mol% transporter and 10 mol% **DMT-dT** with respect to lipid. The experiments were performed as described in **section S5.1.** and is the average of minimum of 3 repeats. (shaded areas represent standard deviations). (b) Hill curves plotted from the cAMP influx values at 50 min.

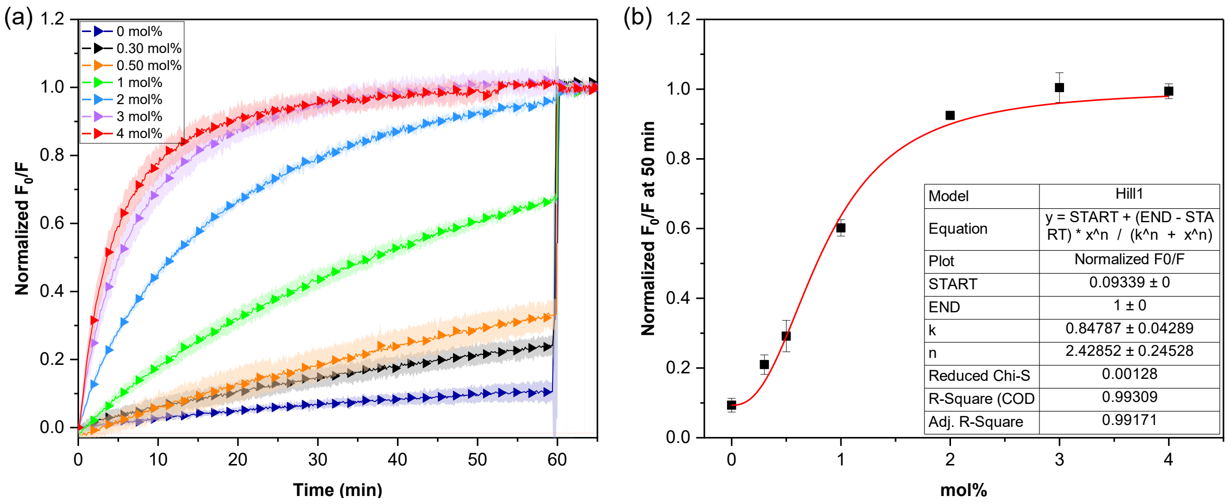


**Figure S30**. (a) cAMP influx mediated by transporter **6** from 200 nm unilamellar POPC vesicles loaded with 0.8 mM SPBA, 225 mM $NaNO_3$ and 10 mM HEPES at pH 7.4. Concentrations used: various mol% transporter to lipid. The experiments were performed as described in **section S5.1**. and is the average of minimum of 3 repeats. (shaded areas represent standard deviations). (b) Hill curves plotted from the cAMP influx values at 50 min.

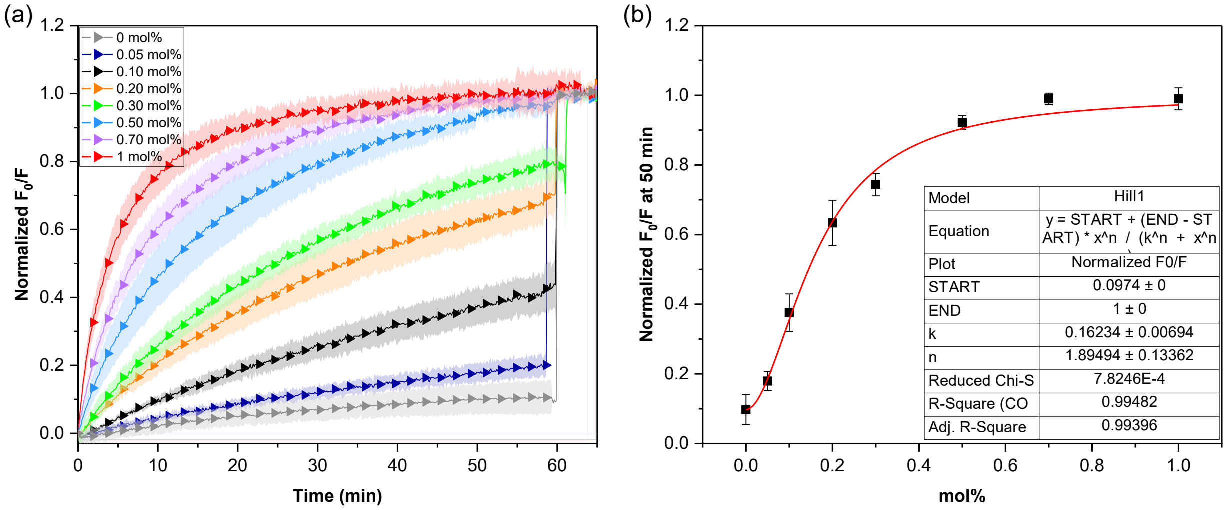


**Figure S31**. (a) cAMP influx mediated by transporter **6 + DMT-dT** from 200 nm unilamellar POPC vesicles loaded with 0.8 mM SPBA, 225 mM $NaNO_3$ and 10 mM HEPES at pH 7.4. Concentrations used: various mol% transporter and 10 mol% **DMT-dT** with respect to lipid. The experiments were performed as described in **section S5.1.** and is the average of minimum of 3 repeats. (shaded areas represent standard deviations). (b) Hill curves plotted from the cAMP influx values at 50 min.

**Table S2.** Summary of the cAMP anion transport (*$EC_{50}$* and *n*) properties of transporters **1-6** by varying either the transporter concentration or **DMT-dT** concentration

| Transporter | Transporter concentration varied[a] | | DMT-dT concentration varied[b] | |
|---|---|---|---|---|
| | ***$EC_{50}$*** **(mol%)[c]** | **Hill coefficient (*n*)** | ***$EC_{50}$*** **(mol%)[c]** | **Hill coefficient (*n*)** |
| One component system (transporter only) | | | | |
| **6** | 0.8 ± 0.1 | 2.4 ± 0.2 | - | - |
| Two component systems (transporter + co-transporter) | | | | |
| **1 + DMT-dT** | 0.8 ± 0.1 | 0.9 ± 0.1 | 6.8 ± 0.4 | 2.7 ± 0.5 |
| **2 + DMT-dT** | 1.6 ± 0.1 | 0.7 ± 0.1 | 9.8 ± 0.6 | 1.4 ± 0.2 |
| **3 + DMT-dT** | 3.0 ± 0.2 | 1.0 ± 0.1 | 10.6 ± 0.4 | 3.6 ± 0.6 |
| **4 + DMT-dT** | 0.5 ± 0.1 | 1.6 ± 0.1 | 5.9 ± 0.3 | 2.8 ± 0.4 |
| **5 + DMT-dT** | 2.4 ± 0.1 | 1.6 ± 0.1 | 10.1 ± 0.5 | 2.1 ± 0.3 |
| **6 + DMT-dT** | 0.16 ± 0.01 | 1.9 ± 0.1 | 6.7 ± 0.5 [d] | 1.7 ± 0.36 [d] |

[a] Concentration of **DMT-dT** was fixed at 10 mol% and the concentration of transporter was varied. [b] Concentration of transporter was fixed at 3 mol% and the concentration of **DMT-dT** was varied. [c] $EC_{50}$: concentration of transporter (mol% with respect to lipid) needed to obtain 50% cAMP influx in 50 min. Corresponds to the *k* value of the fit. [d] Concentration of **6** was fixed at 0.5 mol% and the concentration of **DMT-dT** was varied.

## *S5.5. SPBA assay for cAMP transport: Hill plot for co-transporters*

The cAMP transport assay was performed as described in **section S5.1** for various concentrations of **DMT-dT** co-transporter, while maintaining constant transporter concentration (3 mol% with respect to lipid for transporters **1-5**, and 0.5 mol% with respect to lipid for transporter **6**). Data workup was as described above in **section S5.4** (with END value fixed to 1, and START value fixed to the value obtained for transporter only), and the results are shown in **Figure S32**-**Figure S37** and summarized in **Table S2**.

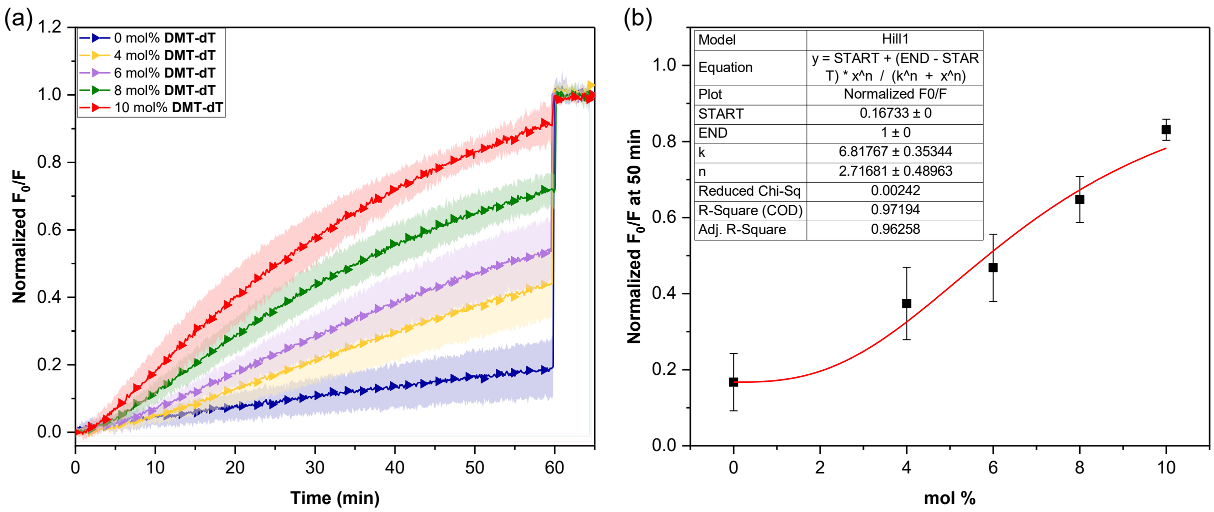


**Figure S32.** (a) cAMP influx mediated by transporter **1** with different **DMT-dT** concentrations from 200 nm unilamellar POPC vesicles loaded with 0.8 mM SPBA, 225 mM $NaNO_3$ and 10 mM HEPES at pH 7.4. Concentrations used: 3 mol% transporter and various mol% **DMT-dT** to lipid. The experiments were performed as described in **section S5.1.** and is the average of minimum of 3 repeats. (shaded areas represent standard deviations). (b) Hill curves plotted from the cAMP influx values at 50 min.

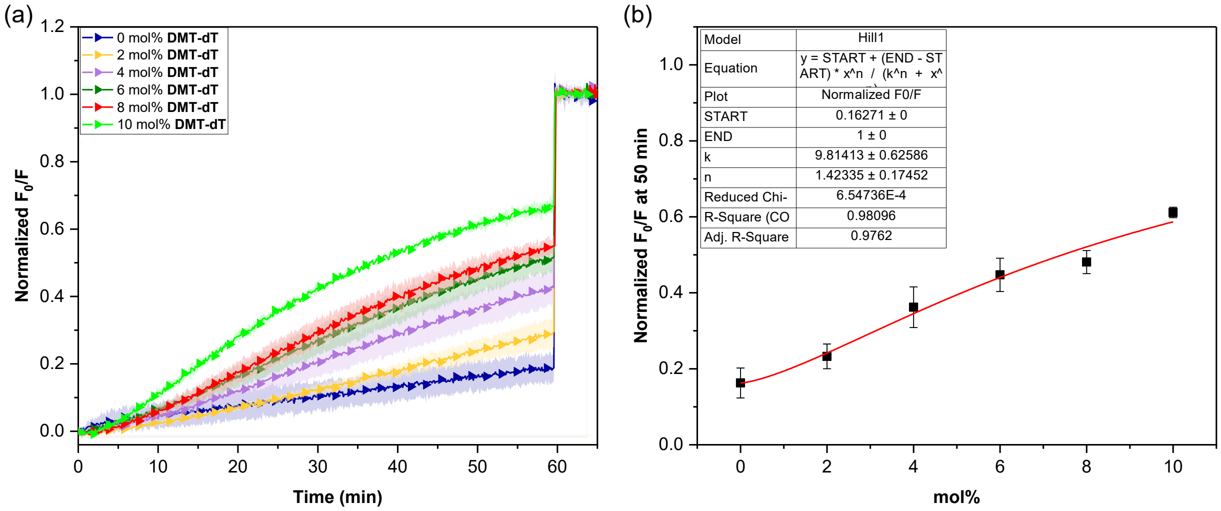


**Figure S33**. (a) cAMP influx mediated by transporter **2** with different **DMT-dT** concentrations from 200 nm unilamellar POPC vesicles loaded with 0.8 mM SPBA, 225 mM $NaNO_3$ and 10 mM HEPES at pH 7.4. Concentrations used: 3 mol% transporter and various mol% **DMT-dT** to lipid. The experiments were performed as described in **section S5.1.** and is the average of minimum of 3 repeats. (shaded areas represent standard deviations). (b) Hill curves plotted from the cAMP influx values at 50 min.

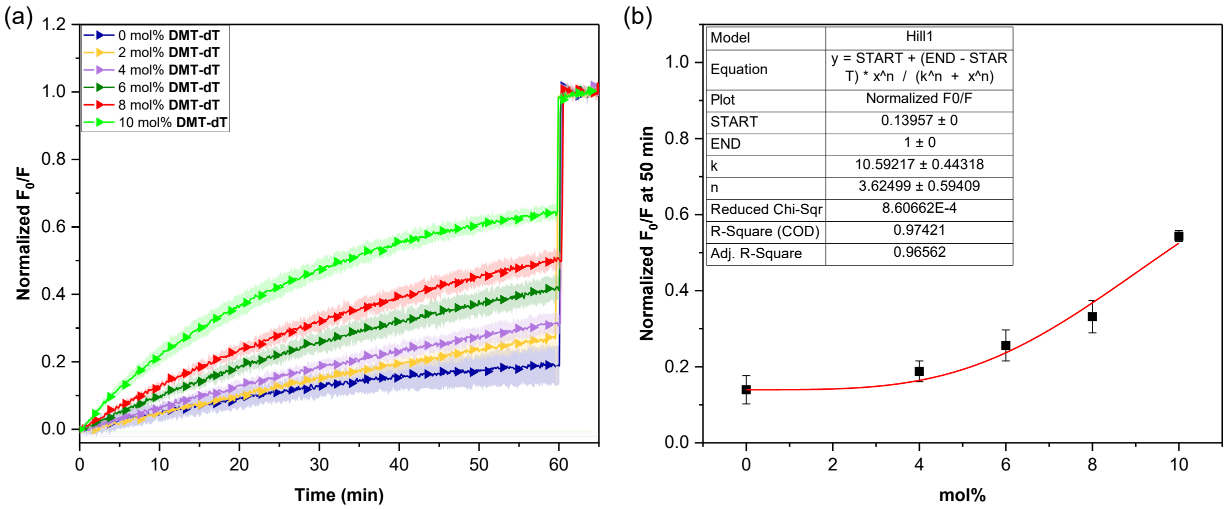


**Figure S34**. (a) cAMP influx mediated by transporter **3** with different **DMT-dT** concentrations from 200 nm unilamellar POPC vesicles loaded with 0.8 mM SPBA, 225 mM $NaNO_3$ and 10 mM HEPES at pH 7.4. Concentrations used: 3 mol% transporter and various mol% **DMT-dT** to lipid. The experiments were performed as described in **section S5.1.** and is the average of minimum of 3 repeats. (shaded areas represent standard deviations). (b) Hill curves plotted from the cAMP influx values at 50 min.

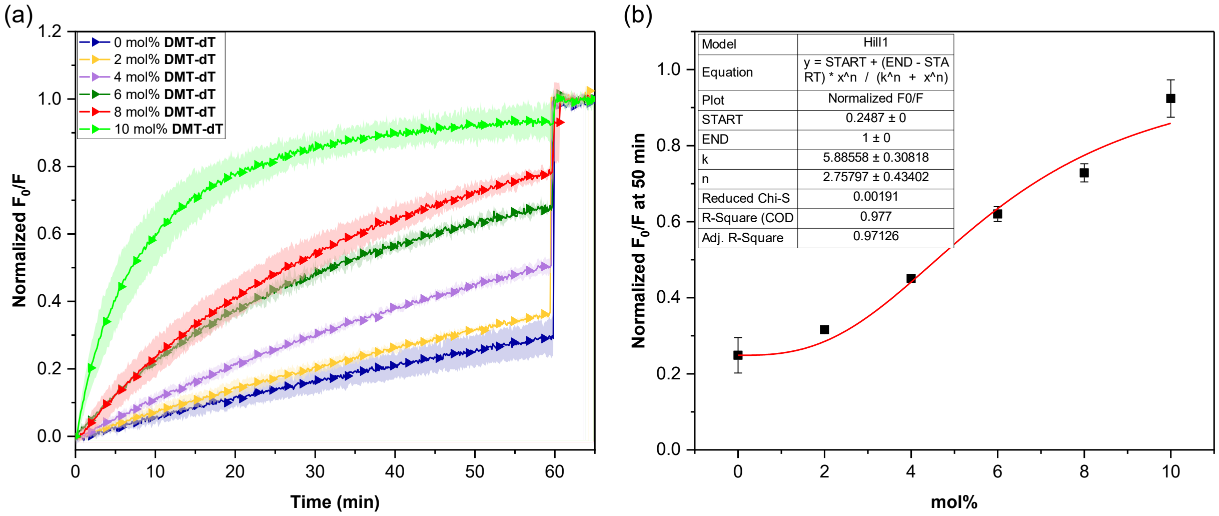


**Figure S35**. (a) cAMP influx mediated by transporter **4** with different **DMT-dT** concentrations from 200 nm unilamellar POPC vesicles loaded with 0.8 mM SPBA, 225 mM $NaNO_3$ and 10 mM HEPES at pH 7.4. Concentrations used: 3 mol% transporter and various mol% **DMT-dT** to lipid. The experiments were performed as described in **section S5.1.** and is the average of minimum of 3 repeats. (shaded areas represent standard deviations). (b) Hill curves plotted from the cAMP influx values at 50 min.

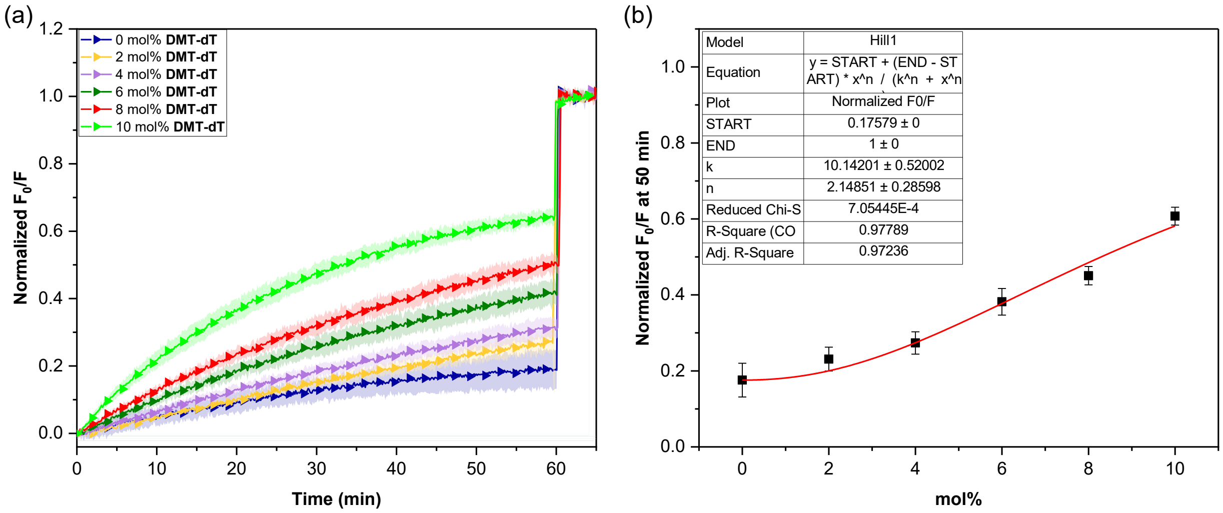


**Figure S36**. (a) cAMP influx mediated by transporter **5** with different **DMT-dT** concentrations from 200 nm unilamellar POPC vesicles loaded with 0.8 mM SPBA, 225 mM $NaNO_3$ and 10 mM HEPES at pH 7.4. Concentrations used: 3 mol% transporter and various mol% **DMT-dT** to lipid. The experiments were performed as described in **section S5.1.** and is the average of minimum of 3 repeats. (shaded areas represent standard deviations). (b) Hill curves plotted from the cAMP influx values at 50 min.

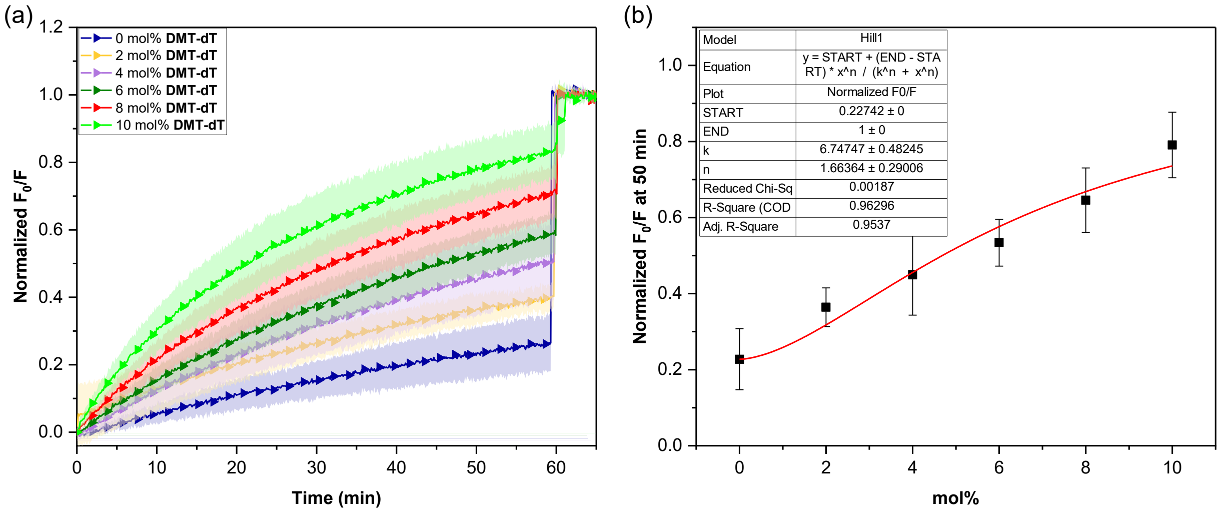


**Figure S37**. (a) cAMP influx mediated by transporter **6** with different **DMT-dT** concentrations from 200 nm unilamellar POPC vesicles loaded with 0.8 mM SPBA, 225 mM $NaNO_3$ and 10 mM HEPES at pH 7.4. Concentrations used: 0.5 mol% transporter and various mol% **DMT-dT** to lipid. The experiments were performed as described in **section S5.1.** and is the average of minimum of 3 repeats. (shaded areas represent standard deviations). (b) Hill curves plotted from the cAMP influx values at 50 min.

## *S5.6. SPBA assay for AMP transport: screening*

The initial screening for AMP transport was conducted using 3 mol% transporter with or without 10 mol% **DMT-dT**. Control experiments were conducted using DMF and **DMT-dT** (10 mol% to lipid) without the transporter, serving as blank solutions. Only transporters **1-6** were tested, because transporters **7-10** did not transport cAMP and were therefore unlikely to transport AMP. The results are shown in **Figure S38**-**Figure S43**. Only transporter **6** in the presence of 10 mol% **DMT-dT** is able to transport AMP.

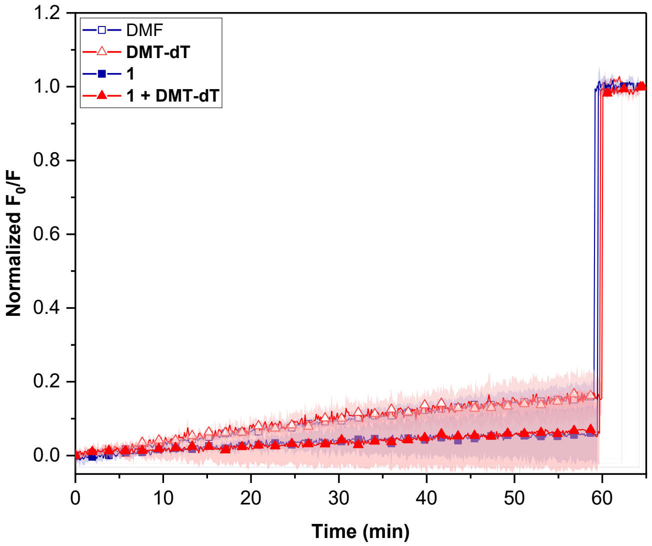


**Figure S38.** AMP transport across 200 nm POPC LUVs mediated by transporter **1** (3 mol% to lipid) along with co-transporters **DMT-dT** (10 mol% to lipid). The experiment was performed as described in **section S5.1.** and is the average of minimum of 3 repeats (shaded areas represent standard deviations).

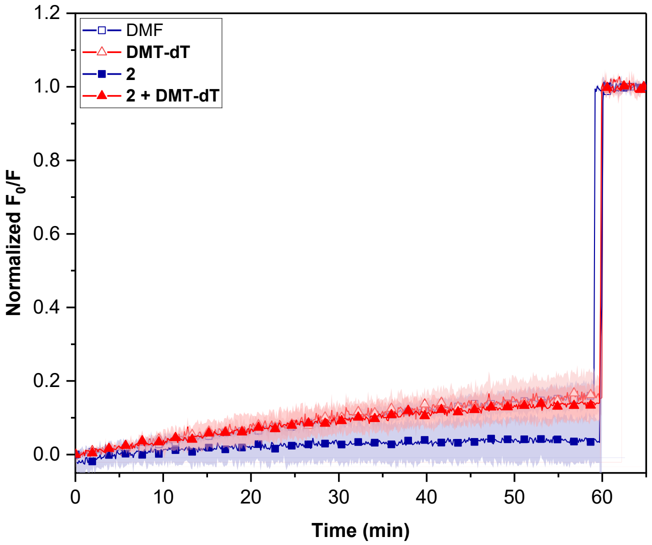


**Figure S39.** AMP transport across 200 nm POPC LUVs mediated by transporter **2** (3 mol% to lipid) along with co-transporters **DMT-dT** (10 mol% to lipid). The experiment was performed as described in **section S5.1.** and is the average of minimum of 3 repeats (shaded areas represent standard deviations).

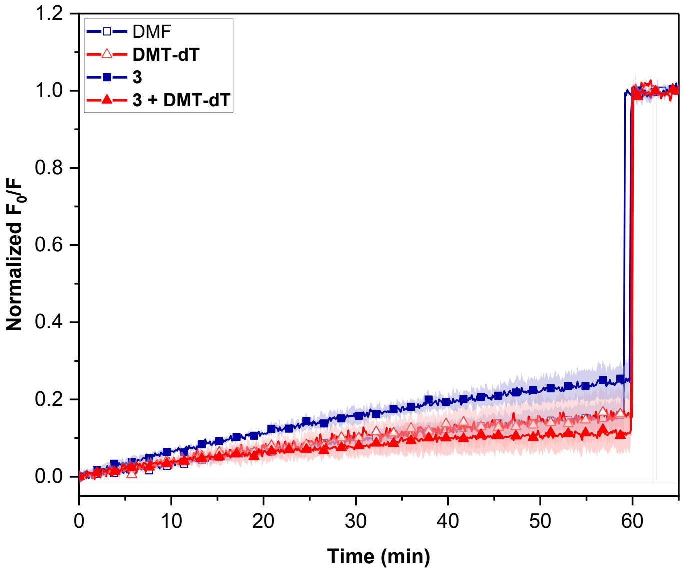


**Figure S40.** AMP transport across 200 nm POPC LUVs mediated by transporter **3** (3 mol% to lipid) along with co-transporters **DMT-dT** (10 mol% to lipid). The experiment was performed as described in **section S5.1.** and is the average of minimum of 3 repeats (shaded areas represent standard deviations).

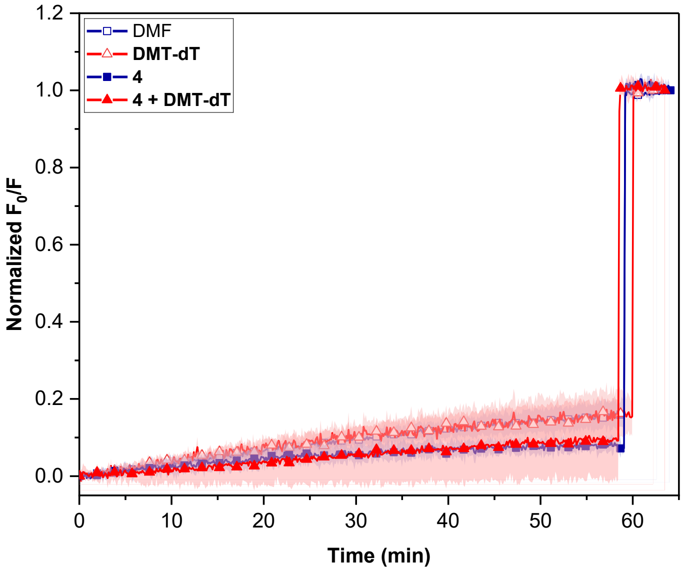


**Figure S41.** AMP transport across 200 nm POPC LUVs mediated by transporter **4** (3 mol% to lipid) along with co-transporters **DMT-dT** (10 mol% to lipid). The experiment was performed as described in **section S5.1.** and is the average of minimum of 3 repeats (shaded areas represent standard deviations).

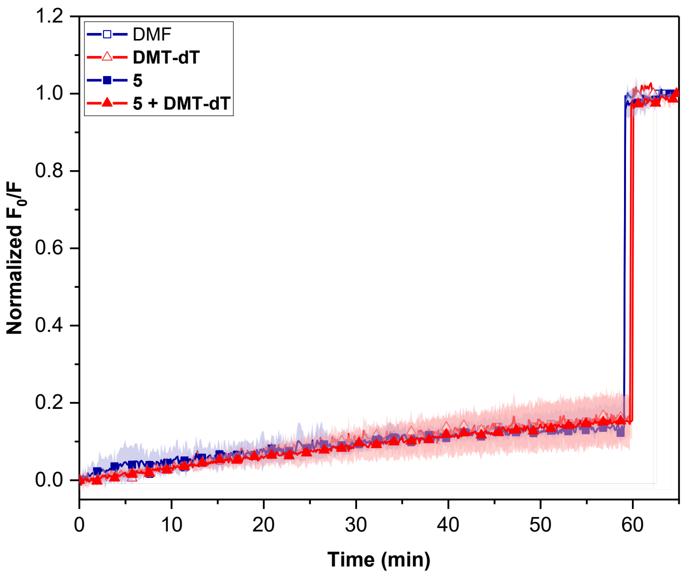


**Figure S42.** AMP transport across 200 nm POPC LUVs mediated by transporter **5** (3 mol% to lipid) along with co-transporters **DMT-dT** (10 mol% to lipid). The experiment was performed as described in **section S5.1.** and is the average of minimum of 3 repeats (shaded areas represent standard deviations).

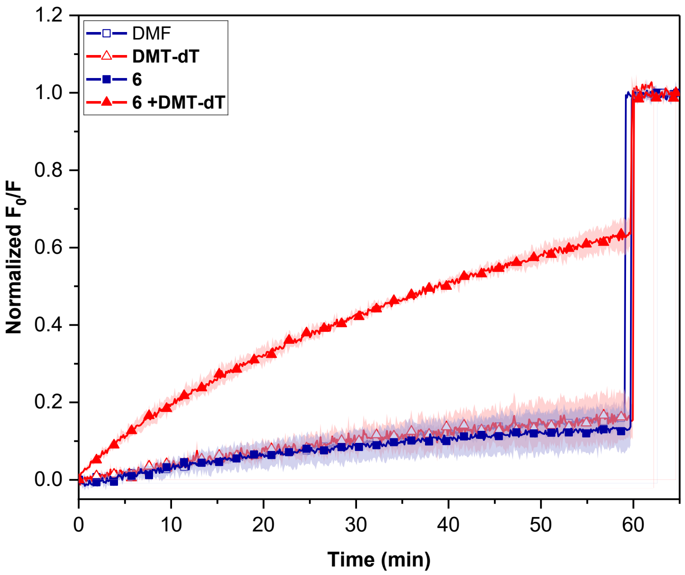


**Figure S43.** AMP transport across 200 nm POPC LUVs mediated by transporter **6** (3 mol% to lipid) along with co-transporters **DMT-dT** (10 mol% to lipid). The experiment was performed as described in **section S5.1.** and is the average of minimum of 3 repeats (shaded areas represent standard deviations).

## *S5.7. SPBA assay for AMP transport: pH dependence*

We also tried to assess the effect of pH on AMP transport. Based on the Chemicalize calculations (**section S2**), AMP is mostly present as a doubly negatively charged species at pH 7.4, while a large amount of singly negatively charged species should be present at pH 6.4. The AMP transport assay was therefore performed as described in **section S6.1**., but both internal and external buffers were adjusted to pH 6.4, 7.4 or 8.4. The experiment was performed using 3 mol% transporter with 10 mol% **DMT-dT**. Control experiments were conducted using **DMT-dT** (10 mol% to lipid) without the transporter, serving as blank solutions. The results are shown in **Figure S44**. Surprisingly, no significant pH dependence was observed for AMP transport.

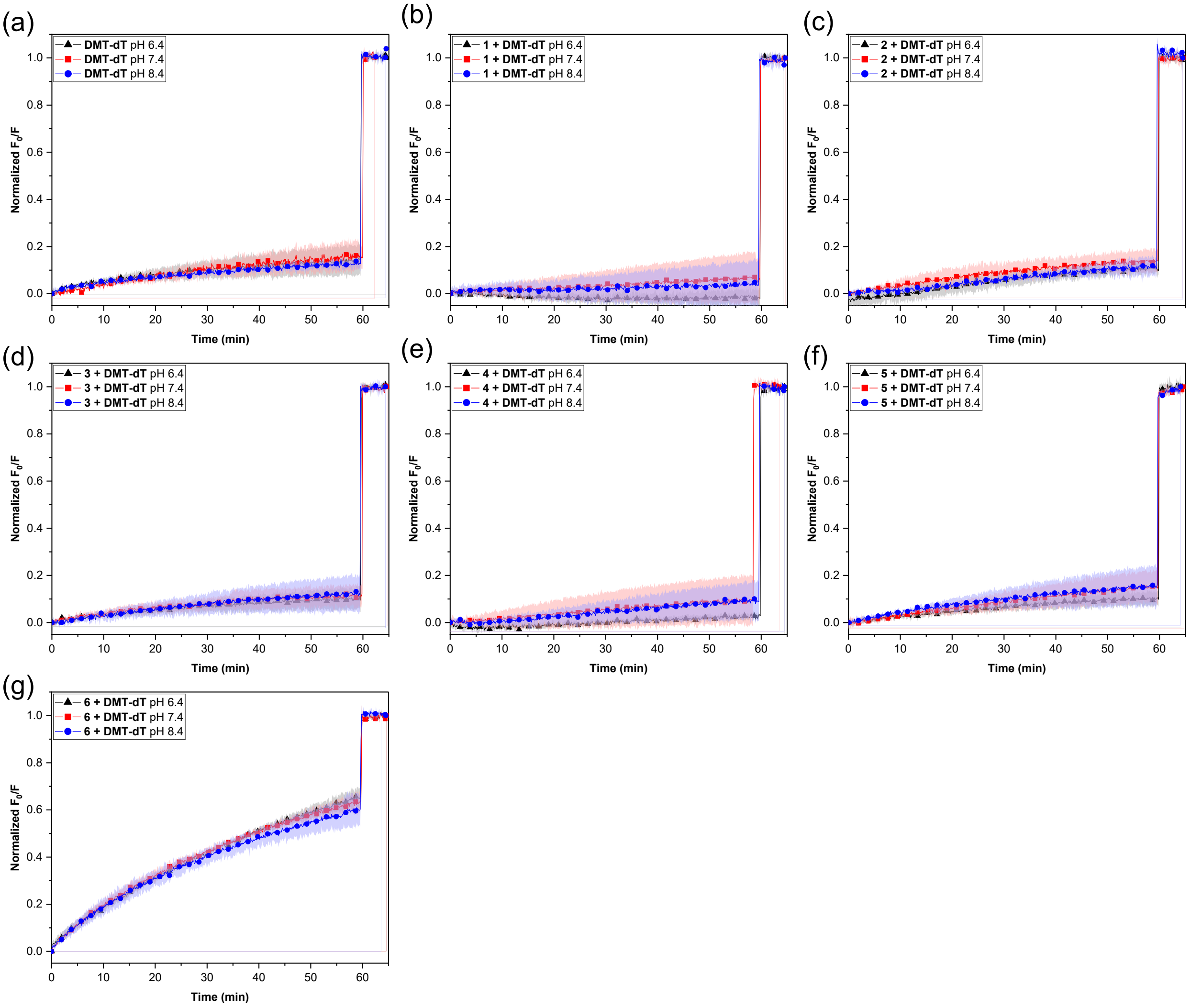


**Figure S44.** AMP transport across 200 nm POPC LUVs mediated by transporters **1-6** with **DMT-dT** at pH 6.4, pH 7.4 and pH 8.4. The experiment was performed as described in **section S5.7.** and is the average of minimum of 3 repeats (shaded areas represent standard deviations). DMT blank and transporters **1-6** (3 mol% to lipid) with **DMT-dT** (10 mol% to lipid) are shown in graphs **a-g** in order.

# S6. Evaluating potential factors leading to false positive results

## *S6.1. Addition of nucleotide solution at different times*

The results shown in the previous sections could be the result of either cAMP or AMP being transported into the liposomes, or the dye (SPBA) being transported out of the liposomes. To verify that the results are due to true nucleotide transport and not dye leakage, we performed an assay where the nucleotide is added to the external solution at various time points after the addition of transporter. If the transporter induces leakage of the dye, the initial fluorescence drop caused by the addition of the nucleotide (due to quenching of any unencapsulated SPBA) will vary depending on the time of nucleotide addition. If there is no leakage the fluorescence drop remains consistent, indicating the amount of dye outside the liposomes has not changed. Furthermore, if there is no change in the rate of cAMP or AMP transport observed, this indicates that there are no significant deliverability problems with these transporters at the given concentrations.

To conduct these experiments, a thin film of POPC lipid was formed by evaporating a chloroform solution of lipid under reduced pressure, followed by drying under high vacuum for at least for 8 hours. The lipid film was hydrated by vortexing with a $NaNO_3$ solution (0.8 mM SPBA in 225 mM $NaNO_3$, 10 mM HEPES at pH 7.4). The lipid suspension was then subjected to eleven freeze-thaw cycles alternating between submersion in liquid nitrogen followed by thawing in a mildly warm water bath (below 34 °C). The lipid suspension was allowed to rest at room temperature for 30 min and was subsequently extruded 29 times through a 200 nm polycarbonate membrane using the Avanti mini extruder set (Avanti Polar Lipids, Inc.). Unencapsulated dye was removed by size exclusion chromatography on a Sephadex G-25 column, eluted with 225 mM $NaNO_3$, 10 mM HEPES, pH 7.4. The final lipid concentration per sample was 0.5 mM.

The dye-loaded liposomes (0.5 mM lipid) were transferred into a 3 mL glass cuvette and placed in the sample compartment of an Agilent Cary Eclipse fluorescence spectrometer equipped with a magnetic stirrer, and a temperature controller. Three separate cuvettes were prepared and placed in the fluorometer. At $t$ = 0 min, fluorescence data collection was started ($\lambda_{ex}$ = 435 nm, $\lambda_{em}$ = 505 nm) and the solutions were stirred. At $t$ = 1 min, 15 µL of the transporter solution in DMF was added to each cuvette. The nucleotide sodium salt (1 M cAMP sodium salt in 225 mM $NaNO_3$ and 10 mM HEPES, pH 7.4 or 1 M AMP sodium salt in 225 mM $NaNO_3$ and 10 mM HEPES, pH 7.4) was then added at different time points: at $t$ = 5 min for the first cuvette, $t$ = 15 min for the second, and $t$ = 25 min for the third cuvette**.** For each cuvette, detergent (75 µL of 10% Triton X-100) was added 60 minutes after the addition of cAMP or AMP to fully lyse the membrane.

The results are plotted as $F/F_0$ as a function of time (whereby $F_0$ is the fluorescence intensity at time $t$ = 0, and $F$ is the fluorescence intensity at any time). The graphs are shown for cAMP transport initiated by **1– 6** in **Figure S45**-**Figure S50** and for AMP transport initiated by **6** in **Figure S51.** It can be seen that the initial drop upon the addition of cAMP or AMP remains the same regardless of the time the nucleotide was added. This indicates that the transporters do not facilitate the efflux of SPBA out of the liposomes. Furthermore, the kinetic traces were fitted to the ExpDecay2 equation using Origin 2024b (10.15), and the extrapolated curves do not coincide with the addition of transporter. This is further proof that the transporters do not cause leakage of SPBA and the observed results are due to true transport of nucleotides.

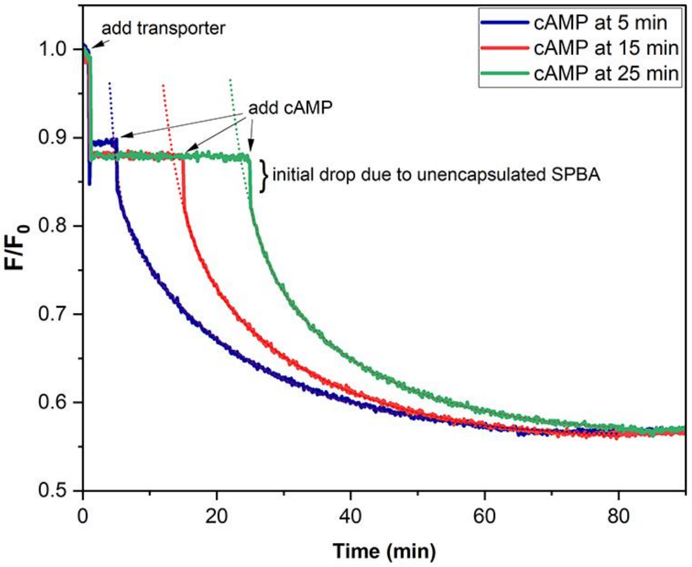


**Figure S45**. cAMP transport across 200 nm POPC LUVs mediated by transporter **1** (3 mol% with respect to lipid) along with the **DMT-dT** co-transporter (10 mol% with respect to lipid). The experiment was performed as described in **section S6.1**. Transporter/co-transporter were added at $t$ = 1 min and cAMP was added at different time points: $t$ = 5 min, $t$ = 15 min or $t$ = 25 min. The sharp drop upon the addition of transporter is due to quenching of SPBA by DMF. The sharp initial drop upon the addition of cAMP is due to quenching of unencapsulated SPBA by cAMP. The fluorescence curve is shown as a solid line, while the simulated ExpDecay2 curve is represented by a dashed line.

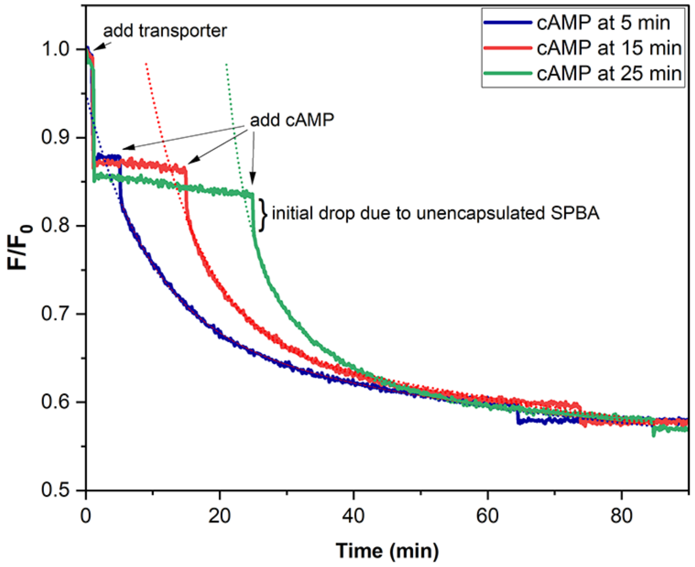


**Figure S46**. cAMP transport across 200 nm POPC LUVs mediated by transporter **2** (3 mol% with respect to lipid) along with the **DMT-dT** co-transporter (10 mol% with respect to lipid). The experiment was performed as described in **section S6.1**. Transporter/co-transporter were added at *t* = 1 min and cAMP was added at different time points: *t* = 5 min, *t* = 15 min or *t* = 25 min. The sharp drop upon the addition of transporter is due to quenching of SPBA by DMF. The sharp initial drop upon the addition of cAMP is due to quenching of unencapsulated SPBA by cAMP. The fluorescence curve is shown as a solid line, while the simulated ExpDecay2 curve is represented by a dashed line.

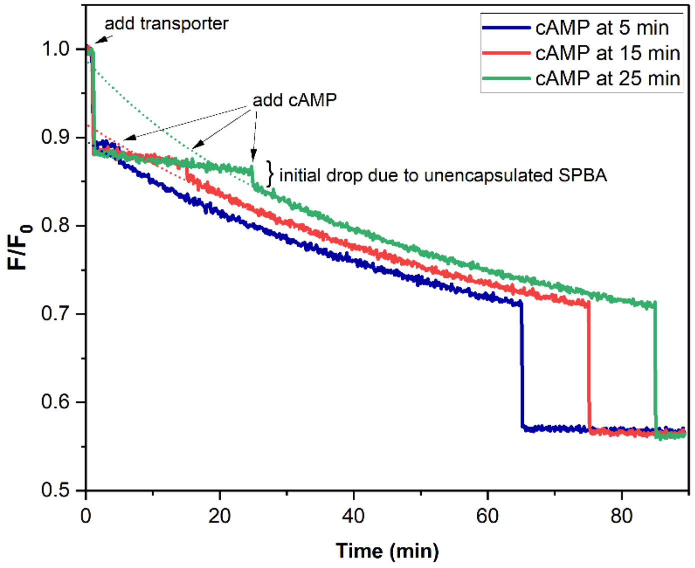


**Figure S47**. cAMP transport across 200 nm POPC LUVs mediated by transporter **3** (3 mol% with respect to lipid) along with the **DMT-dT** co-transporter (10 mol% with respect to lipid). The experiment was performed as described in **section S6.1**. Transporter/co-transporter were added at *t* = 1 min and cAMP was added at different time points: *t* = 5 min, *t* = 15 min or *t* = 25 min. The sharp drop upon the addition of transporter is due to quenching of SPBA by DMF. The sharp initial drop upon the addition of cAMP is due to quenching of unencapsulated SPBA by cAMP. The fluorescence curve is shown as a solid line, while the simulated ExpDecay2 curve is represented by a dashed line.

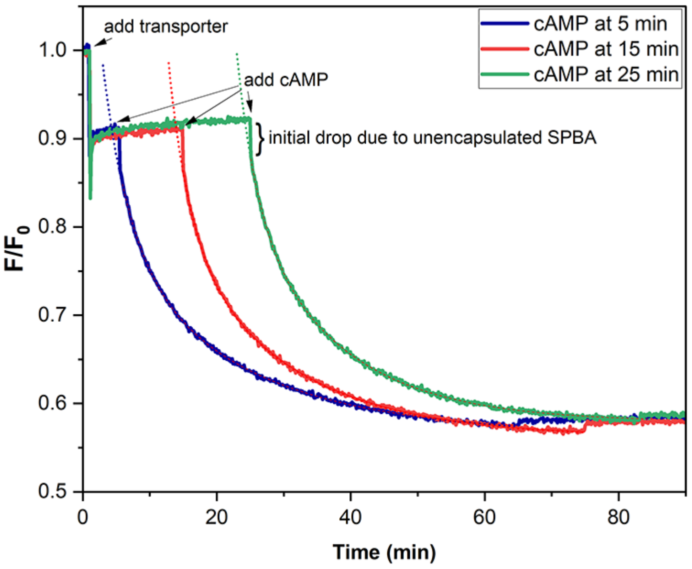


**Figure S48**. cAMP transport across 200 nm POPC LUVs mediated by transporter **4** (3 mol% with respect to lipid) along with the **DMT-dT** co-transporter (10 mol% with respect to lipid). The experiment was performed as described in **section S6.1**. Transporter/co-transporter were added at *t* = 1 min and cAMP was added at different time points: *t* = 5 min, *t* = 15 min or *t* = 25 min. The sharp drop upon the addition of transporter is due to quenching of SPBA by DMF. The sharp initial drop upon the addition of cAMP is due to quenching of unencapsulated SPBA by cAMP. The fluorescence curve is shown as a solid line, while the simulated ExpDecay2 curve is represented by a dashed line.

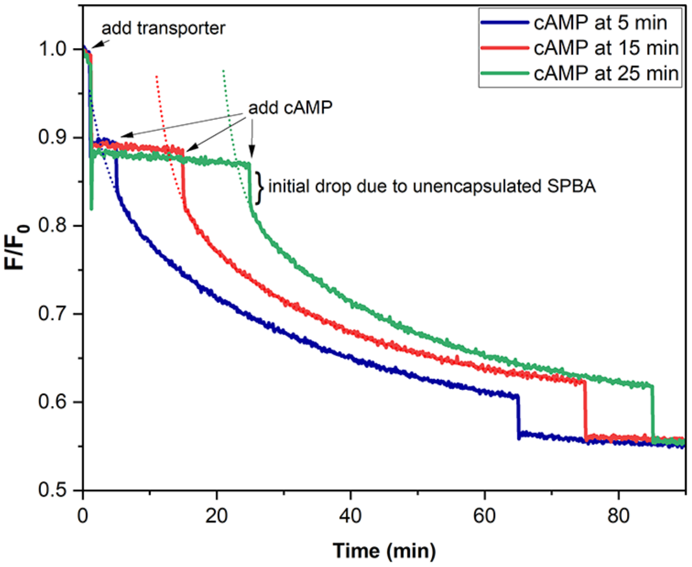


**Figure S49**. cAMP transport across 200 nm POPC LUVs mediated by transporter **5** (3 mol% with respect to lipid) along with the **DMT-Td** co-transporter (10 mol% with respect to lipid). The experiment was performed as described in **section S6.1**. Transporter/co-transporter were added at *t* = 1 min and cAMP was added at different time points: *t* = 5 min, *t* = 15 min or *t* = 25 min. The sharp drop upon the addition of transporter is due to quenching of SPBA by DMF. The sharp initial drop upon the addition of cAMP is due to quenching of unencapsulated SPBA by cAMP. The fluorescence curve is shown as a solid line, while the simulated ExpDecay2 curve is represented by a dashed line.

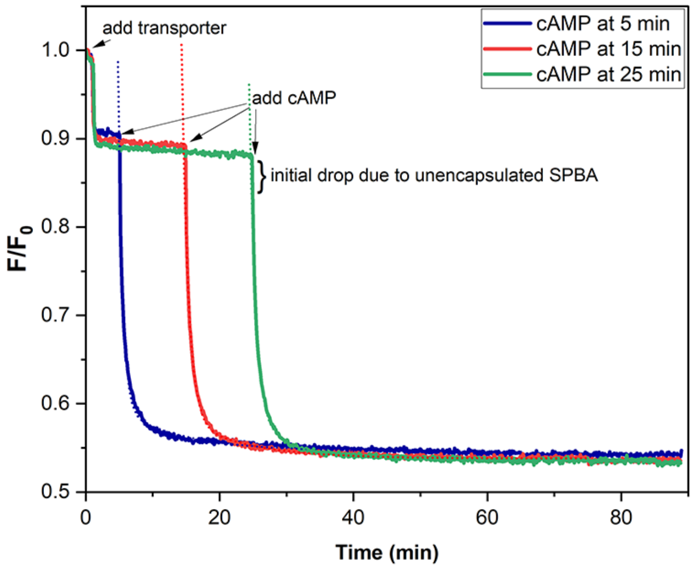


**Figure S50**. cAMP transport across 200 nm POPC LUVs mediated by transporter **6** (3 mol% with respect to lipid) along with the **DMT-dT** co-transporter (10 mol% with respect to lipid). The experiment was performed as described in **section S6.1**. Transporter/co-transporter were added at *t* = 1 min and cAMP was added at different time points: *t* = 5 min, *t* = 15 min or *t* = 25 min. The sharp drop upon the addition of transporter is due to quenching of SPBA by DMF. The sharp initial drop upon the addition of cAMP is due to quenching of unencapsulated SPBA by cAMP. The fluorescence curve is shown as a solid line, while the simulated ExpDecay2 curve is represented by a dashed line.

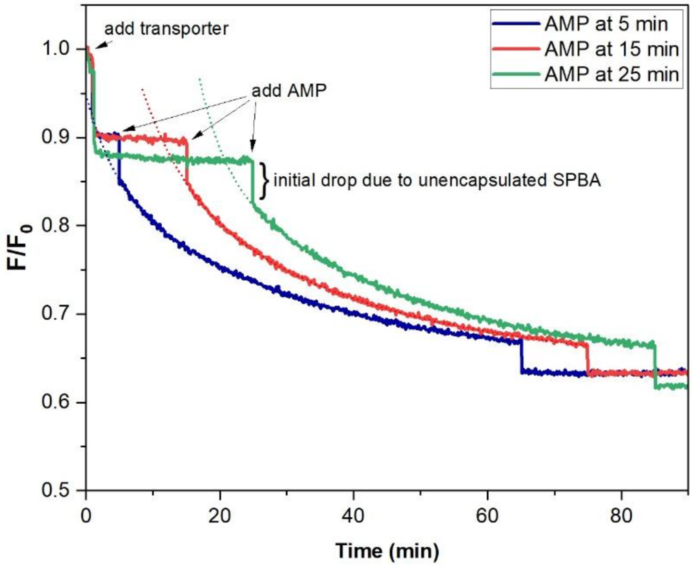


**Figure S51**. AMP transport across 200 nm POPC LUVs mediated by transporter **6** (3 mol% with respect to lipid) along with the **DMT-dT** co-transporter (10 mol% with respect to lipid). The experiment was performed as described in **section S6.1**. Transporter/co-transporter were added at *t* = 1 min and AMP was added at different time points: *t* = 5 min, *t* = 15 min or *t* = 25 min. The sharp drop upon the addition of transporter is due to quenching of SPBA by DMF. The sharp initial drop upon the addition of cAMP is due to quenching of unencapsulated SPBA by AMP. The fluorescence curve is shown as a solid line, while the simulated ExpDecay2 curve is represented by a dashed line.

### *S6.3. Quantifying chloride contamination in nucleotide samples*

SPBA and lucigenin can be quenched by many different anions, and it is therefore important to ensure that the nucleotides are pure enough. A common contaminant that can quench SPBA is chloride. The chloride concentration of each nucleotide stock solution was measured using a chloride-selective electrode. The results indicated that chloride levels in both cAMP and AMP solutions were negligible (**Table S3**). Chloride concentrations in the 1 M nucleotide stocks were in the range of 5-10 mM, which means that in the final experiment where the nucleotide stock is diluted to 25 mM only 125-250 μM chloride will be present. These amounts are not sufficient to induce the level of quenching seen in the transport assays, and the effect of chloride on the transport assay is therefore considered insignificant.

**Table S3.** Measured chloride concentration of each nucleotide stock solutions

| **Nucleotide** | **$Cl^-$ concentration (M)** |
|---|---|
| cAMP (1 M) | 4.3E-3 |
| AMP (1 M) | 9.9E-3 |

## S7. Nucleotide transport studies using $^{31}P$ NMR assay

### *S7.1. General*

For the NMR experiments, a thin film of POPC lipid was formed by evaporating a chloroform solution of lipid under reduced pressure, followed by drying under high vacuum for at least 8 hours. The lipid films were hydrated by vortexing with a $NaNO_3$ solution (225 mM $NaNO_3$, 10 mM HEPES, 10% $D_2O$ at pH 7.4). The lipid suspension was then subjected to eleven freeze-thaw cycles alternating between submersion in liquid nitrogen followed by thawing in a mildly warm water bath (below 34 °C). The lipid suspension was allowed to rest at room temperature for 30 min and was subsequently extruded 29 times through a 200 nm polycarbonate membrane using the Avanti mini extruder set (Avanti Polar Lipids, Inc.). The extruded liposome solution was used without further purification by size exclusion chromatography. The final lipid concentration was 50 mM. A coaxial insert containing trimethyl phosphate ($(CH_3O)_3PO$) in $D_2O$ (50 mM/ 400 μL) was used as the reference and the chemical shift

of its $^{31}$P NMR signal was set at 3.05 ppm. For each transporter, two NMR spectra were recorded: one at time zero ($t$ = 0 h, before addition of transporter) and another one 1 hour after the addition of transporter ($t$ = 1 h), as described in **section S7.2.** $^{31}$P NMR spectra were recorded using a Bruker 600 MHz instrument and were proton decoupled. Acquisition parameters: frequency – 600 MHz; relaxation delay – 1 s; number of scans – 1000; temperature – 298 K, total acquisition time – 40 min.

## *S7.2. Visualization of intravesicular nucleotide by paramagnetic reagent ($MnSO_4$)*

$^{31}$P NMR spectrum at $t$ = 0, without transporters

1.5 mL – 2.0 mL of the liposome solution (50 mM) was prepared as described in **section S7.1** for each experiment. An aliquot of a stock solution of nucleotide sodium salt (1 M in 225 mM $NaNO_3$ and 10 mM HEPES, 10 % $D_2O$, pH 7.4) was added to reach an extravesicular concentration of 100 mM. The solution was stirred for 5 minutes.

500 µL of the liposome solution was taken, and 1 µL of $MnSO_4$ (1 M, $H_2O$) was added to reach a final concentration of 2 mM $MnSO_4$, ensuring the relaxation of the extravesicular nucleotide. The liposome suspension was transferred to an NMR tube containing a coaxial insert filled with trimethyl phosphate as an external reference (50 mM/ 400 µL). The obtained $^{31}$P NMR spectrum is referred to as time zero.

$^{31}$P NMR spectrum at $t$ = 1 h

A DMF solution of the putative transporter (5% of DMF relative to the liposome solution) was added to the remaining liposome suspension to achieve 3 mol% transporter and 10 mol% **DMT-dT** concentrations with respect to lipid. The solution was stirred for 1 hour at room temperature. After stirring, 500 µL of the liposome solution was taken, and 1 µL of $MnSO_4$ (1 M, $H_2O$) was added to reach a final concentration of 2 mM $MnSO_4$, ensuring the relaxation of the extravesicular nucleotide. The second $^{31}$P NMR spectrum was recorded at time 1 h.

$^{31}$P NMR spectra were recorded for cAMP and AMP with transporters **1–6** (3 mol% to lipid) in the presence of **DMT-dT** (10 mol% to lipid). For ADP and ATP, spectra were recorded only for transporter **6** (3 mol%) with **DMT-dT** (10 mol%). In the case of ADP and ATP, an additional experiment was conducted where the liposome solution was stirred for 3 hours after the addition of the transporter solution to investigate whether extended stirring time affects better nucleotide transport. The resulting $^{31}$P NMR spectra are presented in **Figure S52-Figure S55** for each nucleotide. Integrations (sum) of the observed internal cAMP, AMP or ADP peak are referenced to the external reference (50 mM trimethylphosphate), which is set at 100.

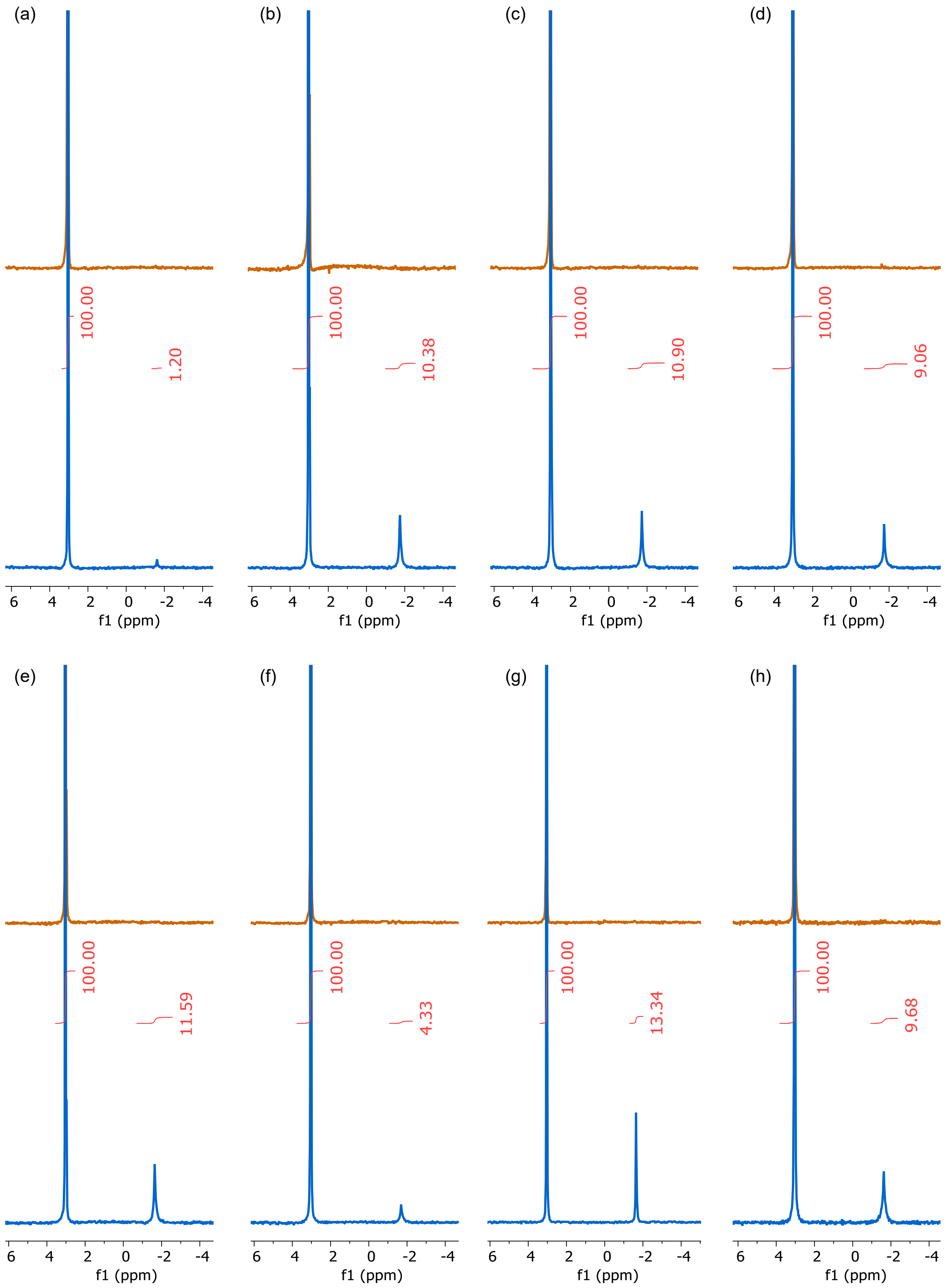


**Figure S52.** $^{31}$P NMR spectra recorded for **cAMP** transport mediated by transporters **1-6** with **DMT-dT**. The experiments were performed as described in **section S7.2**. Recorded spectra (a) **DMT-dT** (10 mol%), (b) transporter **1** (3 mol%) with **DMT-dT** (10 mol%), (c) transporter **2** (3 mol%) with **DMT-dT** (10 mol%), (d) transporter **3** (3 mol%) with **DMT-dT** (10 mol%), (e) transporter **4** (3 mol%) with **DMT-dT** (10 mol%), (f) transporter **5** (3 mol%) with **DMT-dT** (10 mol%), (g) transporter **6** (3 mol%), (h) transporter **6** (3 mol%) with **DMT-dT** (10 mol%). Orange spectra (top) are taken before the addition of transporter, and blue spectra (bottom) are taken 1 h after the addition of transporter. Integrations are relative to the external reference peak (set to 100).

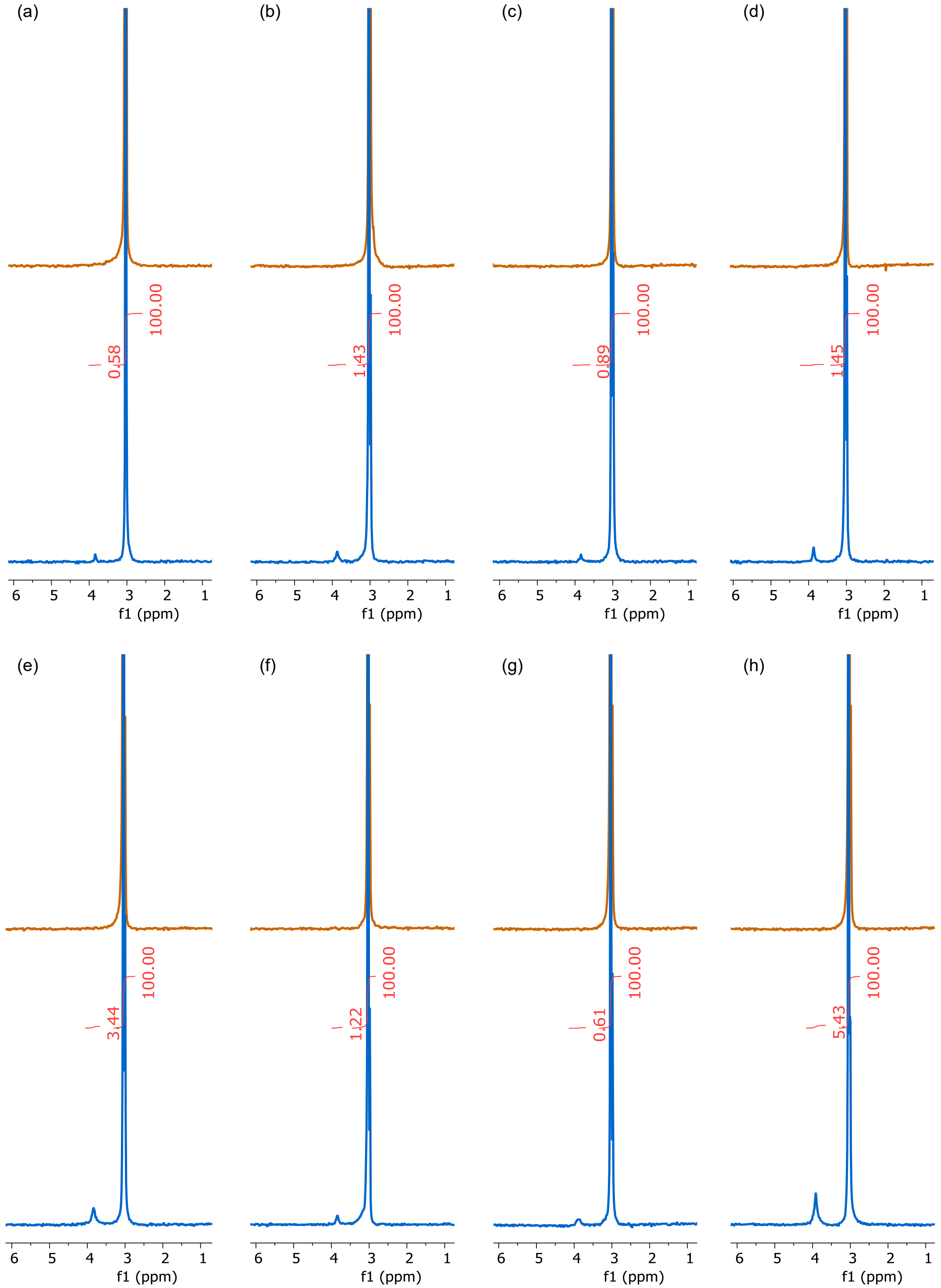


**Figure S53.** $^{31}$P NMR spectra recorded for **AMP** transport mediated by transporters **1-6** with **DMT-dT**. The experiments were performed as described in **section S7.2**. Recorded spectra (a) **DMT-dT** (10 mol%), (b) transporter **1** (3 mol%) with **DMT-dT** (10 mol%), (c) transporter **2** (3 mol%) with **DMT-dT** (10 mol%), (d) transporter **3** (3 mol%) with **DMT-dT** (10 mol%), (e) transporter **4** (3 mol%) with **DMT-dT** (10 mol%), (f) transporter **5** (3 mol%) with **DMT-dT** (10 mol%), (g) transporter **6** (3 mol%), (h) transporter **6** (3 mol%) with **DMT-dT** (10 mol%). Orange spectra (top) are taken before the addition of transporter, and blue spectra (bottom) are taken 1 h after the addition of transporter. Integrations are relative to the external reference peak (set to 100). Vertical scale is the same as *in Figure S52*.

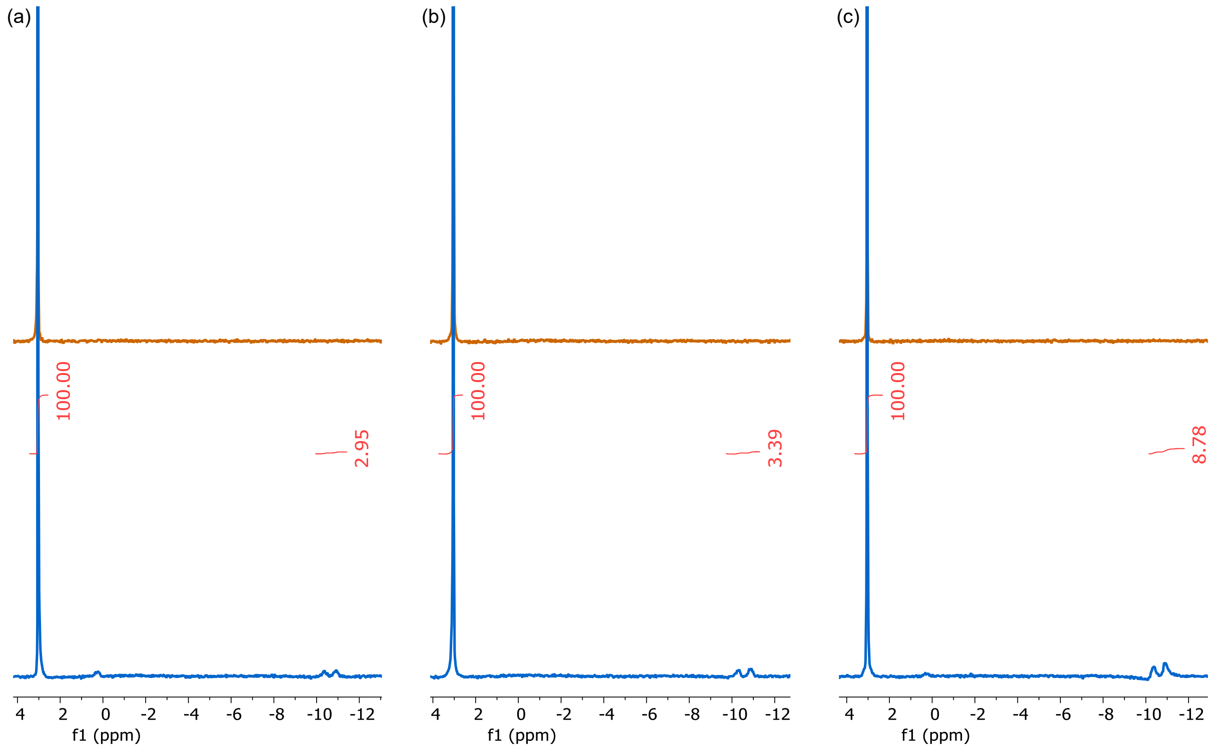


**Figure S54**. $^{31}$P NMR spectra recorded for **ADP** transport mediated by transporter **6** with **DMT-dT**. The experiments were performed as described in **section S7.2**. Recorded spectra (a) **DMT-dT** (10 mol%) after 1 h, (b) transporter **6** (3 mol%) with **DMT-dT** (10 mol%) after 1 h, (c) transporter **6** (3 mol%) with **DMT-dT** (10 mol%) after 3 h. Orange spectra (top) are taken before the addition of transporter, and blue spectra (bottom) are taken 1 h or 3 h after the addition of transporter. Integrations are relative to the external reference peak (set to 100). Vertical scale is the same as *in Figure S52*. The β phosphate peak is not always well reserved and only the α phosphate peak is integrated.

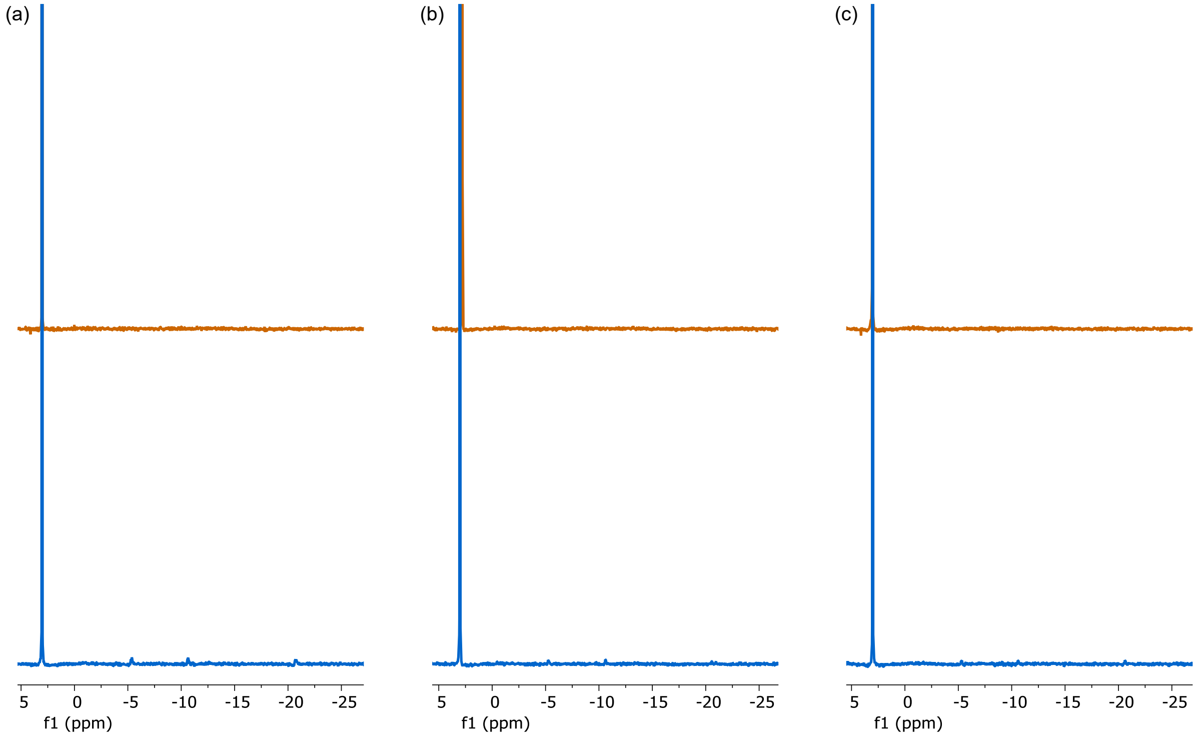


**Figure S55**. $^{31}P$ NMR spectra recorded for **ATP** transport mediated by transporter **6** with **DMT-dT**. The experiments were performed as described in **section S7.2**. Recorded spectra (a) **DMT-dT** (10 mol%) after 1 h, (b) transporter **6** (3 mol%) with **DMT-dT** (10 mol%) after 1 h, (c) transporter **6** (3 mol%) with **DMT-dT** (10 mol%) after 3 h. Orange spectra (top) are taken before the addition of transporter, and blue spectra (bottom) are taken 1 h or 3 h after the addition of transporter. Vertical scale is the same as *in Figure S52*. Peaks were too small to integrate.

## *S7.3. Visualization of intravesicular nucleotide by NMR shift reagent Eu(NO₃)₃*

To confirm that the observed signal in the above experiments is due to intravesicular nucleotide, an additional experiment was performed, in which the NMR shift reagent $Eu(NO_3)_3$ was used instead of $MnSO_4$. The experiment followed the same procedure as described in **section S7.2** but 10 µL of aqueous $Eu(NO_3)_3$ (0.2 M, $H_2O$) was added instead of 1 µL of aqueous $MnSO_4$. The $^{31}P$ NMR spectra were recorded only for cAMP transport with transporters **1–6** (3 mol% with respect to lipid) in the presence of **DMT-T** (10 mol% to lipid). The resulting spectra are presented in **Figure S56.** $Eu^{3+}$ is not a paramagnetic agent (like $Mn^{2+}$), but a shift reagent. Because $Eu^{3+}$ is also not membrane permeable, only external cAMP is shifted to lower chemical shift values (although the exact magnitude of the shift varies from sample to sample), while the internal cAMP peak remains at -1.83 ppm. Integrations (sum) of the observed internal and external cAMP peaks are referenced to the external cAMP peak, which is set to 100, to indicate internal/external cAMP concentration ratios.

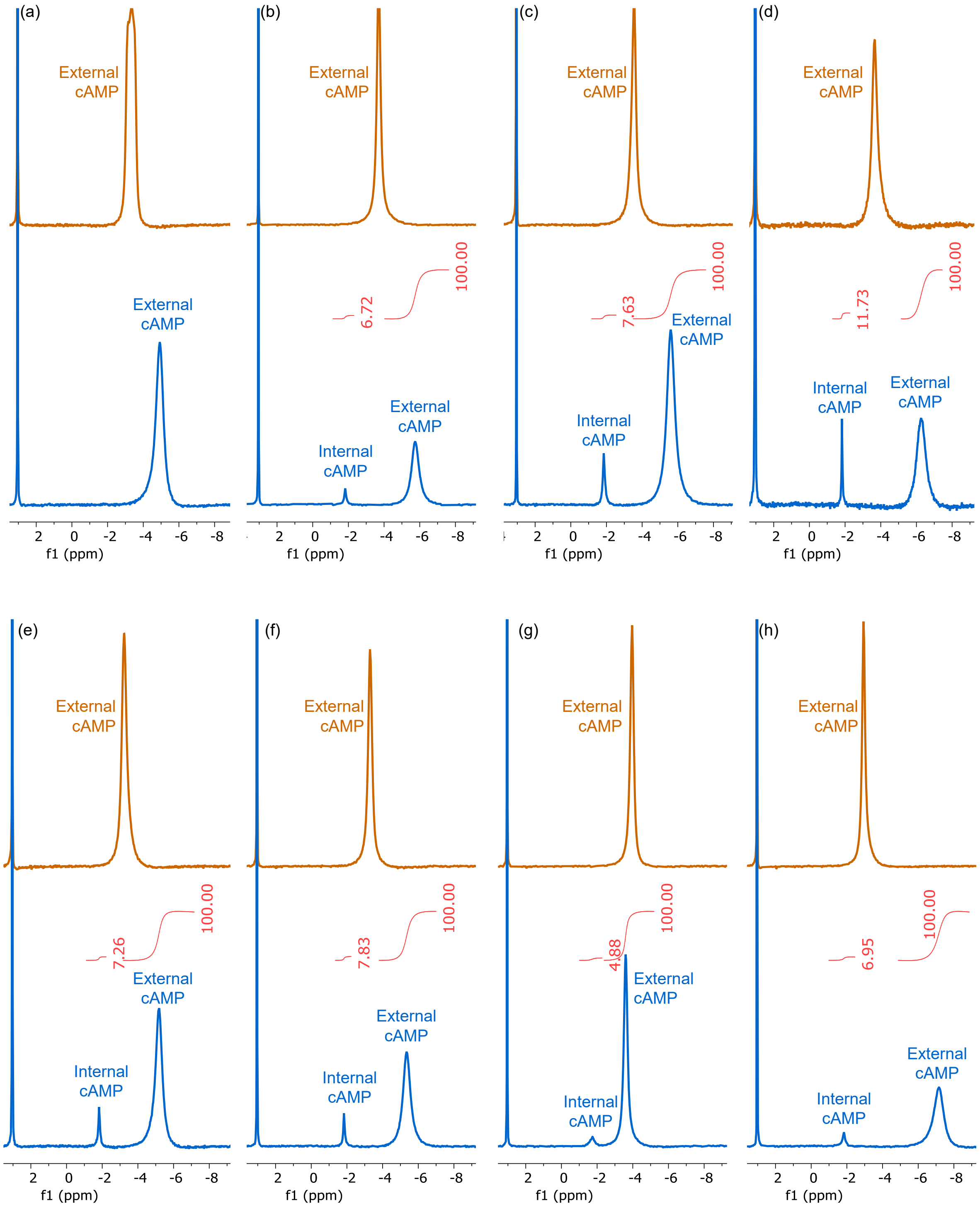


**Figure S56**. $^{31}P$ NMR spectra recorded for **cAMP** transport mediated by transporters **1-6** with **DMT-dT**. The experiments were performed as described in **section S7.3**. Recorded spectra (a) **DMT-dT** (10 mol%), (b) transporter **1** (3 mol%) with **DMT-dT** (10 mol%), (c) transporter **2** (3 mol%) with **DMT-dT** (10 mol%), (d) transporter **3** (3 mol%) with **DMT-dT** (10 mol%), (e) transporter **4** (3 mol%) with **DMT-dT** (10 mol%), (f) transporter **5** (3 mol%) with **DMT-dT** (10 mol%), (g) transporter **6** (3 mol%), (h) transporter **6** (3 mol%) with **DMT-dT** (10 mol%). Orange spectra (top) are taken before the addition of transporter, and blue spectra (bottom) are taken 1 h after the addition of transporter. Integrations are relative to the external cAMP peak (set to 100).

## S.8. $^{1}$H NMR titrations

To determine the association constants for the binding of cAMP anions, $^{1}$H NMR titrations were performed with transporter **1** and transporter **6**. The cAMP anion was used in the form of its TBA (tert-butylammonium) salt, prepared as described in **section S1**. In these experiments, the transporter served as the host and TBA–cAMP served as the guest. For transporter **1**, an additional titration was performed by switching the roles of host and guest. For transporter **6**, when the concentration exceeded 2 mM the obtained association constants became inconsistent due to the tendency of squaramide compounds to aggregate at higher concentrations. As a result, an NMR titration whereby the host and guest roles were switched could not be performed for transporter **6**.

$^{1}$H NMR titrations were performed using a Bruker 600 MHz instrument. A mixture of 0.5% Milli-Q $H_2O$ and 99.5% DMSO-$d_6$ was used to accommodate optimal solubility of both transporters. Titrations were performed using a 2.5 mM or 2 mM solution of host as the starting point for transporter **1** and a 2 mM solution of host as the starting point for transporter **6**, to which aliquots of a solution containing guest (concentration varied in each experiment) and host were added using a Hamilton gas-tight syringe (this procedure ensures that the host concentration remains constant throughout the titration) through a rubber septum. The $^{1}$H NMR spectrum was obtained upon each addition. The instrument was locked to DMSO, and the solvent peak referenced to $\delta$ = 2.50 ppm. The downfield shift in the transporters N-H peaks was determined using MestreNova, and these values were used to calculate association constants ($K_a$) using the online tool BindFit2. All titrations were repeated a minimum of 3 times, and the association constants are given as the average of these 3 repeats with errors representing standard deviations. For transporter **1**, the data fitted well with a 1:1 binding model and the association constant was $K_a$ = 1294 $M^{-1}$ (error 5.77%), however for transporter **6** the data did not conform to a 1:1 binding model, instead a better fit was obtained using 2:1 host-guest binding model (two transporter molecules per cAMP anion) with the association constants $K_{a1}$ = 1046 (error 7.00%) and $K_{a2}$ = 313 (error 35 %).

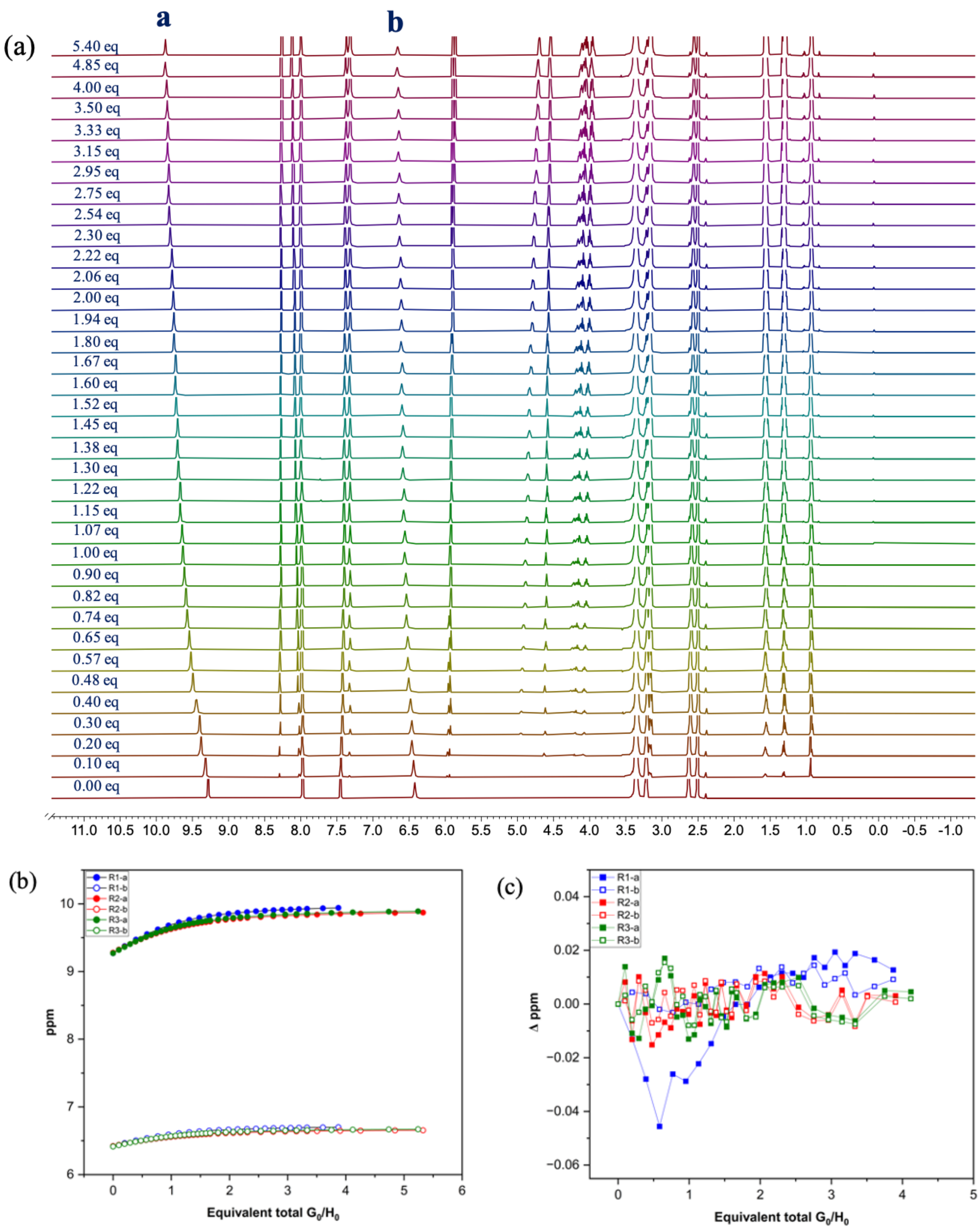


**Figure S57**. $^1$H NMR titration of transporter **1** (host) with TBA-cAMP (guest) in 0.5% Milli-Q $H_2O$:99.5% DMSO-$d_6$ at 298 K. (a) Stack plot of selected spectra of a representative titration (transporter **1**: 2 mM, TBA-cAMP: 20 mM). (b) Fitplot for the NHs at δa = 9.28 ppm and δb = 6.42 ppm using global analysis and 1:1 binding stoichiometry. Data from 3 independent repeats are overlaid (repeat 1. transporter **1**: 2.5 mM, TBA-cAMP: 50 mM, repeat 2. transporter **1**: 2 mM, TBA-cAMP: 20 mM, repeat 3. transporter **1**: 2 mM, TBA-cAMP: 20 mM). $K_{11}$ = 1294 $M^{-1}$ (error 5.77%) (c) Plot of the residuals for NHs at δa = 9.28 ppm and δb = 6.42 ppm using global analysis and 1:1 binding stoichiometry. Data from 3 independent repeats are overlaid.

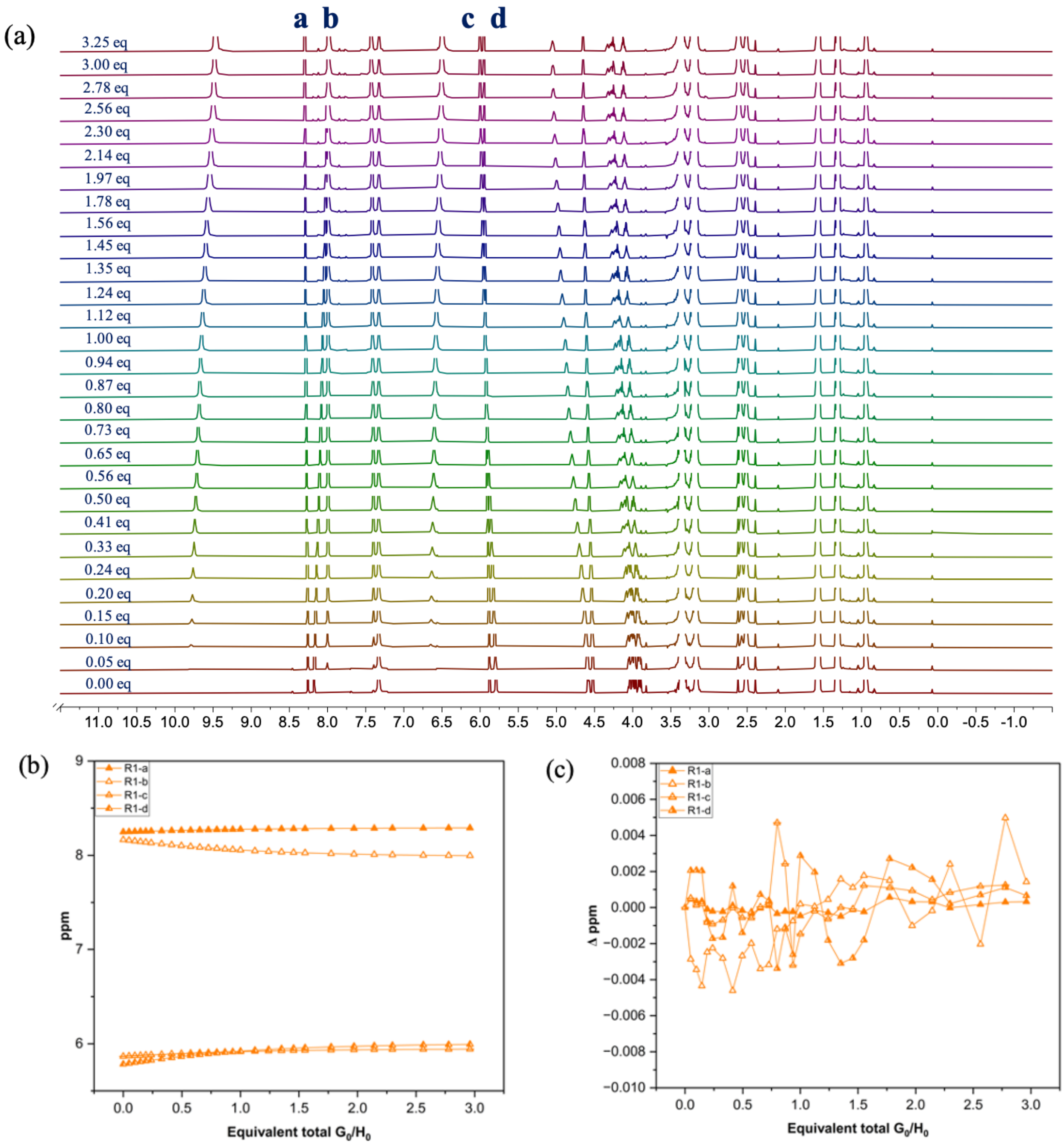


**Figure S58.** $^1$H NMR titration of TBA-cAMP (host) with transporter **1** (guest) in 0.5% Milli-Q $H_2O$:99.5% DMSO-$d_6$ at 298 K. (a) Stack plot of spectra of a representative titration (TBA-cAMP: 2 mM, transporter **1**: 10 mM) (b) Fitplot for the cAMP peaks at δa = 8.25 ppm, δb = 8.17 ppm, δc = 5.86 ppm and δd = 5.78 ppm using global analysis and 1:1 binding stoichiometry. (c) Plot of the residuals for cAMP peaks at δa = 8.25 ppm, δb = 8.17 ppm, δc = 5.86 ppm and δd = 5.78 ppm using global analysis and 1:1 binding stoichiometry.

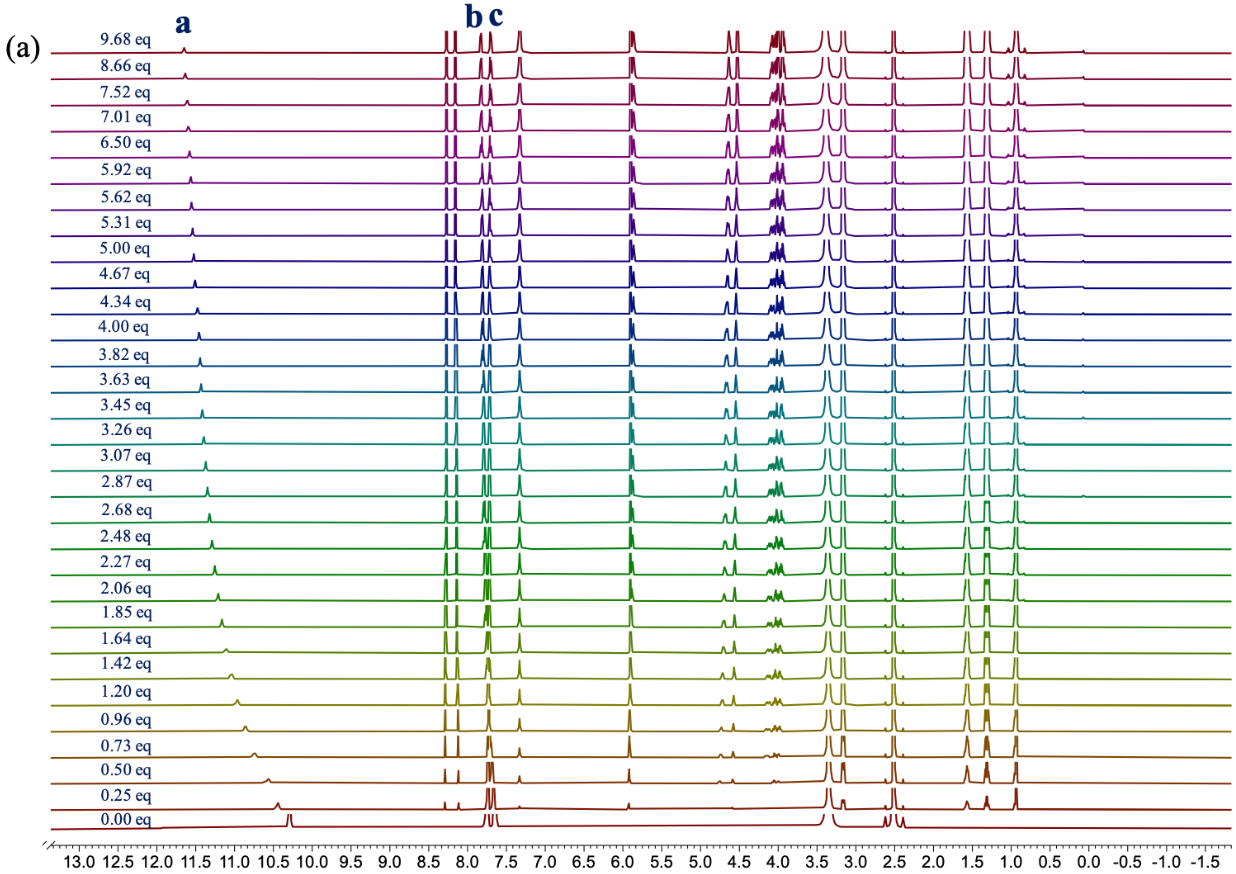


<u>1:1 binding fit</u>

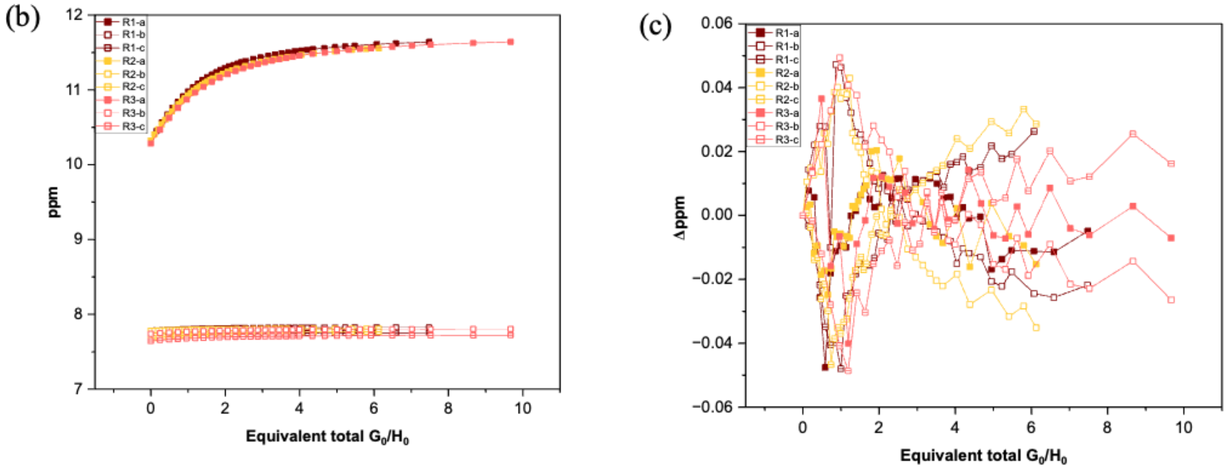

## 2:1 binding fit

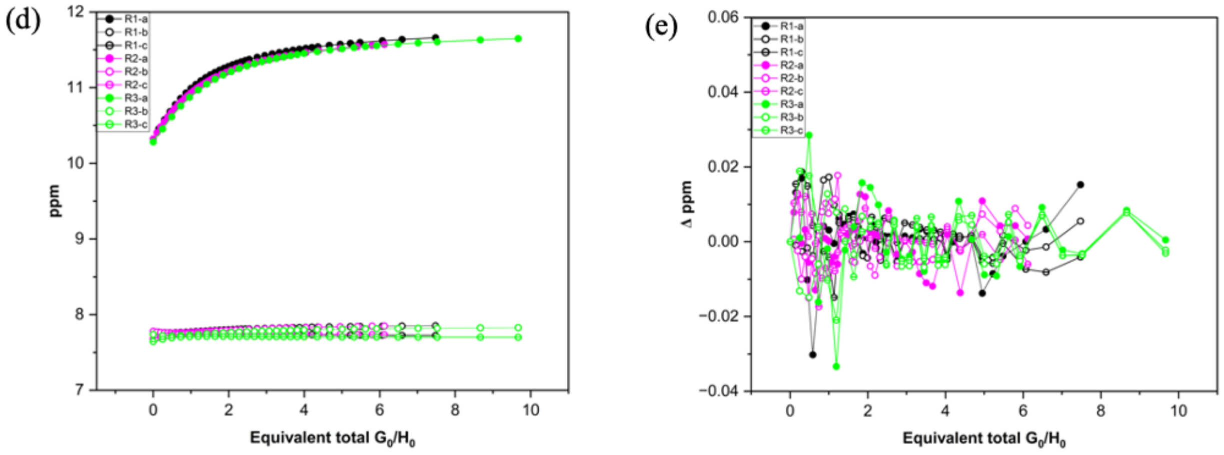


**Figure S59**. $^{1}$H NMR titration of transporter **6** (host) with TBA-cAMP (guest) in 0.5% Milli-Q $H_2O$:99.5% DMSO- at 298 K. (a) Stack plot of selected spectra of a representative titration (transporter **6**: 2 mM, TBA-cAMP: 20 mM). (b) Fitplot for the squaramide NH at δa = 10.29 ppm and phenyl Hs at δb = 7.74 and δc = 7.64 ppm using global analysis and 1:1 binding stoichiometry. Data from 3 independent repeats are overlaid. (repeat 1. transporter **6**: 2 mM, TBA-cAMP: 30.5 mM, repeat 2. transporter **6**: 2 mM, TBA-cAMP: 20 mM, repeat 3. transporter **6**: 2 mM, TBA-cAMP: 50 mM). $K_{a1}$ = 1046 (error 7.00%) and $K_{a2}$ = 313 (error 35 %) (c) Plot of the residuals for squaramide NH at δa = 10.29 ppm and phenyl Hs at δb = 7.74 and δc = 7.64 ppm using global analysis and 1:1 binding stoichiometry. Data from 3 independent repeats are overlaid. (d) Fitplot for the squaramide NH at δa = 10.29 ppm and phenyl Hs at δb = 7.74 and δc = 7.64 ppm using global analysis and 2:1 binding stoichiometry. Data from 3 independent repeats are overlaid as mentioned in 1:1 binding. (c) Plot of the residuals for squaramide NH at δa = 10.29 ppm and phenyl Hs at δb = 7.74 and δc = 7.64 ppm using global analysis and 2:1 binding stoichiometry. Data from 3 independent repeats are overlaid.

## S9. MM calculations

Monte Carlo multiple minimum (MCMM)[7] conformational searches (100 steps per torsion angle, maximum 1000 steps in total) were performed in Schrödinger Release 2023–4, using the OPLS4 force field in Maestro MacroModel (version 13.8.135). The lowest energy conformers for $cAMP^{-}$, $AMP^{-}$, and $AMP^{2-}$ with transporters **1** and **6** (in the absence or presence of **DMT-dT**) are presented in **Figure S60** - **Figure S65.**

As expected, clear hydrogen bonding interactions between transporters **1** and **6** and the phosphate group of the nucleotides are observed in all cases. However, base-pairing between the (c)AMP adenine base and the thymine base of co-transporter **DMT-dT** are rarely seen (except for **1** with $AMP^{-}$ and **DMT-dT**, see **Figure S61b**). Instead, π-π stacking is mostly observed between either thymine and adenine, or thymine and the aromatic ring of the transporters, or between the aromatic rings of the DMT group and the aromatic rings of the transporters. MM calculations without explicit solvent system often overestimate π-π stacking contributions, but it is certainly possible that the complex involved in cAMP and AMP transport does not involve A-T base pair but other types of interactions as well.

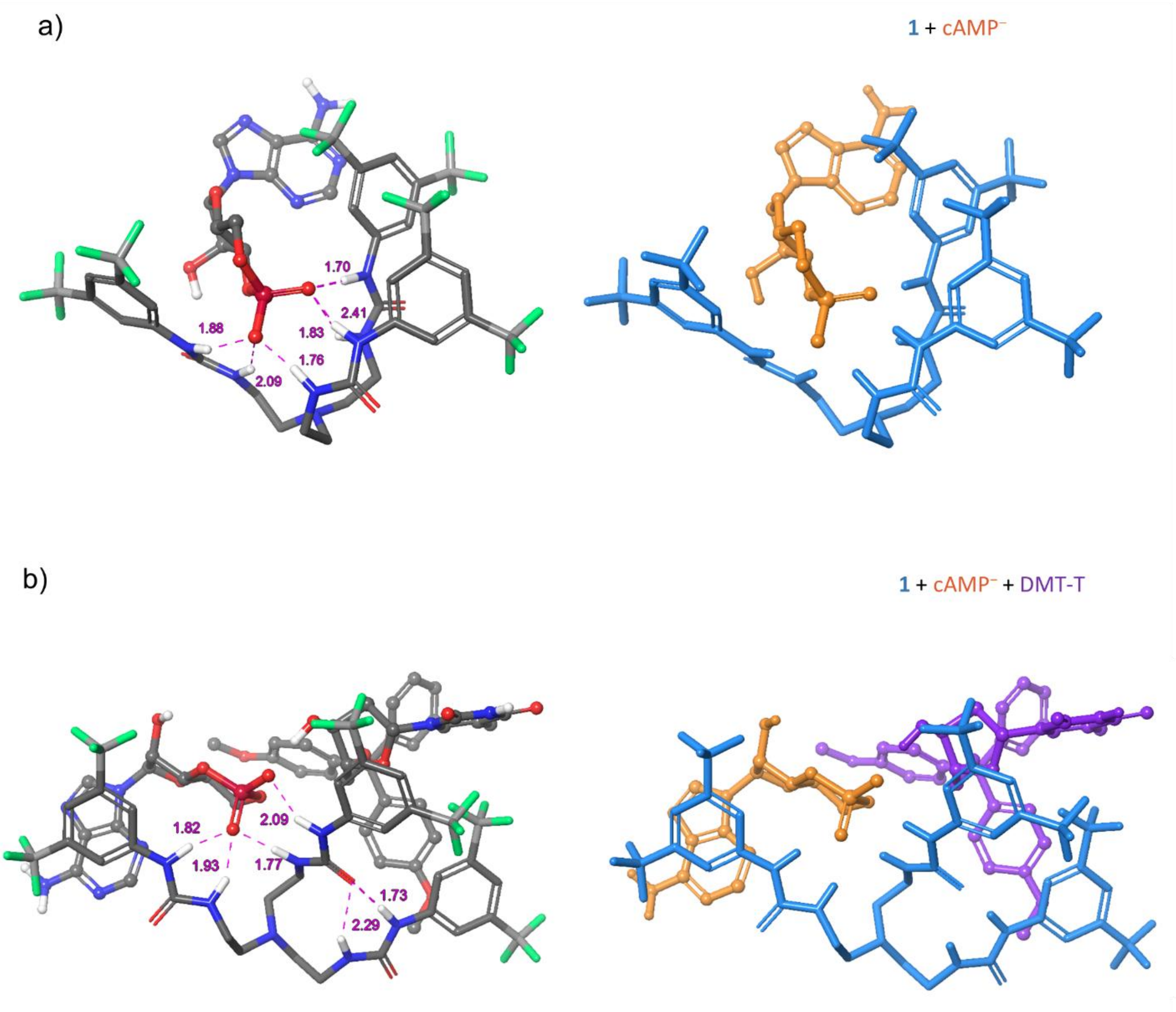


**Figure S60**. Lowest energy conformers with selected distances (Å): (a) Transporter **1** (blue) with cAMP⁻ (orange), and (b) **1** (blue) with cAMP⁻ (orange) and co-transporter **DMT-dT** (purple). All hydrogen atoms linked to carbon atoms are omitted for clarity.

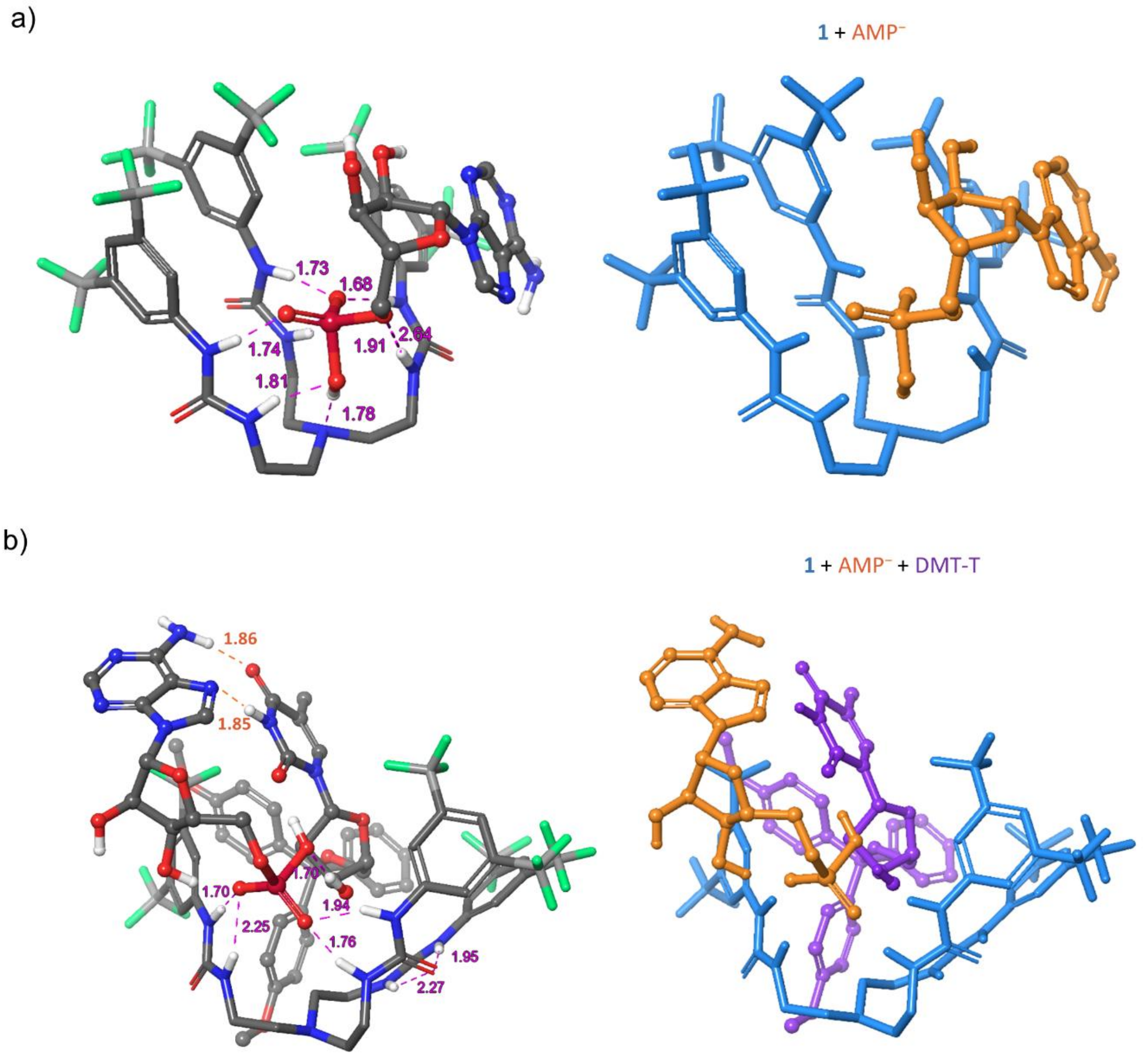


**Figure S61**. Lowest energy conformers with selected distances (Å): (a) Transporter **1** (blue) with AMP⁻ (orange), and (b) **1** (blue) with AMP⁻ (orange) and co-transporter **DMT-dT** (purple). All hydrogen atoms linked to carbon atoms are omitted for clarity.

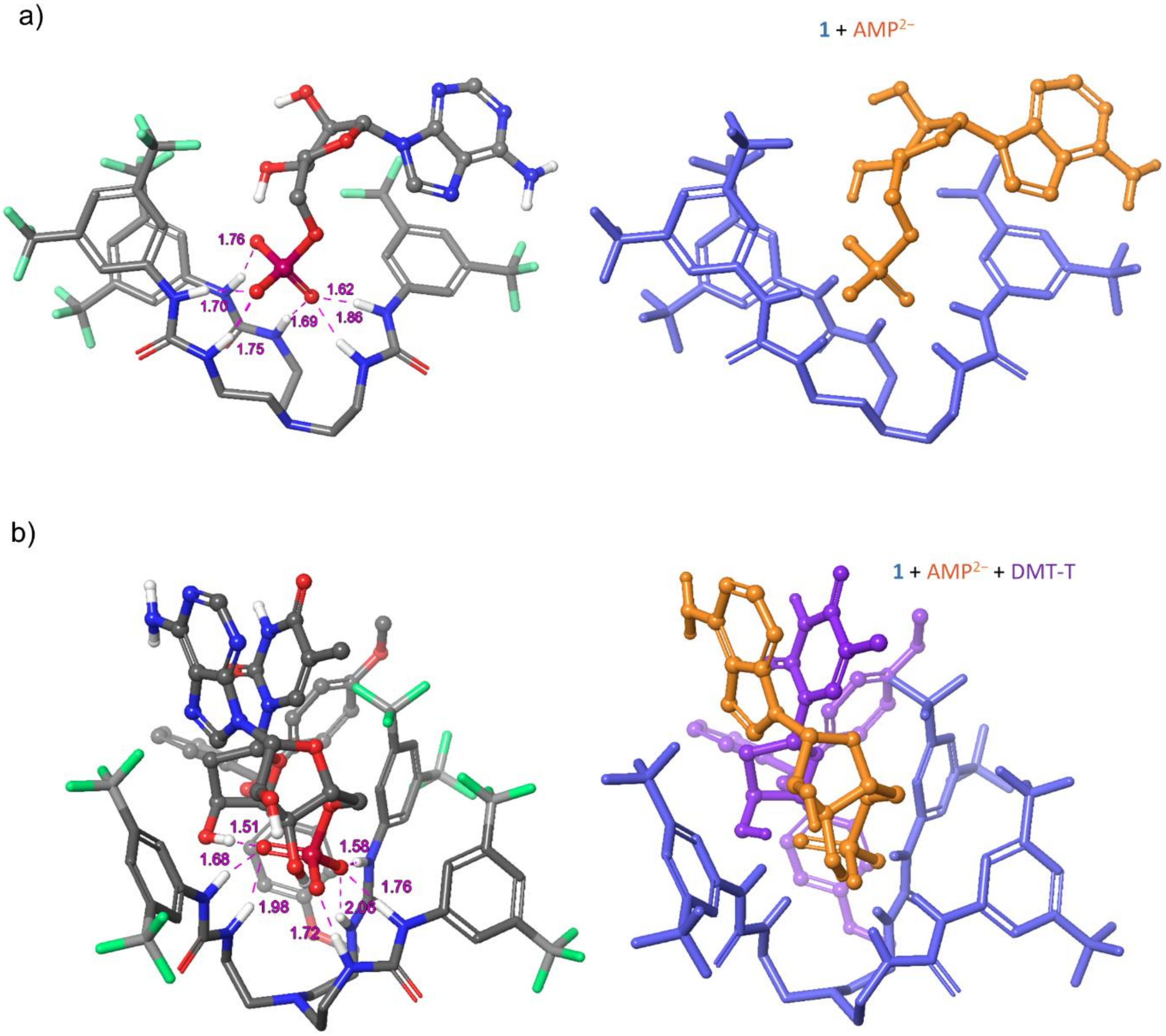


**Figure S62**. Lowest energy conformers with selected distances (Å): (a) Transporter **1** (blue) with $AMP^{2-}$ (orange), and b) **1** (blue) with $AMP^{2-}$ (orange) and co-transporter **DMT-dT** (purple). All hydrogen atoms linked to carbon atoms are omitted for clarity.

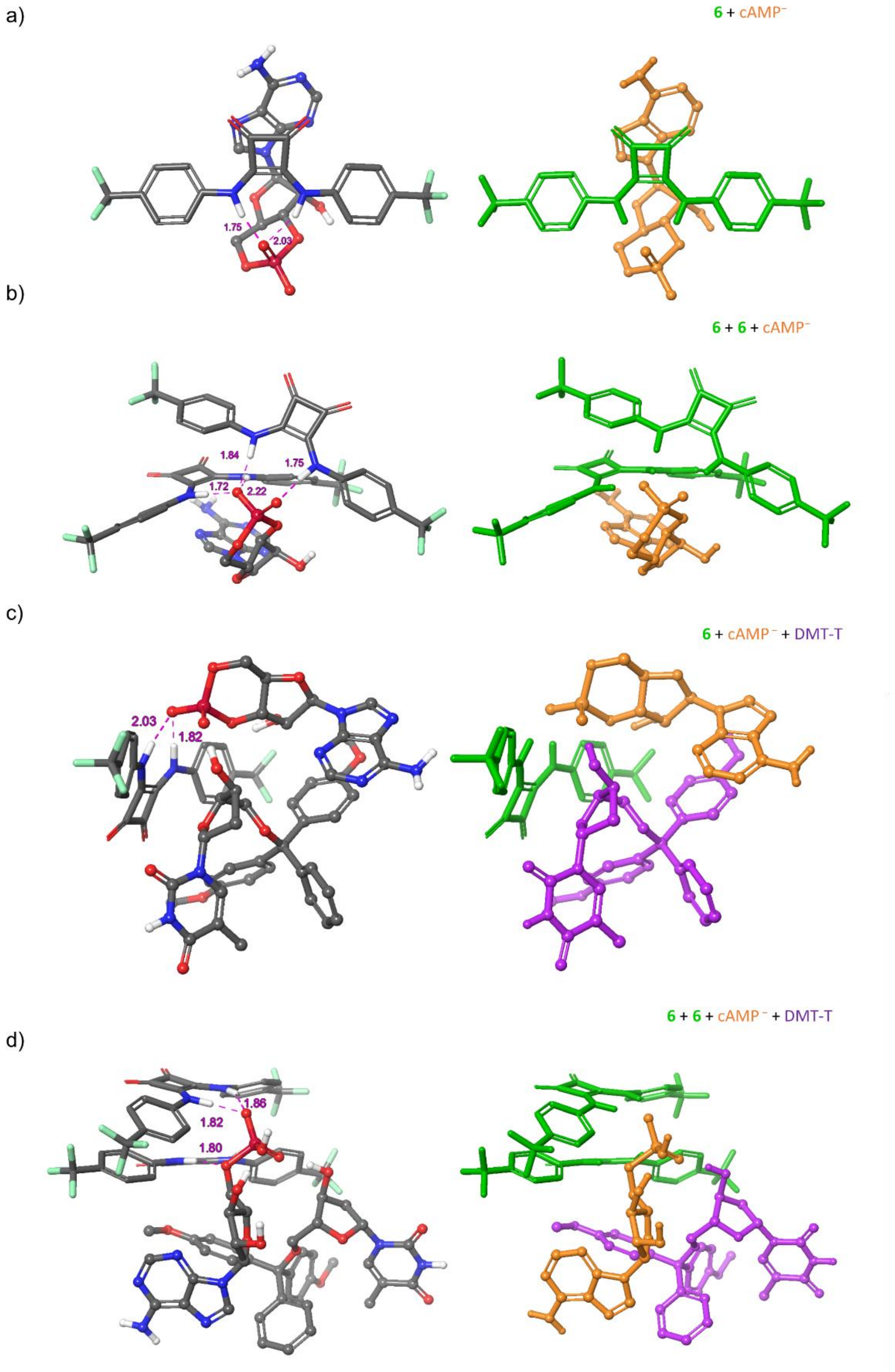


**Figure S63**. Lowest energy conformers with selected distances (Å): (a) Transporter **6** (green) with cAMP⁻ (orange); (b) Two transporters of **6** (both in green) with cAMP⁻ (orange); (c) Transporter **6** (green) with cAMP⁻ (orange) and co-transporter **DMT-dT** (purple); (d) Two transporters of **6** (both in green) with cAMP⁻ (orange) and co-transporter **DMT-dT** (purple). All hydrogen atoms linked to carbon atoms are omitted for clarity.

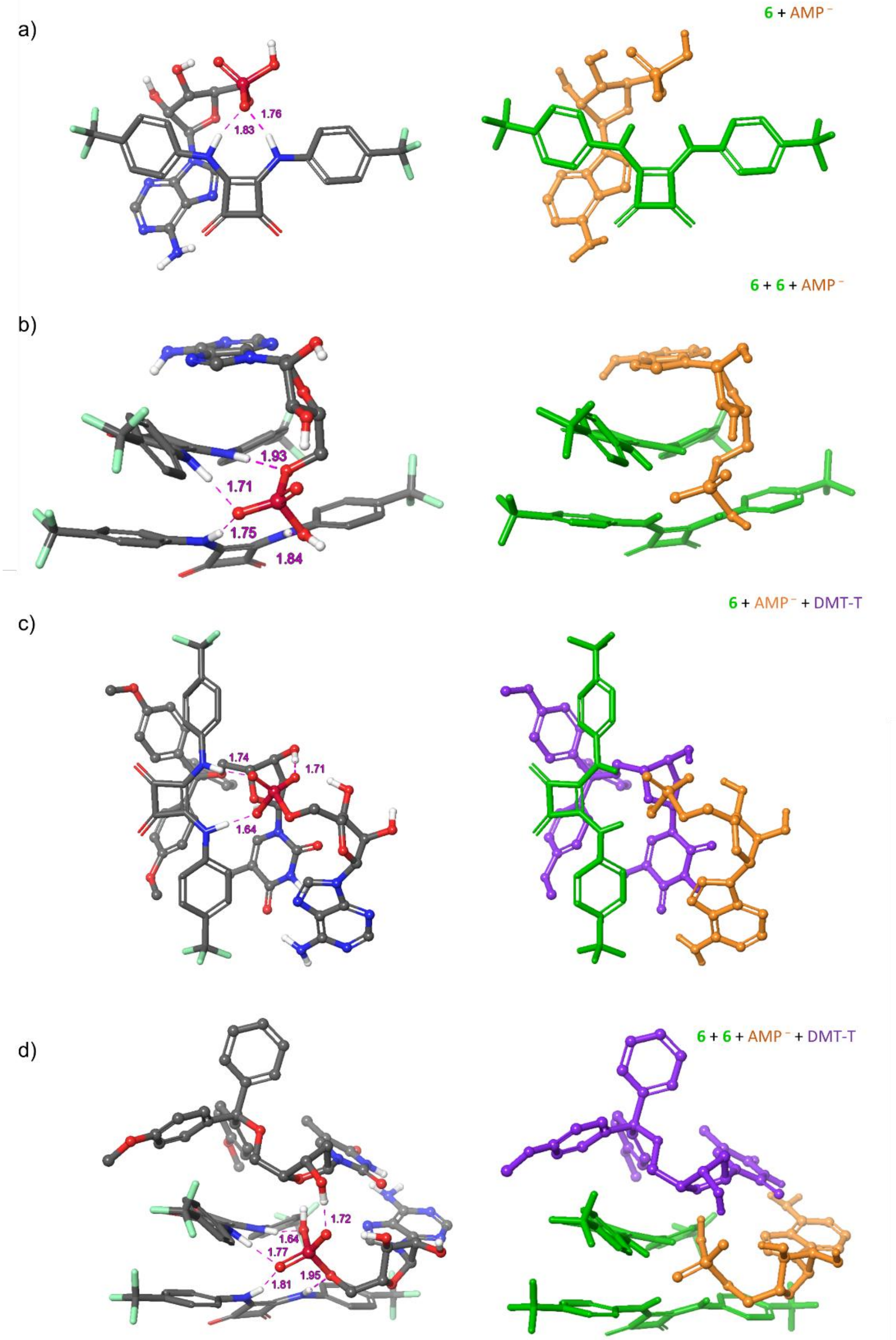


**Figure S64**. Lowest energy conformers with selected distances (Å): (a) Transporter **6** (green) with AMP⁻ (orange); (b) Two transporters of **6** (both in green) with AMP⁻ (orange); (c) Transporter **6** (green) with AMP⁻ (orange) and co-transporter **DMT-dT** (purple); (d) Two transporters of **6** (both in green) with AMP⁻ (orange) and co-transporter **DMT-dT** (purple). All hydrogen atoms linked to carbon atoms are omitted for clarity.

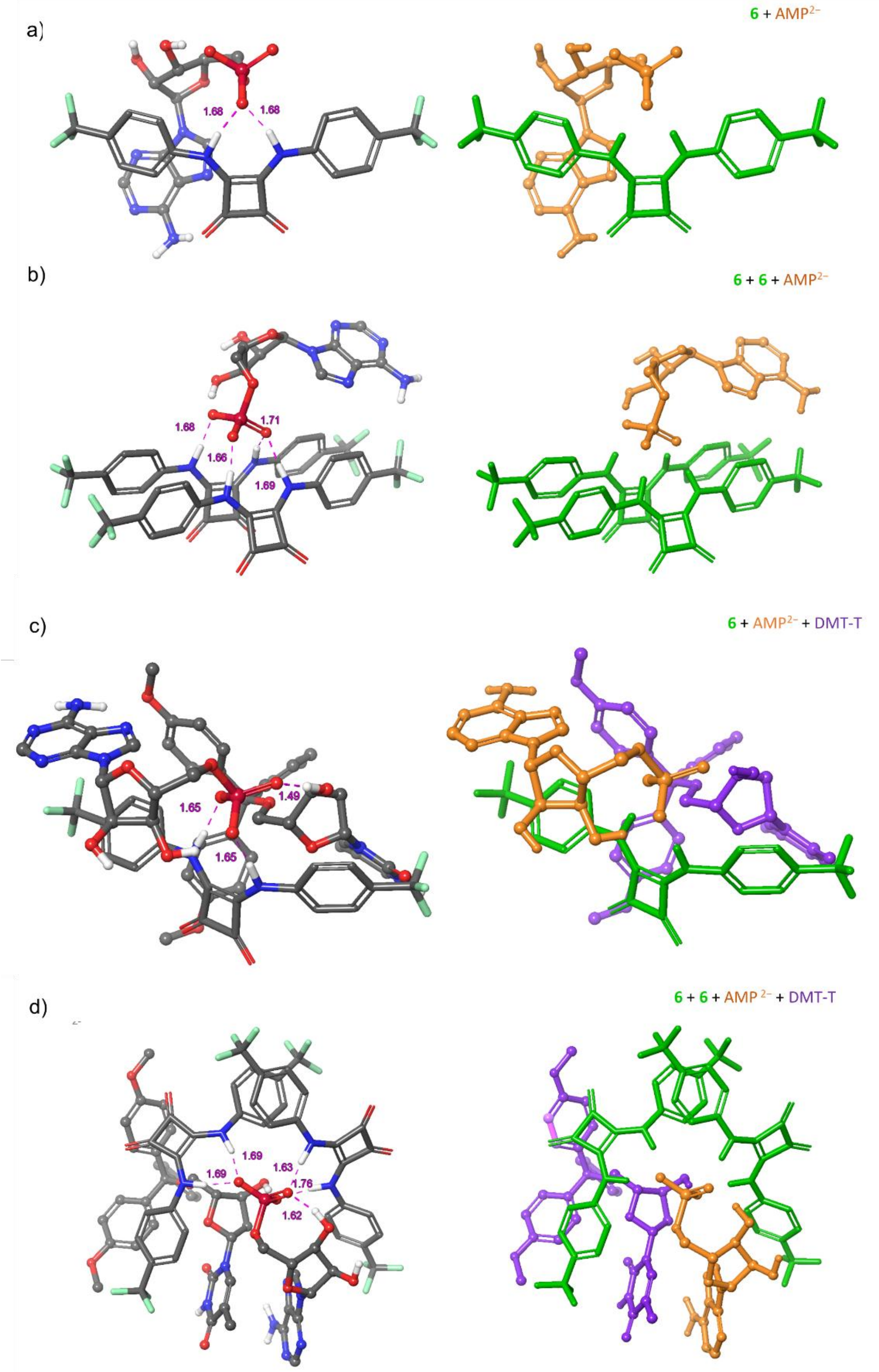


**Figure S65**. Lowest energy conformers with selected distances (Å): (a) Transporter **6** (green) with $AMP^{2-}$ (orange); (b) Two transporters of **6** (both in green) with $AMP^{2-}$ (orange); (c) Transporter **6** (green) with $AMP^{2-}$ (orange) and co-transporter **DMT-dT** (purple); (d) Two transporters of **6** (both in green) with $AMP^{2-}$ (orange) and co-transporter **DMT-dT** (purple). All hydrogen atoms linked to carbon atoms are omitted for clarity.

## S10. Evidence for co-transport mechanism

The enhanced transport observed in the presence of **DMT-dT** could be due to a general effect of **DMT-dT** on membrane properties (making it more leaky), or due to a true co-transport mechanism whereby **DMT-dT** is involved in the transporter-co-transporter-nucleotide complex. Calcein leakage assays (**section S10.1**), DPPC assays (**section S10.2**), and measurements of membrane fluidity using Laurdan and DPH (**section S10.3**) do not suggest that **DMT-dT** has a profound effect on membrane properties. A co-transporter effect of **DMT-dT** is therefore more likely. However, **DMT-dT** is able to enhance transport of other anions for most transporters, but not all (**section S10.4**), and other DMT-based nucleotides are also able to enhance the transport of cAMP (except **DMT-dG**) (**section S10.5**). Combined, these results suggest that **DMT-dT** is a true co-transporter for cAMP and AMP transport facilitated by transporters **1**-**6**, but this co-transporter event does not necessarily involve base-pairing between the thymine and adenine nucleobases and could involve other interactions such as the π-π stacking observed in the modeling above (or other types of hydrogen bonding other than Watson-Crick base pairing).

### *S10.1. Calcein leakage assay*

A thin film of POPC lipid was formed by evaporating a chloroform solution of lipid under reduced pressure, followed by drying under high vacuum for at least for 8 hours. The lipid film was hydrated by vortexing with a $NaNO_3$ solution (70 mM calcein in 225 mM $NaNO_3$, 10 mM HEPES at pH 7.4). The lipid suspension was then subjected to eleven freeze-thaw cycles alternating between submersion in liquid nitrogen followed by thawing in a mildly warm water bath (below 34 °C). The lipid suspension was allowed to rest at room temperature for 30 min and was subsequently extruded 29 times through a 200 nm polycarbonate membrane using the Avanti mini extruder set (Avanti Polar Lipids, Inc.). Unencapsulated calcein was removed by size exclusion chromatography on a Sephadex G-50 column, eluted with 225 mM $NaNO_3$, 10 mM HEPES, pH 7.4. The final lipid concentration per sample was 0.5 mM.

The dye-loaded liposomes (0.5 mM lipid) were transferred into a 3 mL glass cuvette and placed in the sample compartment of an Agilent Cary Eclipse fluorescence spectrometer equipped with a magnetic stirrer, and a temperature controller. Two minutes before starting the kinetic run, 75 µL of a sodium salt of cAMP stock solution (1 M in 225 mM $NaNO_3$ and 10 mM HEPES, pH 7.4)) was added to reach a final concentration of 25 mM. At $t$ = 0 min, the kinetic run began, and at $t$ = 1 min 15 µL of the transporter solution in DMF was added and the fluorescence emission ($\lambda_{ex}$ = 490 nm, $\lambda_{em}$ = 520 nm) of encapsulated calcein was recorded for 60 minutes. At time $t$ = 60 min, detergent (75 µL of 10% Triton X-100) was added to fully lyse the membrane. Transporter concentrations are given as mol% with respect to POPC lipid concentration. Control

experiments were conducted using DMF and **DMT-dT** (10 mol% with respect to lipid) without the transporter, serving as blank solutions. The fluorescence intensities were converted to % calcein leakage using the following equation ($F_t$ = fluorescence intensity at time *t*, $F_0$ = fluorescence intensity at time 0, and $F_{final}$ = fluorescence intensity after addition of detergent). The results are shown in **Figure S66** and show no evidence of membrane disruption or leakage.

$$\%\ calcein\ leakage = \frac{F_t - F_0}{F_{final} - F_0} \cdot 100\%$$

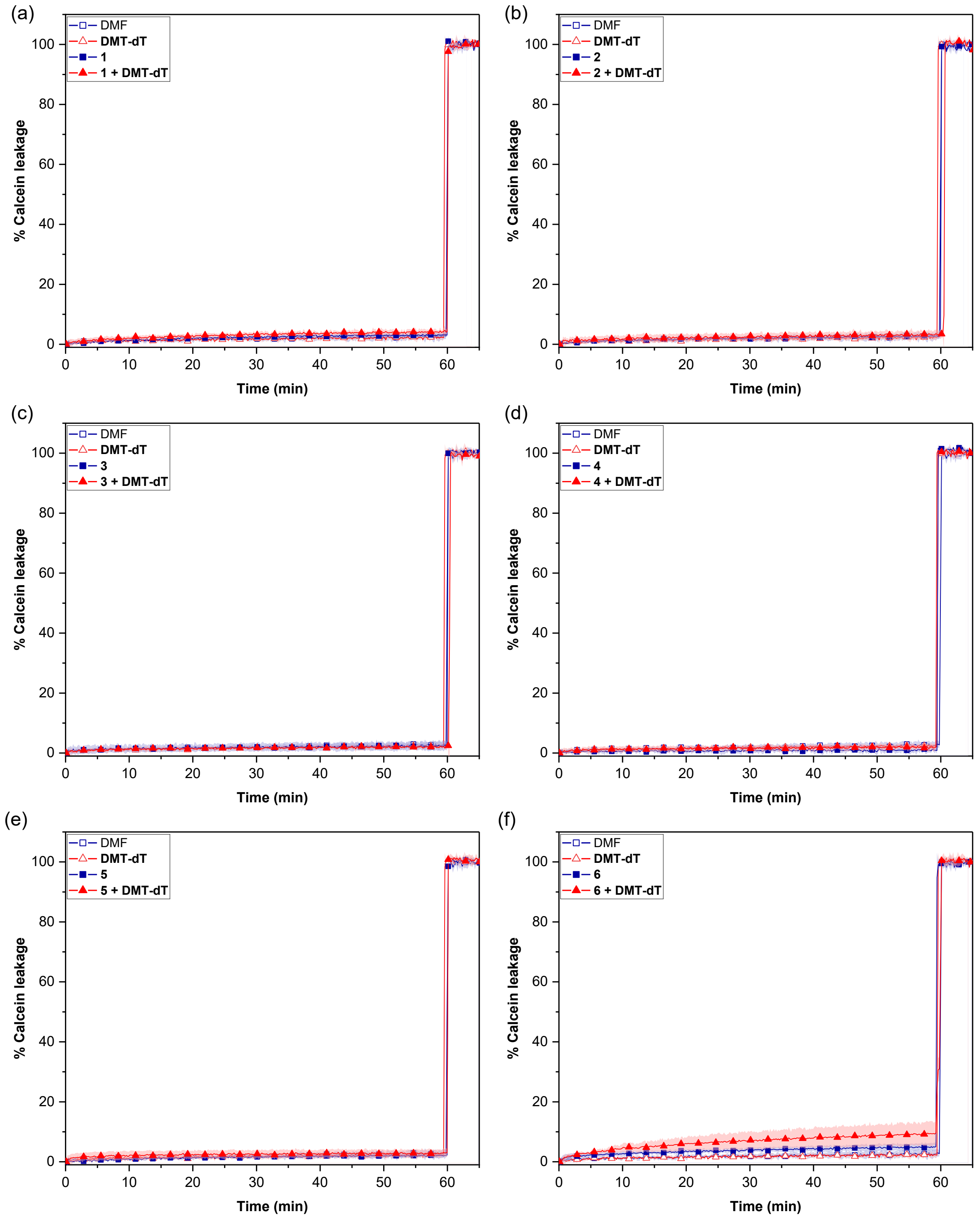


**Figure S66**. Calcein efflux promoted by transporters **1-6**, with and without **DMT-dT**. The experiment was performed as described in **section S10.1**. and is the average of minimum of 3 repeats (shaded areas represent standard deviations). Transporters **1-6** (3 mol% to lipid) with and without **DMT-dT** (10 mol% to lipid) are shown in graphs **a-j** in order.

## *S10.2. DPPC assay*

A thin film of DPPC (1,2-dipalmitoyl-*sn*-glycero-3-phosphocholine,) lipid was formed by evaporating a chloroform solution under reduced pressure, followed by drying under high vacuum for at least 8 hours. The lipid film was hydrated by vortexing with a $NaNO_3$ solution (0.8 mM SPBA in 225 mM $NaNO_3$, 10 mM HEPES at pH 7.4) The lipid suspension was then subjected to eleven freeze-thaw cycles alternating between submersion in liquid nitrogen followed by thawing in a hot water bath (~ 45 °C). The extrusion was performed at 55 °C and the lipid suspension was extruded 29 times through a 200 nm polycarbonate membrane. Unencapsulated SPBA was removed by size exclusion chromatography on a Sephadex G-25 column, eluted with 225 mM $NaNO_3$, 10 mM HEPES, pH 7.4. The final lipid concentration per sample was 0.5 mM.

The dye-loaded liposomes (0.5 mM lipid) were transferred into a 3 mL glass cuvette and placed in the sample compartment of an Agilent Cary Eclipse fluorescence spectrometer equipped with a magnetic stirrer, and a temperature controller. Stirring was initiated at maximum speed and maintained throughout the experiment. The transport experiment was conducted at two different temperatures: 25 °C (below the phase transition temperature – gel phase), and 45 °C (above the phase transition temperature – liquid crystalline phase). After placing the cuvettes in the fluorometer, they were equilibrated for 5 min at each temperature prior to initiating the experiment. Two minutes before starting the kinetic run, 75 µL of a sodium salt of cAMP sodium salt stock solution (1 M in 225 mM $NaNO_3$ and 10 mM HEPES, pH 7.4)) was added to reach a final concentration of 25 mM. At $t$ = 0 min, the kinetic run began, and at $t$ = 1 min 15 µL of the transporter solution in DMF was added to initiate the influx of cAMP and the efflux of $NO_3^-$ anions. The fluorescence intensity ($\lambda_{ex}$ = 435 nm, $\lambda_{em}$ = 505 nm) was recorded for 60 minutes. At time $t$ = 60 min, detergent (75 µL of 10% Triton X-100) was added to fully lyse the membrane and estimate the quality of the liposomes. Transporter concentrations are given as mol% with respect to DPPC concentration. Data work-up was performed as described in **section S6.1.**

The experiment was conducted only with transporter **1** and transporter **6** (3 mol% to lipid), along with **DMT-dT** (10 mol% to lipid). Control experiments were conducted using DMF and **DMT-dT** (10 mol% to lipid) without the transporter, serving as blank solutions. The results are shown in **Figure S67**. At 25 °C no transport was detected, while transport could be seen at higher temperatures. This is indicative of a mobile carrier transport mechanism, because mobile carriers cannot diffuse through the gel-like ordered phase of DPPC at low temperature (but can diffuse through the less ordered liquid-crystalline phase of DPPC above 41 °C).

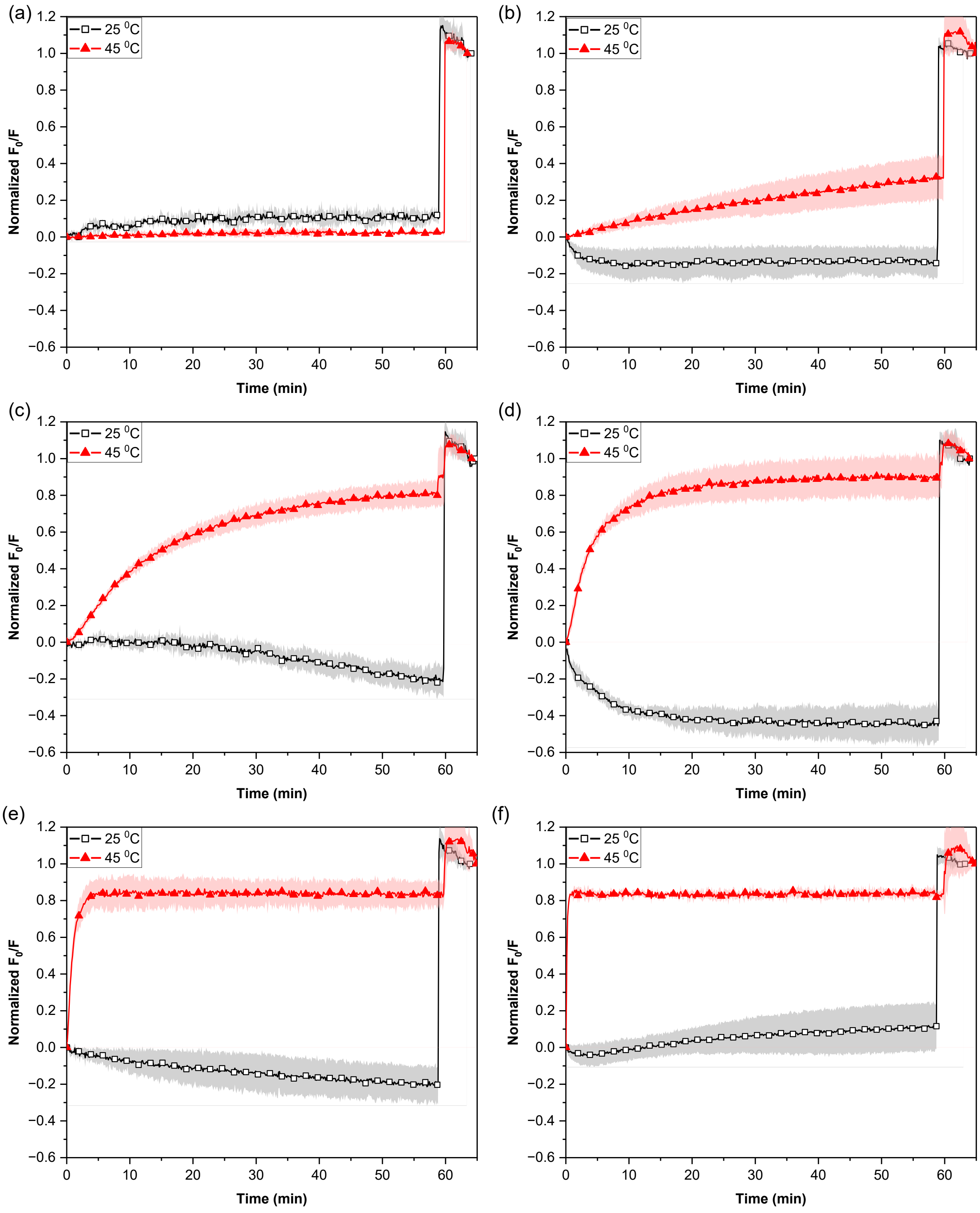


**Figure S67.** cAMP transport across 200 nm DPPC LUVs at 25 °C and 45 °C mediated by (a) **DMF**, (b) **DMT-dT** (10 mol% to lipid), (c) transporter **1** (3 mol%), (d) transporter **1** (3 mol%) with **DMT-dT** (10 mol% to lipid), (e) transporter **6** (3 mol%), and (f) transporter **6** (3 mol%) with **DMT-dT** (10 mol% to lipid). The experiment was performed as described in **section S10.2.** and is the average of minimum of 3 repeats (shaded areas represent standard deviations).

## *S10.3. Membrane fluidity measurements*

Two assays were used to measure membrane fluidity: Laurdan and DPH. Laurdan is sensitive to the polarity of its environment in the bilayer, whereby changes in water content cause a spectral shift that can be quantified as Laurdan GP. Laurdan GP has been linked to changes in phospholipid order.[8] DPH (1,6-diphenyl-1,3,5-hexatriene) is a rigid-rod type dye whose fluorescence anisotropy is correlated to membrane fluidity.[9]

### S10.3.1. Laurdan assay

A thin film of POPC lipid was formed by evaporating a chloroform solution of lipid under reduced pressure, followed by drying under high vacuum for at least for 6 hours. The resulting lipid film was then hydrated with Laurdan dye solution (1 mM in chloroform) to achieve a final composition of 1 mol% Laurdan respective to the total lipid, ensuring pre-incorporation of the dye into the lipid. The film was subsequently dried under vaccum again for at least 4 hours. The lipid film was hydrated by vortexing with a $NaNO_3$ solution (225 mM $NaNO_3$, 10 mM HEPES at pH 7.4). The lipid suspension was then subjected to eleven freeze-thaw cycles alternating between submersion in liquid nitrogen followed by thawing in a mildly warm water bath (below 34 °C). The lipid suspension was allowed to rest at room temperature for 30 min and was subsequently extruded 29 times through a 200 nm polycarbonate membrane using the Avanti mini extruder set (Avanti Polar Lipids, Inc.). The pre incorporated dye-loaded liposomes (0.5 mM lipid) were transferred into a 3 mL glass cuvette and placed in the sample compartment of an Agilent Cary Eclipse fluorescence spectrometer equipped with a magnetic stirrer, and a temperature controller. The final lipid concentration per sample was 0.5 mM. 15 µL of **DMF** or **DMT-dT** (10 mol% respective to lipid) was added to the lipid solution and stirred for 10 minutes in the fluorometer at room temperature. The emission spectrum of Laurdan was measured from 420 nm to 700 nm ($\lambda_{ex}$ = 360 nm). General polarizability (GP) values were calculated using the following equation ($I_{440}$ is the intensity of the emission at 440 nm and $I_{490}$ is the intensity of the emission at 490 nm):

$$GP = \frac{I_{440} - I_{490}}{I_{440} + I_{490}}$$

The results are shown in **Figure S68**. A small increase in Laurdan GP was observed for the addition of 10 mol% **DMT-dT** to POPC liposomes. This indicates a small increase in rigidity, and is therefore expected to be detrimental for transmembrane transport (an increase in fluidity would be expected to make the membrane more permeable, while an increase in rigidity would be expected to make the membrane less permeable). However, we also measured Laurdan GP for POPC containing 10 mol% cholesterol and for pure DPPC membranes, which are both known to be much more rigid than POPC bilayers. The effect of **DMT-dT** is not as large as that of cholesterol

or a phospholipid bilayer in the gel phase, and we therefore consider the effect of **DMT-dT** on membrane fluidity minimal.

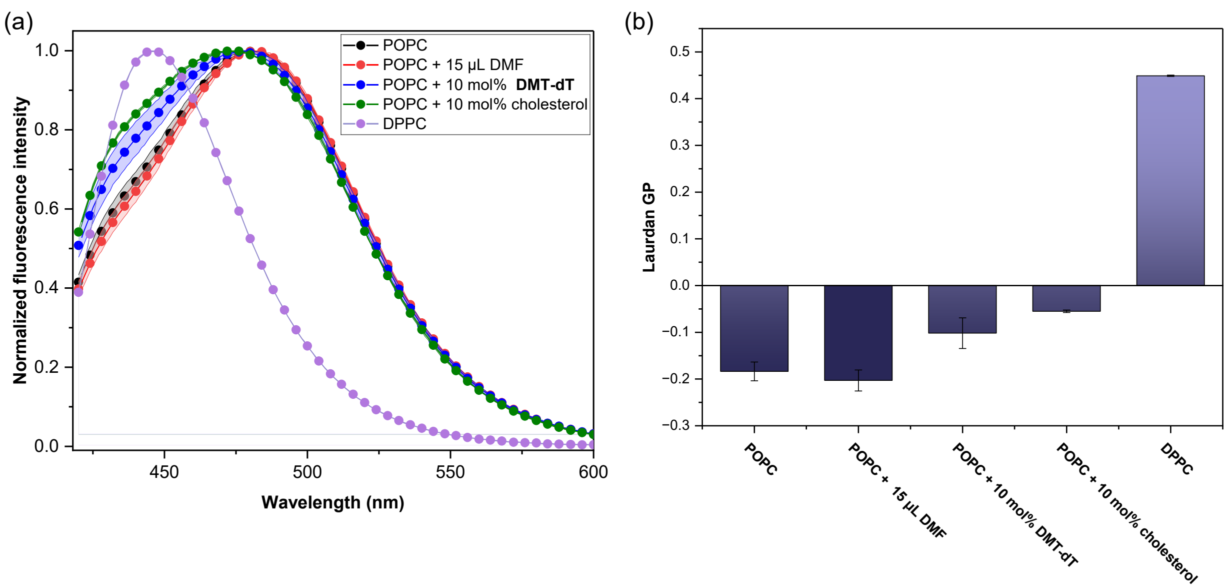


**Figure S68.** (a) Normalized emission spectra of Laurdan embedded in untreated POPC vesicles, POPC vesicles after the addition of DMF or **DMT-dT** (10 mol% respective to lipid), 1:9 cholesterol:POPC vesicles (10 mol% cholesterol), and DPPC vesicles measured using an excitation wavelength of 360 nm at 24 °C. (b) Calculated GP values using the emission intensities at 440 nm and 490 nm. The experiment was performed as described in **section S10.3.1.** and is the average of minimum of 3 repeats. Shaded areas and error bars represent standard deviations.

### S10.3.2. DPH assay

A thin film of POPC lipid was formed by evaporating a chloroform solution of lipid under reduced pressure, followed by drying under high vacuum for at least for 6 hours. The lipid film was hydrated by vortexing with a $NaNO_3$ solution (225 mM $NaNO_3$, 10 mM HEPES at pH 7.4). The lipid suspension was then subjected to eleven freeze-thaw cycles alternating between submersion in liquid nitrogen followed by thawing in a mildly warm water bath (below 34 °C). The lipid suspension was allowed to rest at room temperature for 30 min and was subsequently extruded 29 times through a 200 nm polycarbonate membrane using the Avanti mini extruder set (Avanti Polar Lipids, Inc.). The final lipid concentration per sample was 0.5 mM. The liposome solutions (0.5 mM lipid) were transferred into a 3 mL glass cuvette and placed in the sample compartment of an Agilent Cary Eclipse fluorescence spectrometer equipped with a magnetic stirrer, and a temperature controller. An aliquot of a DPH stock solution in DMF was added into the liposome solution to achieve final DPH concentration of 0.2 mol% with respect to lipid, and samples were incubated for 1 h in the fluorometer in the dark at room temperature. Then 15 μL of **DMF** or **DMT-dT** (10 mol% respective to lipid) was added to the lipid solution and stirred for another 10 minutes. The fluorescence anisotropy was measured using an Agilent Cary Eclipse

fluorometer with manual polarizer (excitation = 355 nm, emission = 425 nm). The steady state fluorescence anisotropy was then calculated with following equation:

$$anisotropy = \frac{I_{vv} - G \cdot I_{vh}}{I_{vh} + 2 \cdot G \cdot I_{vh}}$$

Where $I_{vv}$ is the vertical component of fluorescence intensity measured using excitation with vertically polarized light, and $I_{vh}$ is the horizontal component of the fluorescence intensity measured using excitation with vertically polarized light. G is a correction factor for the instrument, which was determined by the ratio of the fluorescence intensities of the horizontal and vertical components of a liposome solution without any fluorophore when excited with light that is horizontally polarized (G = $I_{hv}/I_{hh}$), and was found to be 0.71208 for our fluorometer. The results are shown in **Figure S69** and show no effect of **DMT-dT** on membrane fluidity as measured by DPH fluorescence anisotropy.

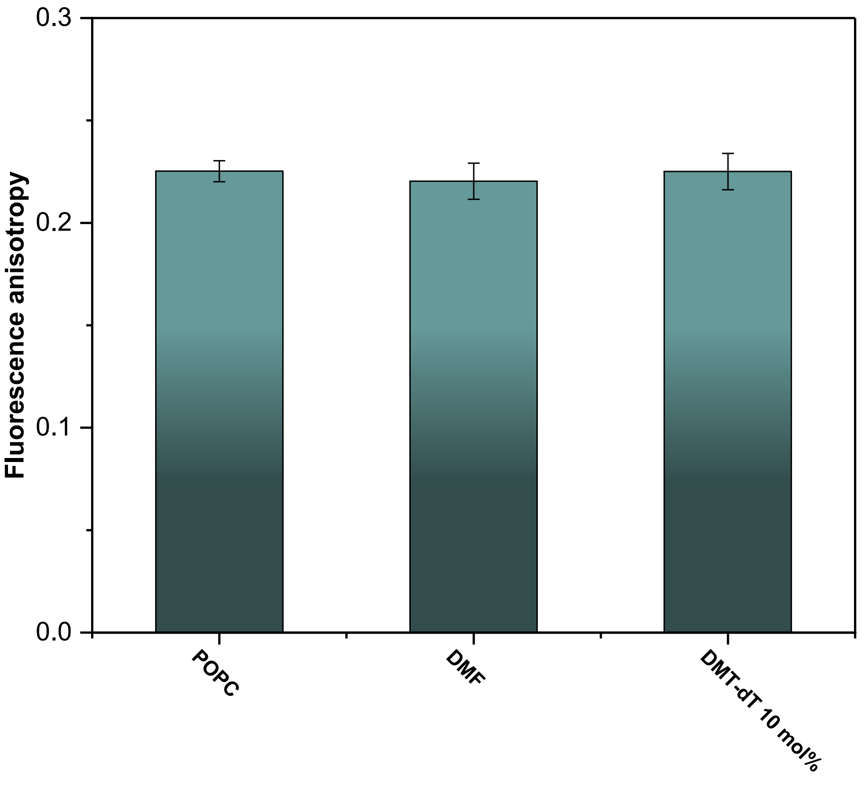


**Figure S69.** Fluorescence anisotropy of DPH in 200 nm POPC LUVs that were left untreated, or treated with 15 µL DMF or 10 mol% **DMT-dT** (10 mol% respective to lipid). The experiment was performed as described in **section S10.3.2.** and is the average of minimum of 3 repeats. Error bars represent standard deviations.

## *S10.4. Effect of DMT-dT on the transport of other anions*

### S10.4.1. Effect of **DMT-dT** on chloride transport

To investigate whether **DMT-dT** enhances the chloride transport activity of transporters **1-10** (or any transporter), the transport experiments were performed using POPC vesicles loaded with SPBA dye. Since transporters **1-10** are known chloride transporters, the experiments were carried out at concentrations close to their reported $Ec_{50}$ values in the presence of 10 mol% **DMT-dT** (10 mol% respective to lipid). To conduct this experiment, a thin film of POPC lipid was formed by evaporating a chloroform solution of lipid under reduced pressure, followed by drying under high vacuum for at least for 6 hours. The lipid film was hydrated by vortexing with a $NaNO_3$ solution (0.8 mM SPBA, 225 mM $NaNO_3$, 10 mM HEPES at pH 7.4). The lipid suspension was then subjected to eleven freeze-thaw cycles alternating between submersion in liquid nitrogen followed by thawing in a mildly warm water bath (below 34 °C). The lipid suspension was allowed to rest at room temperature for 30 min and was subsequently extruded 29 times through a 200 nm polycarbonate membrane using the Avanti mini extruder set (Avanti Polar Lipids, Inc.). Unencapsulated dye was removed by size exclusion chromatography on a Sephadex G-25 column, eluted with 225 mM $NaNO_3$, 10 mM HEPES, pH 7.4. The final lipid concentration per sample was 0.5 mM.

The dye-loaded liposomes (0.5 mM lipid) were transferred into a 3 mL glass cuvette and placed in the sample compartment of an Agilent Cary Eclipse fluorescence spectrometer equipped with a magnetic stirrer, and a temperature controller. Stirring was initiated at maximum speed and maintained throughout the experiment. At time t = 10 s, 75 µL of a sodium salt of chloride stock solution (1 M in 225 mM $NaNO_3$ and 10 mM HEPES, pH 7.4) was added. At time t = 40 s, 15 µL of the transporter solution in DMF was added to initiate the influx of chloride anions and the efflux of $NO_3^-$ anions. The fluorescence intensity (SPBA; $\lambda_{ex}$ = 435 nm, $\lambda_{em}$ = 505 nm) was recorded for 300 s. At time t = 350 s, detergent (75 µL of 10% Triton X-100) was added to fully lyse the membrane and estimate the quality of the liposomes. Transporter concentrations are given as mol% with respect to POPC lipid concentration. Data work-up was as described in **section S5.1**. The results are shown in **Figure S70** - **Figure S79**. Note that **section 10.4.2** provides further data, obtained by adding the transporters prior to the $Cl^-$ pulse, resulting in different trends (see further discussion in **section 10.4.2**).

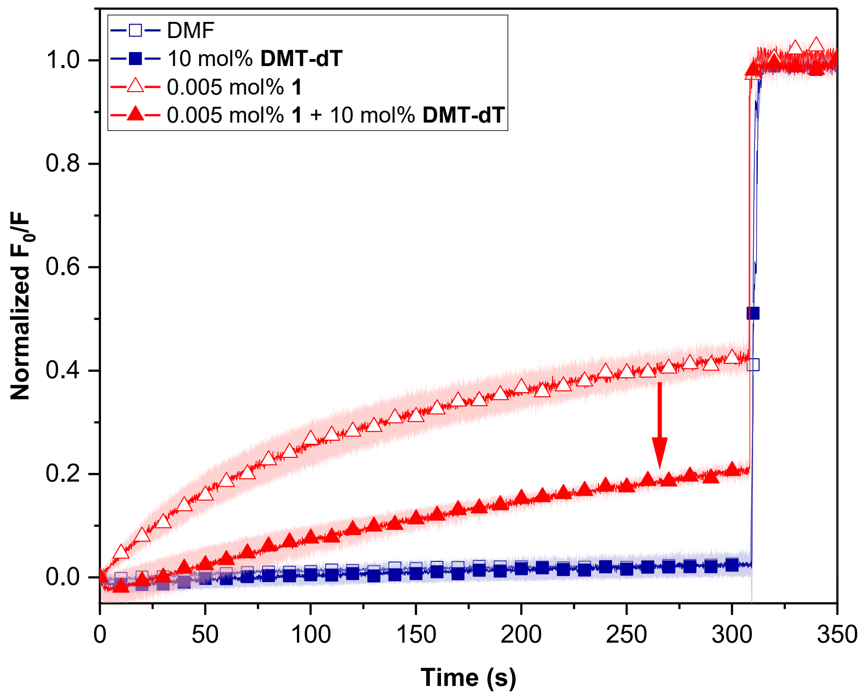


**Figure S70.** $Cl^-$ transport across 200 nm POPC LUVs mediated by transporter **1** (0.005 mol% to lipid) in the presence and absence of co-transporter **DMT-dT** (10 mol% to lipid). The experiment was performed as described in **section S10.4.1**. and is the average of minimum of 3 repeats (shaded areas represent the standard deviations).

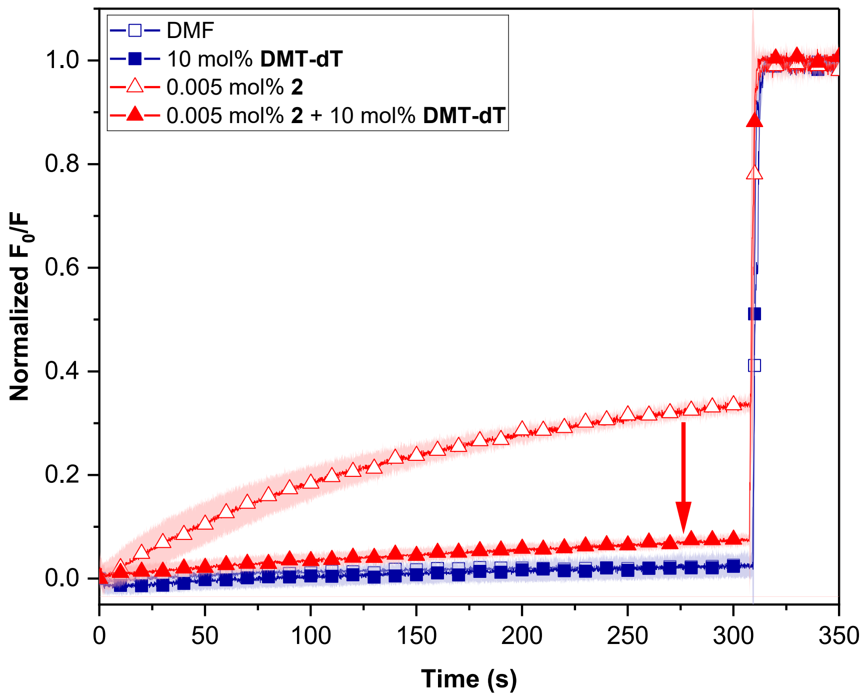


**Figure S71.** $Cl^-$ transport across 200 nm POPC LUVs mediated by transporter **2** (0.005 mol% to lipid) in the presence and absence of co-transporter **DMT-dT** (10 mol% to lipid). The experiment was performed as described in **section S10.4.1**. and is the average of minimum of 3 repeats (shaded areas represent the standard deviations).

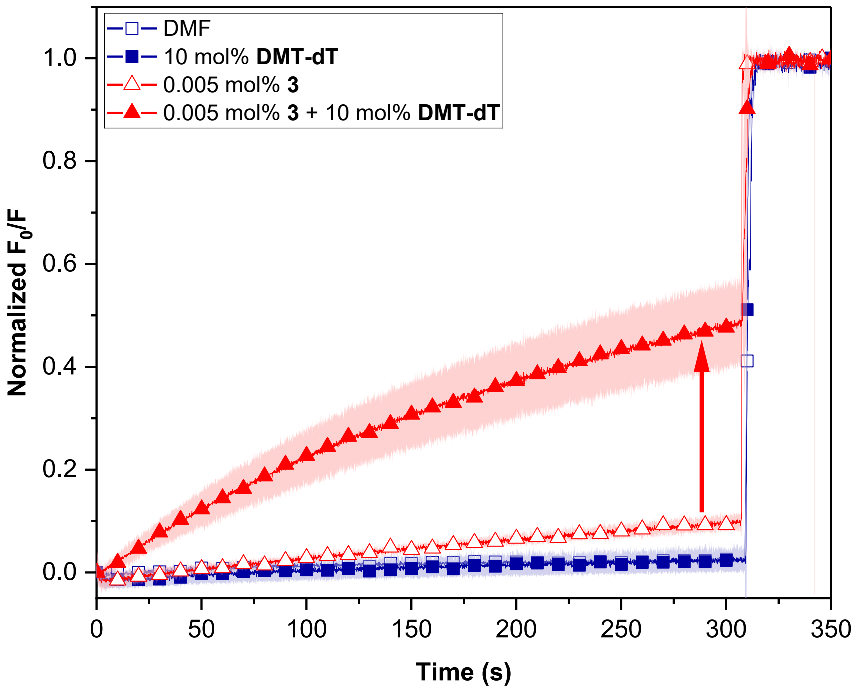


**Figure S72.** Cl⁻ transport across 200 nm POPC LUVs mediated by transporter **3** (0.005 mol% to lipid) in the presence and absence of co-transporter **DMT-dT** (10 mol% to lipid). The experiment was performed as described in **section S10.4.1**. and is the average of minimum of 3 repeats (shaded areas represent the standard deviations).

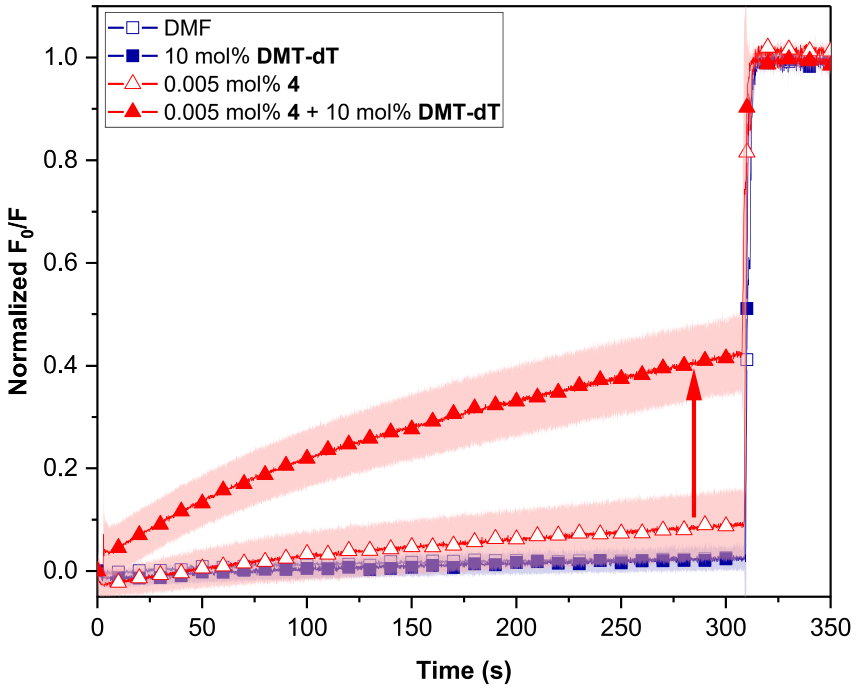


**Figure S73.** $Cl^-$ transport across 200 nm POPC LUVs mediated by transporter **4** (0.005 mol% to lipid) in the presence and absence of co-transporter **DMT-dT** (10 mol% to lipid). The experiment was performed as described in **section S10.4.1**. and is the average of minimum of 3 repeats (shaded areas represent the standard deviations).

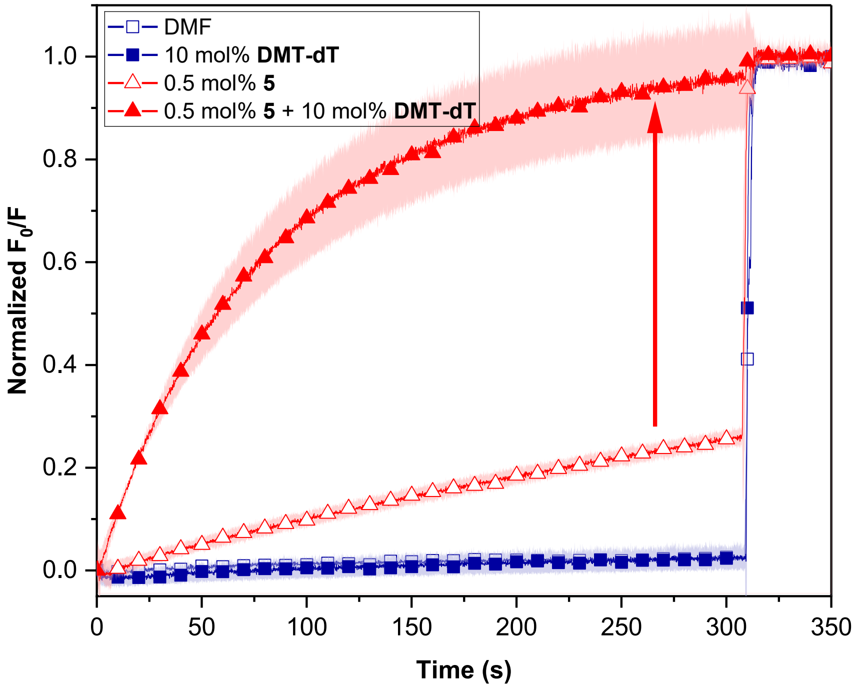


**Figure S74.** $Cl^-$ transport across 200 nm POPC LUVs mediated by transporter **5** (0.5 mol% to lipid) in the presence and absence of co-transporter **DMT-dT** (10 mol% to lipid). The experiment was performed as described in **section S10.4.1**. and is the average of minimum of 3 repeats (shaded areas represent the standard deviations).

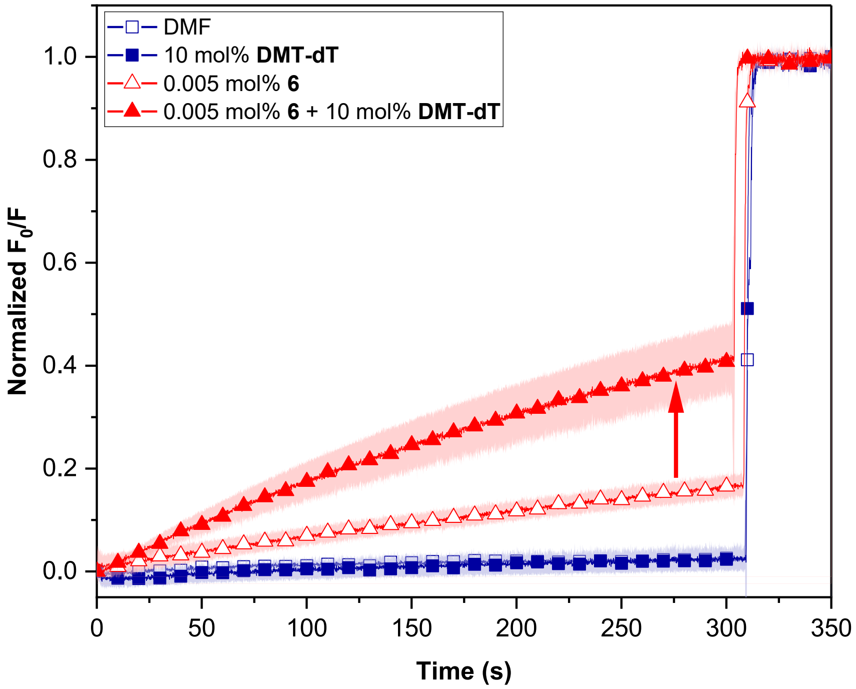


**Figure S75.** $Cl^-$ transport across 200 nm POPC LUVs mediated by transporter **6** (0.005 mol% to lipid) in the presence and absence of co-transporter **DMT-dT** (10 mol% to lipid). The experiment was performed as described in **section S10.4.1**. and is the average of minimum of 3 repeats (shaded areas represent the standard deviations).

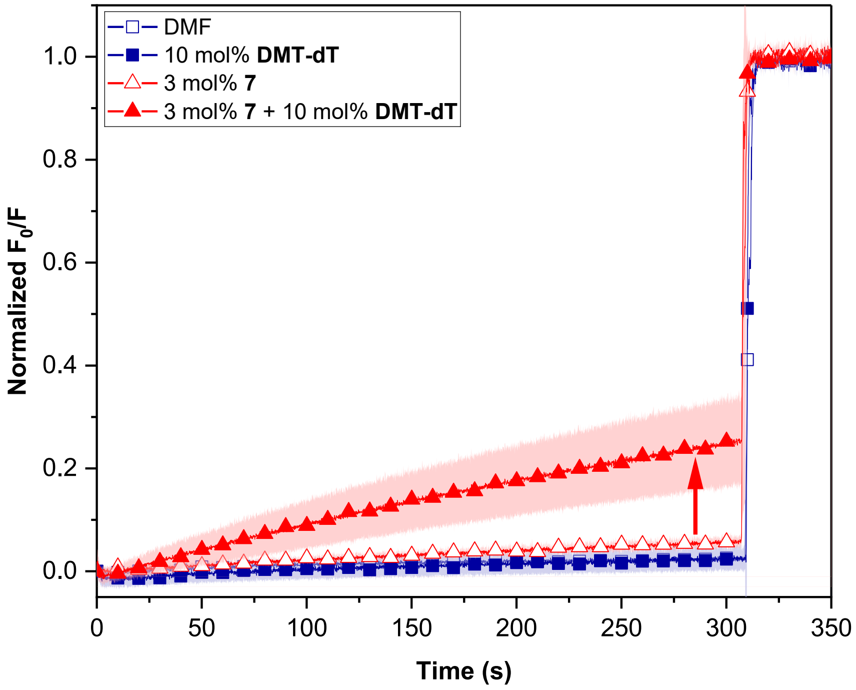


**Figure S76.** $Cl^-$ transport across 200 nm POPC LUVs mediated by transporter **7** (3 mol% to lipid) in the presence and absence of co-transporter **DMT-dT** (10 mol% to lipid). The experiment was performed as described in **section S10.4.1**. and is the average of minimum of 3 repeats (shaded areas represent the standard deviations).

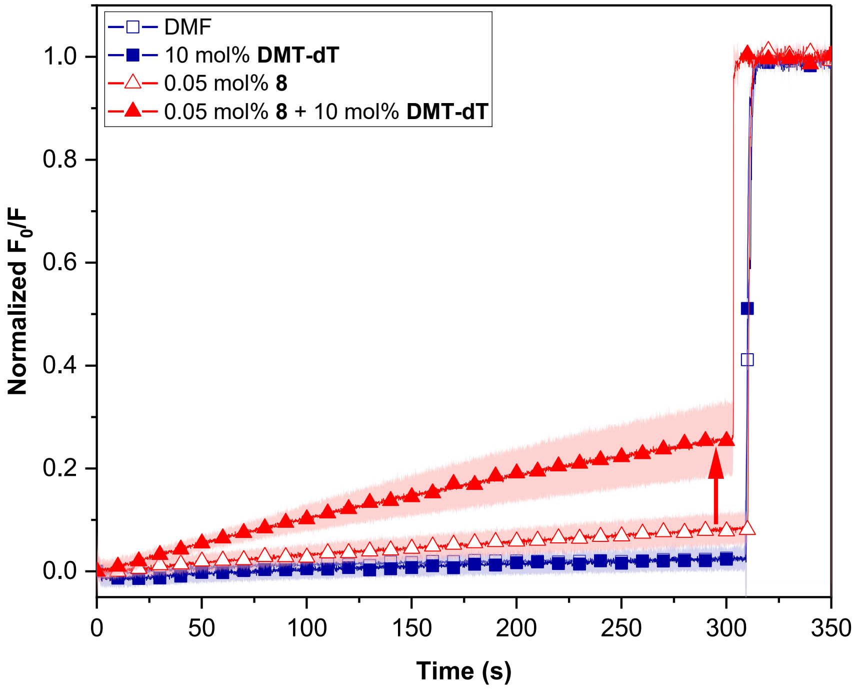


**Figure S77.** $Cl^-$ transport across 200 nm POPC LUVs mediated by transporter **8** (0.05 mol% to lipid) in the presence and absence of co-transporter **DMT-dT** (10 mol% to lipid). The experiment was performed as described in **section S10.4.1**. and is the average of minimum of 3 repeats (shaded areas represent the standard deviations).

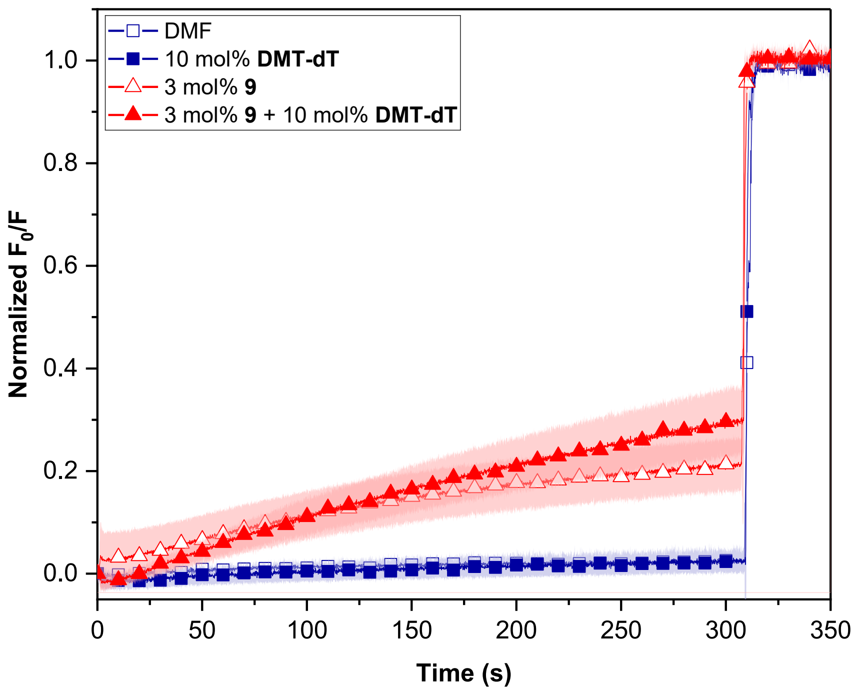


**Figure S78.** $Cl^-$ transport across 200 nm POPC LUVs mediated by transporter **9** (3 mol% to lipid) in the presence and absence of co-transporter **DMT-dT** (10 mol% to lipid). The experiment was performed as described in **section S10.4.1**. and is the average of minimum of 3 repeats (shaded areas represent the standard deviations).

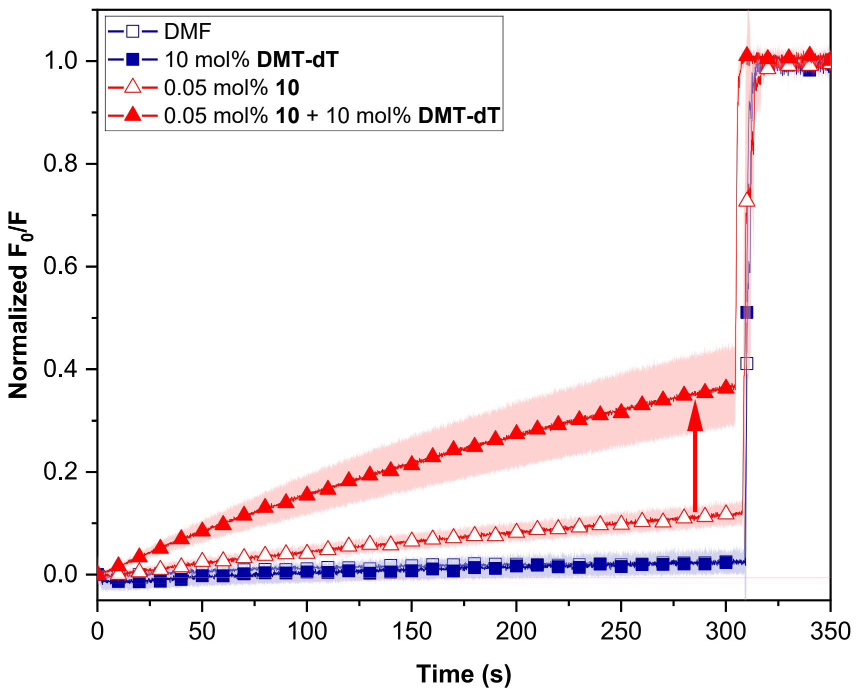


**Figure S79.** $Cl^-$ transport across 200 nm POPC LUVs mediated by transporter **10** (0.05 mol% to lipid) in the presence and absence of co-transporter **DMT-dT** (10 mol% to lipid). The experiment was performed as described in **section S10.4.1**. and is the average of minimum of 3 repeats (shaded areas represent the standard deviations).

### S10.4.2. Effect of **DMT-dT** on transporter deliverability

In most cases, the addition of **10 mol% DMT-dT** caused an increase in $Cl^-$ transport, except for transporter **9** (no effect) and transporters **1** and **2** (a decrease in $Cl^-$ transport was observed). Especially the decrease in transport observed for **1** and **2** was unexpected and could suggest deliverability issues. For the experiments shown in **Figure S70** - **Figure S79**, transporter and co-transporter are pre-mixed in DMF and this mixture is added externally to the liposome after the addition of an external $Cl^-$ pulse. In this case, if the complex between transporter and co-transporter is less soluble than transporter alone, it might take time for the transport system to diffuse into the liposomes and transport is decreased. In **section S6.1.**, we had already confirmed that there are no deliverability issues for these transporters in the cAMP transport assay. However, cAMP transport is very slow and if the transporters need a few minutes to fully incorporate into the membrane it might not be observable on a 1-hour cAMP transport assay, but it will be observable for a 5-minute $Cl^-$ transport assay.

To test this, additional experiments were performed where the transporter and **DMT-dT** were added separately and stirred for 3 min prior to the kinetic run (so that the transporters have time to diffuse into the membrane). At time $t$ = 0 s, 75 µL of a sodium salt of chloride stock solution (1 M in 225 mM $NaNO_3$ and 10 mM HEPES, pH 7.4) was added to initiate transmembrane transport. The fluorescence intensity (SPBA; $\lambda_{ex}$ = 435 nm, $\lambda_{em}$ = 505 nm) was recorded for 300 s. At time t = 300 s, detergent (75 µL of 10% Triton X-100) was added to fully lyse the membrane and estimate the quality of the liposomes. This procedure was repeated for transporters **1**, **2** and **6** and the results shown in **Figure S80** - **Figure S82**. The results confirm that **1** and **2** had deliverability issues because the decrease in $Cl^-$ transport in the presence of **DMT-dT** is not seen anymore. Overall, it therefore seems that **DMT-dT** can increase $Cl^-$ transport ability of most transporters, but not for **9** and **2**. The fact that **DMT-dT** does not have the same effect on all transporters again confirms that general membrane disruption by **DMT-dT** is unlikely. The increase in $Cl^-$ transport observed upon the addition of **DMT-dT** as a co-transporter is therefore most likely the result of direct interactions between **DMT-dT** and $Cl^-$ (via its many hydrogen bond donors) or between **DMT-dT** and the transporters.

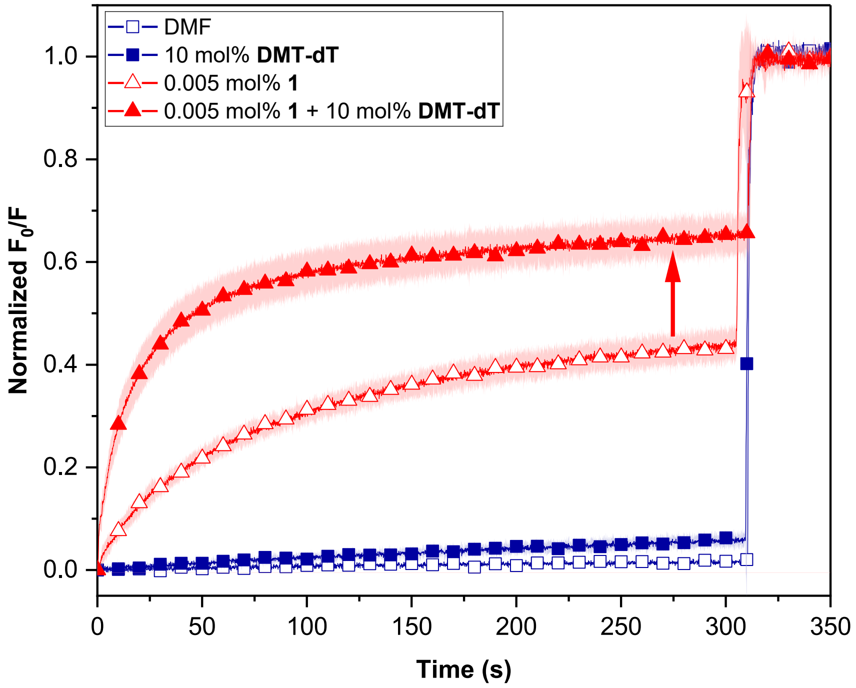


**Figure S80.** $Cl^-$ transport across 200 nm POPC LUVs mediated by transporter **1** (0.005 mol% to lipid) in the presence and absence of co-transporter **DMT-dT** (10 mol% to lipid). The experiment was performed as described in **section S10.4.2**. and is the average of minimum of 3 repeats (shaded areas represent the standard deviations).

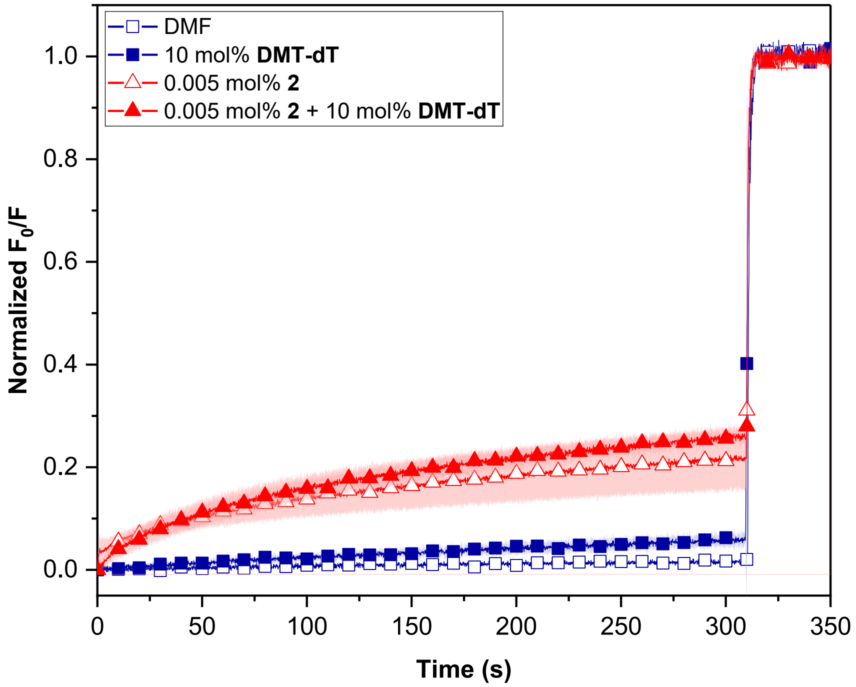


**Figure S81.** $Cl^-$ transport across 200 nm POPC LUVs mediated by transporter **2** (0.005 mol% to lipid) in the presence and absence of co-transporter **DMT-dT** (10 mol% to lipid). The experiment was performed as described in **section S10.4.2**. and is the average of minimum of 3 repeats (shaded areas represent the standard deviations).

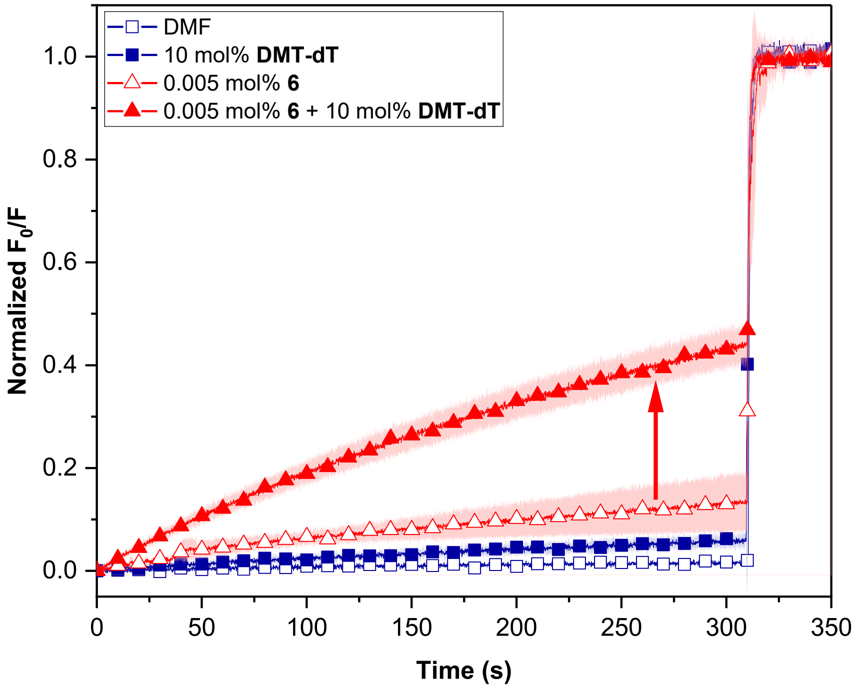


**Figure S82.** $Cl^-$ transport across 200 nm POPC LUVs mediated by transporter **6** (0.005 mol% to lipid) in the presence and absence of co-transporter **DMT-dT** (10 mol% to lipid). The experiment was performed as described in **section S10.4.2**. and is the average of minimum of 3 repeats (shaded areas represent the standard deviations).

## S10.4.3. Effect of **DMT-dT** on phosphate transport

The $^{31}P$ NMR transport studies of inorganic phosphate were carried out using the conditions previously reported.[10]

1-Palmitoyl-2-oleoyl-*sn*-glycero-3-phosphocholine (POPC) was purchased from Sigma Aldrich. A stock solution of POPC was prepared in deacidified chloroform and stored in a freezer. Volumes calculated to obtain a final lipid concentration of 50 mM in 600 µL (30 µmol) with corresponding DMF solutions of transporters **2**, **6** (12.5 µL x 24 mM to reach 1 mol%) and MeOH solution of co-transporter **DMT-dT** (125 µL x 24 mM to reach 10 mol%) were added to a 5 mL round bottom flask. The solvents were evaporated under a flow of nitrogen and dried under vacuum for at least 1 hour to obtain a lipid film. The lipid films were hydrated with 600 µL of an aqueous solution ($H_2O$) of NaCl (400 mM) and MES (5 mM) buffered at pH 5. The buffer solution was prepared in water that was deionized with a Millipore filtration system. The hydrated lipid films were sonicated for ca. 30 s and stirred for 1 hour, at room temperature to create a heterogeneous mixture of vesicles. The suspension was subjected to 10 freeze-thawing cycles to generate unilamellar vesicles and extruded 29 times through a polycarbonate membrane (200 nm pore size), at room temperature.

$^{31}P$ NMR spectra were recorded at 243 MHz on a 14.1 T Jeol JNM-ECZ600R/S3 spectrometer with a 5 mm Royal Probe. A coaxial insert containing trimethyl phosphate in $D_2O$ (50 mM) was used as reference and the chemical shift of its $^{31}P$ NMR signal was set at 3.7 ppm ($H_3PO_4$ = 0 ppm). The T1 of phosphate was determined to be 2.65 s. Using a of 45° pulse, the acquisition time and relaxation delay were set respectively to 0.84 s and 80 ms (repetition time = 0.92 seconds, equal to 0.35T1 to maximize the S/N for 1h recording time).

400 µL of liposomes were placed in an NMR tube with a coaxial insert containing trimethyl phosphate in $D_2O$ (50 mM), used as chemical shift reference (3.70 ppm) and a first $^{31}P$ NMR was recorded. No $^{31}P$ NMR signal was observed for the POPC lipid headgroups, due to the large size of the liposomes. 45 µL of a phosphate solution (1 M, $NaH_2PO_4$) prepared in the buffer used for the preparation of the liposomes was added to the liposomes sample to obtain a 100 mM phosphate extra-vesicular pulse. After 1 h, 1 µL of $MnSO_4$ (1 M, $H_2O$) was added to the liposome suspension to reach approximately 2 mM $MnSO_4$ to ensure the relaxation of the extra-vesicular phosphate $^{31}P$ NMR. After 1 hour, the spectra were recorded with 4000 scans (~1 h) to improve the signal to noise ratio and recorded again with 4000 scans (~1 h) after 10 hours.

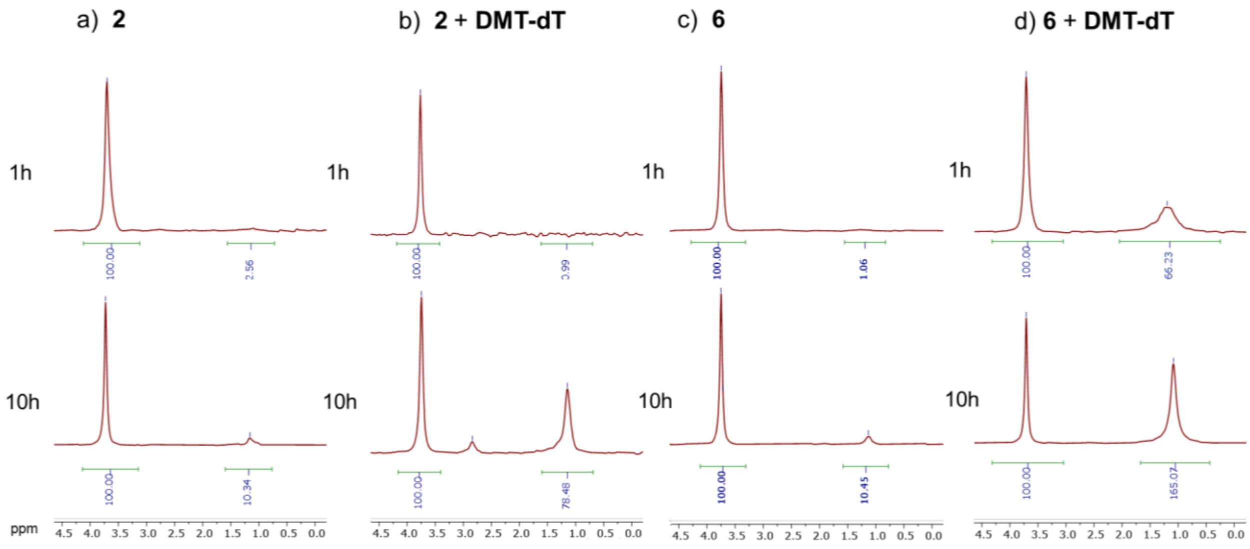


**Figure S83.** $^{31}P$ NMR spectra recorded for **$H_2PO_4^-$** transport mediated by transporters **2** and **6** with and without **DMT-dT**. The experiments were performed as described in this section. Recorded spectra for (a) transporter **2** (1 mol%) at 1 h and 10 h, (b) transporter **2** (1 mol%) with **DMT-dT** (10 mol%) at 1 h and 10 h, (c) transporter **6** (1 mol%) at 1 h and 10 h, (d) transporter **6** (1 mol%) with **DMT-dT** (10 mol%) at 1 h and 10 h. Integrations are relative to the external trimethyl phosphate peak (set to 100), but cannot be directly compared to the integrals in the $^{31}P$ NMR spectra of nucleotide transport experiments in Section 7, as those were recorded under different conditions.

## *S10.5. Effect of other DMT nucleosides on the transport of cAMP*

To investigate how other nucleosides affect the transport activity of cAMP in the presence of transporters **1-6**, experiments were conducted with the following potential co-transporters: 5'-O-(4,4'-dimethoxytrityl)-2'-deoxyadenosine (**DMT-dA**), 5'-O-(4,4'-dimethoxytrityl)-2'-deoxyguanosine (**DMT-dG**), and 5'-O-(4,4'-dimethoxytrityl)-2'-deoxycytidine (**DMT-dC**). If Watson-Crick base-pairing is involved in the co-transport mechanism, significant differences in the co-transport ability of these various DMT derivatives is expected. However, if the large lipophilic DMT group is the main driving force, the differences might be smaller (although it must be noted that for a free single nucleobase, alternative hydrogen bonds other than Watson-Crick are possible).

To conduct the experiments, a thin film of POPC lipid was formed by evaporating a chloroform solution of the lipid under reduced pressure, followed by drying under high vacuum for at least 8 hours. The lipid film was hydrated by vortexing with a $NaNO_3$ solution (0.8 mM SPBA in 225 mM $NaNO_3$, 10 mM HEPES at pH 7.4). The lipid suspension was then subjected to eleven freeze-thaw cycles alternating between submersion in liquid nitrogen followed by thawing in a mildly warm water bath (below 34 °C). The lipid suspension was allowed to rest at room temperature for 30 min and was subsequently extruded 29 times through a 200 nm polycarbonate membrane using the Avanti mini extruder set (Avanti Polar Lipids, Inc.). Unencapsulated dye was removed by size exclusion chromatography on a Sephadex G-25 column, eluted with 225 mM $NaNO_3$, 10 mM HEPES, pH 7.4. The dye-loaded liposomes (0.5 mM lipid) were transferred into a 3 mL glass cuvette and placed in the sample compartment of an Agilent Cary Eclipse fluorescence spectrometer equipped with a magnetic stirrer, and a temperature controller. Stirring was initiated at maximum speed and maintained throughout the experiment. Because transporter **1** and **2** appeared to have deliverability issues for $Cl^-$ transport in the presence of **DMT-dT** (but not for cAMP), various methods were used to add transporter, co-transporter and cAMP to the liposome solution, as detailed below (for all methods he fluorescence (SPBA; $\lambda_{ex}$ = 435 nm, $\lambda_{em}$ = 505 nm) was recorded for 60 minutes, and 75 µL of 10% Triton X-100 was added after 60 minutes to lyse the LUVs):

- Method 1: Two minutes before starting the kinetic run, 75 µL of a stock solution of the sodium salt of cAMP (1 M in 225 mM $NaNO_3$ and 10 mM HEPES, pH 7.4)) was added to reach a final concentration of 25 mM. At *t* = 0 min, the kinetic run began, and at *t* = 1 min 15 µL of the transporter/co-transporter solution in DMF was added to initiate the influx of nucleotide anions and the efflux of $NO_3^-$ anions.
- Method 2: Five minutes before starting the kinetic run, 7.5 µL of a transporter solution in DMF was added. Two minutes before starting the kinetic run, 75 µL of a sodium salt of cAMP stock solution (1 M in 225 mM $NaNO_3$ and 10 mM HEPES, pH 7.4) was added. At *t*

= 0 min, the kinetic run began, and at *t* = 1 min 7.5 µL of a cotransporter solution in DMF was added to initiate the influx of nucleotide anions and the efflux of $NO_3^-$ anions.

- Method 3**:** Three minutes before starting the kinetic run, 7.5 µL of a transporter solution in DMF and 7.5 µL of a cotransporter solution in DMF were added. At *t* = 0 min, the kinetic run began, and at *t* = 1 min 75 µL of a sodium salt of cAMP stock solution (1 M in 225 mM $NaNO_3$ and 10 mM HEPES, pH 7.4) was added to initiate the influx of nucleotide anions and the efflux of $NO_3^-$ anions.

For almost all combinations of transporter and co-transporter, no difference in transport rates were observed between the different methods of addition and the results obtained using the various methods were averaged together. This indicates that deliverability is not a big problem for the transport of cAMP due to the slow rate of transmembrane transport. The only combination that appeared to have a problem with deliverability was **1** + **DMT-dC**, which gave higher rates using method 2 and 3 (or if pre-incorporated into liposomes) than using method 1. In this case, the results obtained using method 1 were not included in the average. The final results are shown in **Figure S84 - Figure S90**. In all cases, **DMT-dG** was consistently a worse co-transporter than the other DMT derivatives. On the other hand, the differences between **DMT-dT**, **DMT-dA** and **DMT-dC** were less pronounced – but a small preference for **DMT-dT** is observed (as expected based on Watson-Crick base pairing). This suggests that base pairing could be involved in the co-transport mechanism, but other types of interactions cannot be fully excluded.

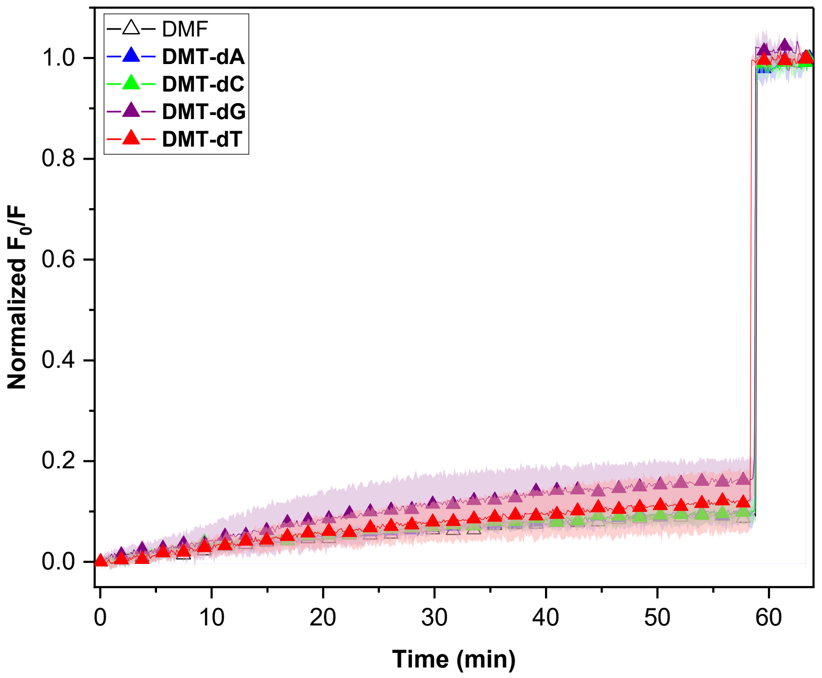


**Figure S84.** cAMP⁻ transport across 200 nm POPC LUVs mediated by **DMF** along with the different co-transporters **DMT-dA, DMT-dC, DMT-dG and DMT-dT** (10 mol% with respect to lipid). The experiment was performed as described in **section S10.5.** and is the average of minimum of 3 repeats (shaded areas represent the standard deviations).

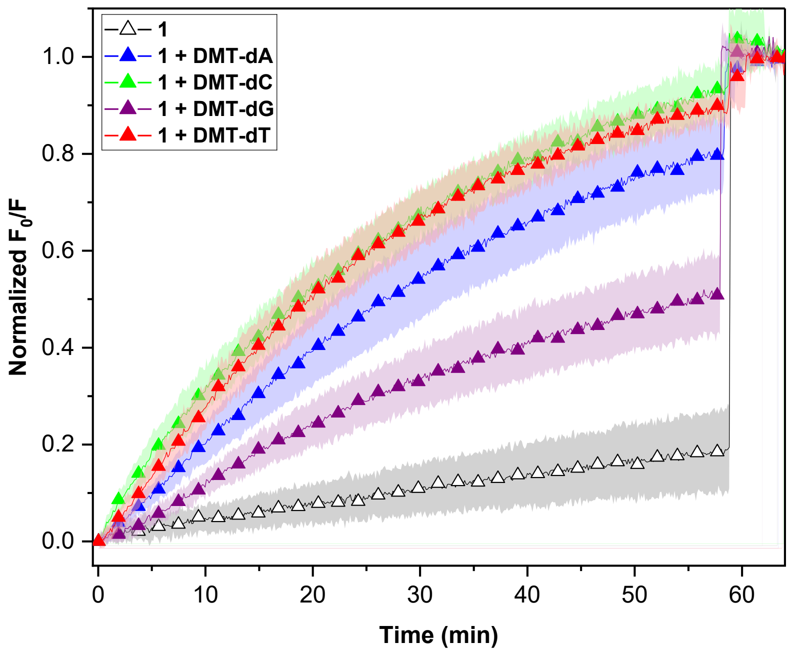


**Figure S85.** cAMP⁻ transport across 200 nm POPC LUVs mediated by **1** (3 mol%) along with the different co-transporters **DMT-dA, DMT-dC, DMT-dG and DMT-dT** (10 mol% with respect to lipid). The experiment was performed as described in **section S10.5.** and is the average of minimum of 3 repeats (shaded areas represent the standard deviations).

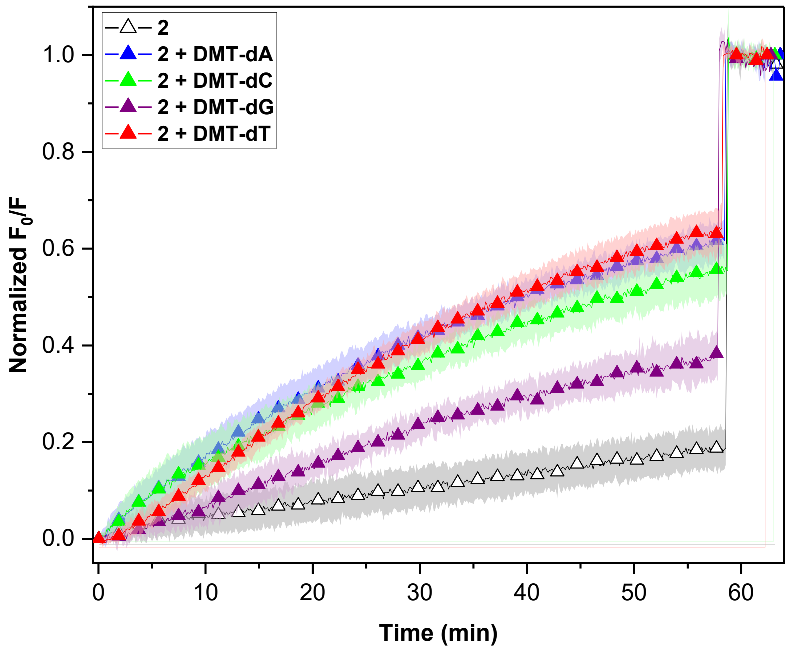


**Figure S86.** cAMP⁻ transport across 200 nm POPC LUVs mediated by **2** (3 mol%) along with the different co-transporters **DMT-dA, DMT-dC, DMT-dG and DMT-dT** (10 mol% with respect to lipid). The experiment was performed as described in **section S10.5.** and is the average of minimum of 3 repeats (shaded areas represent the standard deviations).

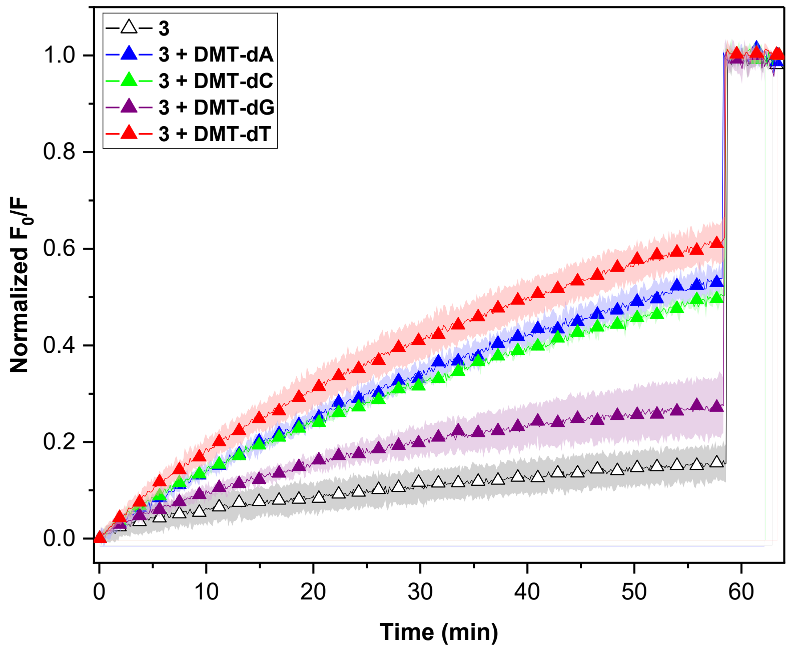


**Figure S87.** cAMP⁻ transport across 200 nm POPC LUVs mediated by **3** (3 mol%) along with the different co-transporters **DMT-dA, DMT-dC, DMT-dG and DMT-dT** (10 mol% with respect to lipid). The experiment was performed as described in **section S10.5.** and is the average of minimum of 3 repeats (shaded areas represent the standard deviations).

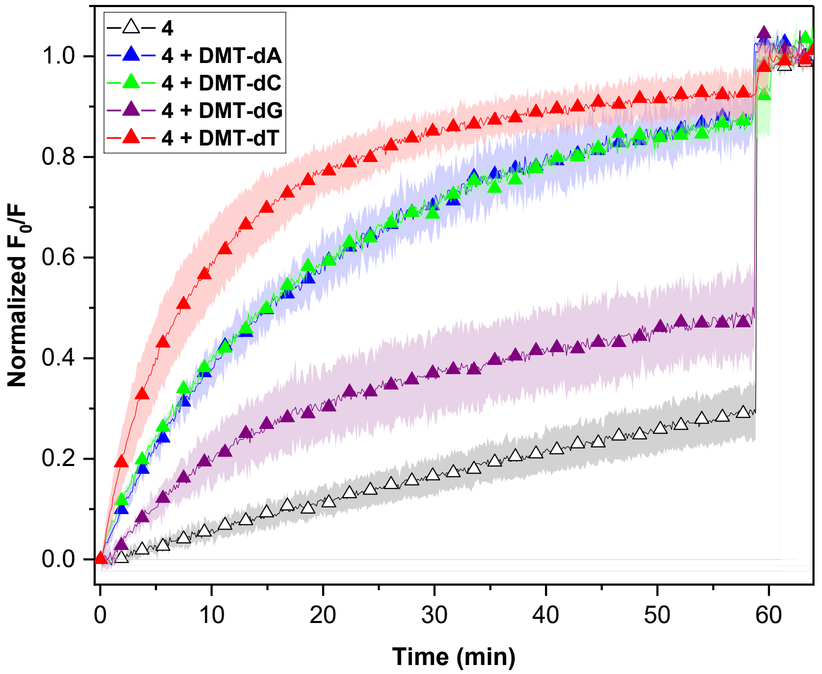


**Figure S88.** cAMP⁻ transport across 200 nm POPC LUVs mediated by **4** (3 mol%) along with the different co-transporters **DMT-dA, DMT-dC, DMT-dG and DMT-dT** (10 mol% with respect to lipid). The experiment was performed as described in **section S10.5.** and is the average of minimum of 3 repeats (shaded areas represent the standard deviations).

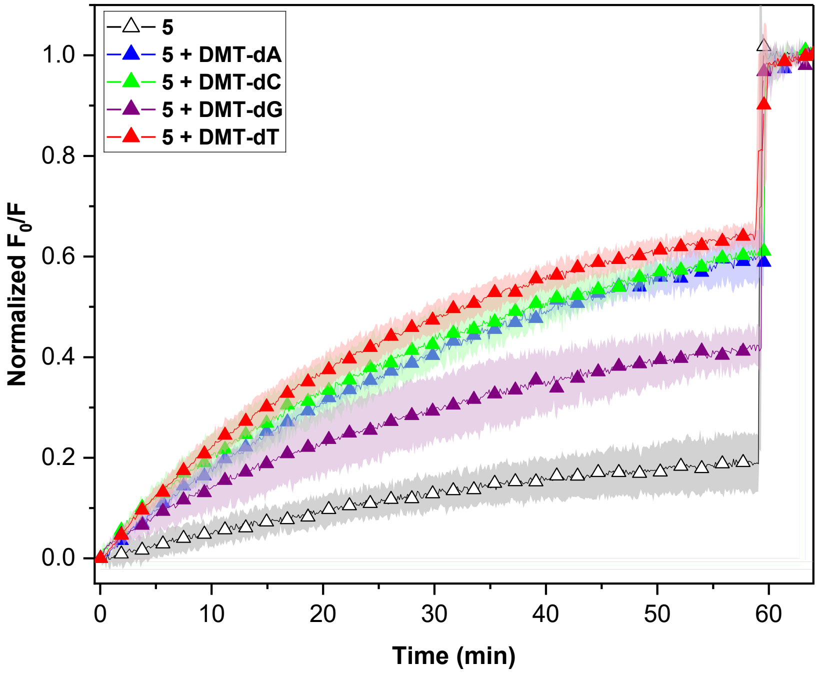


**Figure S89.** cAMP⁻ transport across 200 nm POPC LUVs mediated by **5** (3 mol%) along with the different co-transporters **DMT-dA, DMT-dC, DMT-dG and DMT-dT** (10 mol% with respect to lipid). The experiment was performed as described in **section S10.5.** and is the average of minimum of 3 repeats (shaded areas represent the standard deviations).

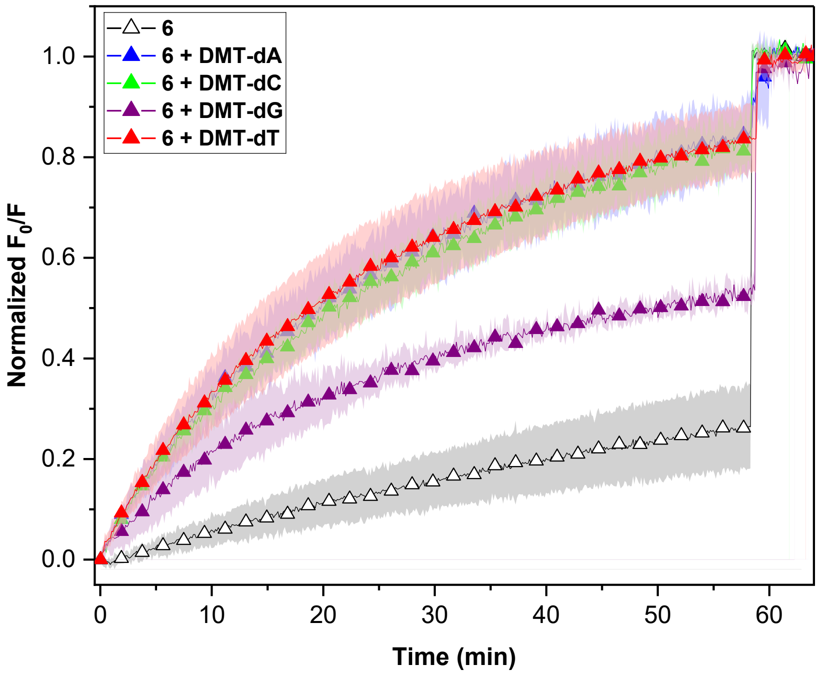


**Figure S90.** cAMP⁻ transport across 200 nm POPC LUVs mediated by **6** (0.5 mol%) along with the different co-transporters **DMT-dA, DMT-dC, DMT-dG and DMT-dT** (10 mol% with respect to lipid). The experiment was performed as described in **section S10.5.** and is the average of minimum of 3 repeats (shaded areas represent the standard deviations).

# S11. References


(1) Busschaert, N.; Wenzel, M.; Light, M. E.; Iglesias-Hernández, P.; Pérez-Tomás, R.; Gale, P. A. Structure–activity relationships in tripodal transmembrane anion transporters: the effect of fluorination. *Journal of the American Chemical Society* **2011**, *133* (35), 14136-14148.

(2) Busschaert, N.; Kirby, I. L.; Young, S.; Coles, S. J.; Horton, P. N.; Light, M. E.; Gale, P. A. Squaramides as potent transmembrane anion transporters. *Angewandte Chemie International Edition* **2012**, *51* (18), 4426-4430.

(3) Huber, C.; Fähnrich, K.; Krause, C.; Werner, T. Synthesis and characterization of new chloride-sensitive indicator dyes based on dynamic fluorescence quenching. *Journal of Photochemistry and Photobiology A: Chemistry* **1999**, *128* (1-3), 111-120.

(4) Fukushima, M.; Kikkawa, S.; Hikawa, H.; Azumaya, I. Synthesis and solvent vapor-induced transformations of crystals of 1D coordination polymers assembled with continuous void space. *CrystEngComm* **2017**, *19* (25), 3398-3406.

(5) Jaffe, E. K.; Cohn, M. 31P nuclear magnetic resonance spectra of the thiophosphate analogs of adenine nucleotides; effects of pH and Mg2+ binding. *Biochemistry* **1978**, *17* (4), 652-657. DOI: 10.1021/bi00597a014.

(6) Chvojka, M.; Singh, A.; Cataldo, A.; Torres-Huerta, A.; Konopka, M.; Šindelář, V.; Valkenier, H. The Lucigenin Assay: Measuring Anion Transport in Lipid Vesicles. *Analysis & Sensing* **2024**, *4* (2), e202300044. DOI: https://doi.org/10.1002/anse.202300044.

(7) Chang, G.; Guida, W. C.; Still, W. C. An internal-coordinate Monte Carlo method for searching conformational space. *Journal of the American Chemical Society* **1989**, *111* (12), 4379-4386.

(8) Harris, F. M.; Best, K. B.; Bell, J. D. Use of laurdan fluorescence intensity and polarization to distinguish between changes in membrane fluidity and phospholipid order. *Biochimica et Biophysica Acta (BBA) - Biomembranes* **2002**, *1565* (1), 123-128. DOI: https://doi.org/10.1016/S0005-2736(02)00514-X.

(9) He, W. DPH Probe Method for Liposome-Membrane Fluidity Determination. In *Liposomes: Methods and Protocols*, D'Souza, G. G. M., Zhang, H. Eds.; Springer US, 2023; pp 241-244.

(10) Cataldo, A.; Norvaisa, K.; Halgreen, L.; Bodman, S. E.; Bartik, K.; Butler, S. J.; Valkenier, H. Transmembrane Transport of Inorganic Phosphate by a Strapped Calixpyrrole. *J. Am. Chem. Soc.* **2023**, *145 (30)*, 16310-16314. DOI: https://doi.org/10.1021/jacs.3c04631.
